# Mechanisms of Ganweikang Tablets against Chronic Hepatitis B: A Comprehensive Study of Network Analysis, Molecular Docking, and Chemical Profiling

**DOI:** 10.1155/2023/8782892

**Published:** 2023-05-08

**Authors:** Jia-Qi Xu, Shi-Bing Su, C. Y. Chen, J. Gao, Z. M. Cao, J. L. Guan, Lin-Xuan Xiao, Ming-Ming Zhao, Hua Yu, Yuan-Jia Hu

**Affiliations:** ^1^State Key Laboratory of Quality Research in Chinese Medicine, Institute of Chinese Medical Sciences, University of Macau, Macao 999078, China; ^2^Department of Public Health and Medicinal Administration, Faculty of Health Sciences, University of Macau, Macao 999078, China; ^3^Research Center for Traditional Chinese Medicine Complexity System, Shanghai University of Traditional Chinese Medicine, Shanghai 201203, China; ^4^Jiaheng (Hengqin, Zhuhai) Pharmaceutical Technology Co., Ltd., Zhuhai, China; ^5^National Engineering Research Center for Modernization of Traditional Chinese Medicine, Zhuhai, China; ^6^Henan Fusen Pharmaceutical Co., Ltd., Henan, China

## Abstract

The hepatitis B virus (HBV) is one of the major viral infection problems worldwide in public health. The exclusive proprietary Chinese medicine Ganweikang (GWK) tablet has been marketed for years in the treatment of chronic hepatitis B (CHB). However, the pharmacodynamic material basis and underlying mechanism of GWK are not completely clear. This study is aimed at investigating the pharmacological mechanism of the GWK tablet in the treatment of CHB. The chemical ingredient information was obtained from the Traditional Chinese Medicine Database and Analysis Platform (TCMSP), Traditional Chinese Medicines Integrated Database (TCMID), and Shanghai Institute of Organic Chemistry of CAS. Ingredients and disease-related targets were defined by a combination of differentially expressed genes from CHB transcriptome data and open-source databases. Target-pathway-target (TPT) network analysis, molecular docking, and chemical composition analysis were adopted to further verify the key targets and corresponding active ingredients of GWK. Eight herbs of GWK were correlated to 330 compounds with positive oral bioavailability, and 199 correlated targets were identified. The TPT network was constructed based on the 146 enriched targets by KEGG pathway analysis, significantly associated with 95 pathways. Twenty-five nonvolatile components and 25 volatile components in GWK were identified in UPLC-QTOF/MS and GC-MS chromatograms. The key active ingredients of GWK include ferulic acid, oleanolic acid, ursolic acid, tormentic acid, 11-deoxyglycyrrhetic acid, dibenzoyl methane, anisaldehyde, wogonin, protocatechuic acid, psoralen, caffeate, dimethylcaffeic acid, vanillin, *β*-amyrenyl acetate, formonentin, aristololactam IIIa, and 7-methoxy-2-methyl isoflavone, associated with targets CA2, NFKB1, RELA, AKT1, JUN, CA1, CA6, IKBKG, FOS, EP300, CREB1, STAT1, MMP9, CDK2, ABCB1, and ABCG2.

## 1. Introduction

The hepatitis B virus (HBV) is one of the major viral infection problems in worldwide public health [1]. About 300 million people were living with chronic hepatitis B (CHB) in 2019, and around 1.5 million new infections were estimated every year globally, especially in developing countries [2]. HBV is known as a double-stranded DNA virus with a membrane and nucleocapsid (cccDNA). Widely recognized as liver damage virus, HBV is believed to be highly associated with cirrhosis and hepatocellular carcinoma (HCC) [3]. When liver cells are infected by HBV, a variety of antigens will be produced, including hepatitis B surface antigen (HBsAg), hepatitis B E antigen (HBeAg), and hepatitis B core antigen (HBcAg), resulting in a series of immune responses. Nonspecific and specific immune responses are activated successively with HBV-specific cytotoxic T cells (CTL) and cytokine production, such as interferon- (IFN-) *γ* and tumor necrosis factor- (TNF-) *α*. Additionally, a series of cell immune-related genes are affected, such as Toll-like receptors (TLRs) and retinoic acid inducible gene-1 (RIG-1), and various pathways are involved, including ROS/NF-*κ*B signaling pathway, JAK-STAT pathway, and p53 pathway [4–6]. The apoptosis of CTL and decline of cytokines result in chronic persistent infection, which serves as one of the pathogenic mechanisms of CHB [7]. Other chain reactions will further lead to the hardship of the clinical cure on CHB, such as immune tolerance, Th1/Th2 imbalance, and CTL-induced hepatocyte damage [8]. As HBV infection persists, inadequate immune responses and the undetachable virus in hepatocytes result in weakened recognition capabilities of the immune system in hosts, and the virus cannot be completely cleared. Antigen-antibody reactions on the host liver membrane induce continuously hepatocyte damage [9]. The infection will become long-standing and recurrent [10].

In China, the implementation of the hepatitis B vaccination policy in the 1990s made prominent relief on HBV prevalence; however, CHB is still a public health problem at present [11]. The dominant clinical treatments of CHB are nucleoside (acid) analogues, polyethylene glycol interferon *α* (PEG-IFN*α*) or interferon*α* (IFN*α*), and other therapies, such as anti-inflammatory, antioxidation, liver protection, and antifibrosis. The first-line drugs entecavir (ETV), tenofovir disoproxil (TDF), and tenofovir alafenamide (TAF) mainly focus on the inhibition of DNA duplication. However, long treatment cycles, drug resistance, and dosage-dependent effects cause heavy economic burdens to the patients and drag down the quality of life [12].

Traditional Chinese medicine (TCM) is a classical medical system existing over thousand years [13]. With the advantages of relatively low cost and good safety, most Chinese CHB patients have been treated with TCM therapies, especially those with CHB liver fibrosis [14]. TCMs are defined as complex systems with the natural characteristics of relatively high differentiation and personalized varieties with multiple herb combinations. Therefore, the molecular mechanisms of TCM are difficult to clarify using the same approaches in research on chemical drugs. Fortunately, network analysis may get access to the solutions on the mechanism investigation of TCM [15]. In 1999, Professor Li et al. firstly hypothesized a potential association between the biomolecular network and the “ZHENG”—the key pathological principle and medication guidance in TCM. In 2002, Li et al. revealed the “tiny and multiple” effects of traditional Chinese herbs in the treatment of complex diseases [16]. Based on computational analysis and animal experiments, Li et al. constructed a neuroendocrine–immune network to understand the basis of hot and cold ZHENG in 2007 [17]. In 2008, Professor Hopkins raised the concept of “network pharmacology” and predicted the new research mode as the next paradigm in drug discovery [18, 19]. In 2011, the “network target” was mentioned by Li et al., and the network target was used for synergistic drug combination screening in TCMs [20]. Since then, network analysis has been widely used in TCM research, and related theory, standards, and methodology guidance have gradually been established [21–23]. With network analysis, the multitarget, multifactor, and multifunction features of TCM could be fitted into nodes and edges in the network to explore the material basis and pharmacodynamic mechanism.

**Figure 1:**
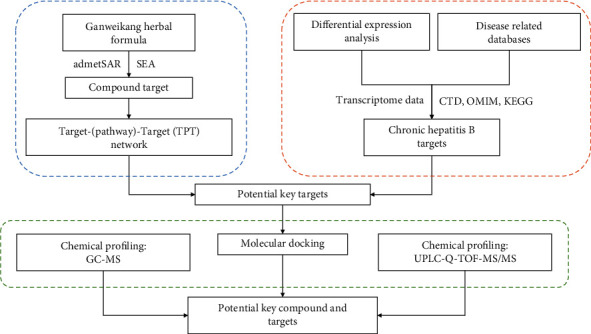
Roadmap of the study.

Ganweikang (GWK) tablets serve as an exclusive proprietary Chinese medicine that has been marketed for years. The original formulation consisted of eight herbs—Astragali Radix (Astragalus membranaceus (Fisch.) Bge. var. mongholicus (Bge.) Hsiao) (Huangqi), Verbenae Herba (Verbena officinalis L.), Stellariae Radix (Stellaria dichotoma L. var. lanceolata Bge.) (Mabiancao), Atractylodis Macrocephalae Rhizoma (Atractylodes macrocephala Koidz.) (Baizhu), Stellaria dichotoma L. var. lanceolata Bge (Yinchaihu), Saposhnikoviae Radix (Saposhnikovia divaricata (Turcz.) Schischk.) (Fangfeng), Pogostemonis Herba (Pogostemon cablin (Blanco) Benth) (Huoxiang), Forsythiae Fructus (Forsythia suspensa (Thunb.) Vahl) (Lianqiao), and Glycyrrhizae Radix et Rhizoma (Glycyrrhiza uralensis Fisch.) (Gancao). As sovereign herbs in the formulation, Huangqi has been proven to have anti-HBV effects and is commonly used in clinics, while Mabiancao is usually combined with other herbs for its anti-inflammatory and antioxidant activities [24, 25]. Glycyrrhizic acid preparation from the GWK formulation Gancao is also included in the guidelines for the prevention and treatment of CHB in China. Based on TCM theory, Huangqi combined with Mabiancao invigorates qi-flowing to strengthen the spleen, clears heat toxin, and promotes blood circulation to remove blood stasis. Huoxiang and Baizhu assist sovereign herbs in inducing diuresis to remove edema. Lianqiao and Yinchaihu help remove heat toxins. Fangfeng eliminates dampness and activates liver qi-flowing. Gancao invigorates the spleen and replenishes qi. The combination of herbs in GWK shows the effecacy of clearing heat and promoting diuresis, removing toxic substances, and promoting blood circulation for removing blood stasis. However, the complete pharmacodynamic material basis and underlying mechanism are still not clear. In this study, we focused on pharmacological basis research on GWK tablet, integrating network analysis, molecular docking, and chemical profiling including UPLC-QTOF/MS and GC-MS, aiming on the illustration of the pharmacodynamic mechanism of GWK. The workflow is showed in Figure 1.

Ultraperformance liquid chromatography coupled with a hybrid quadrupole orthogonal time-of-flight mass spectrometer (UPLC-QTOF-MS) is a technology that has been successfully used for fast and high-resolution determination of nonvolatile components and with the required sensitivity and accuracy. It has become a crucial tool for compositional analysis of complex TCM systems [26]. In addition, gas chromatography-mass spectrometry (GC-MS) technology is used for identification of volatile components in tested samples by conducting a comparative analysis on the determined mass spectrum of the individual components with those of the standard substances recorded in the well-established data library [27]. Since the GWK tablet contains both volatile and nonvolatile components, in the present study, both UPLC-QTOF/MS and GC-MS were conducted to identify the chemical compositions.

## 2. Materials and Methods

### 2.1. Data Collection and Processing

The Ganweikang (GWK) tablet consists of eight herb components—Astragali Radix (Astragalus membranaceus (Fisch.) Bge. var. mongholicus (Bge.) Hsiao) (Huangqi), Verbenae Herba (Verbena officinalis L.), Stellariae Radix (Stellaria dichotoma L. var. lanceolata Bge.) (Mabiancao), Atractylodis Macrocephalae Rhizoma (Atractylodes macrocephala Koidz.) (Baizhu), Saposhnikoviae Radix (Saposhnikovia divaricata (Turcz.) Schischk.) (Fangfeng), Pogostemonis Herba (Pogostemon cablin (Blanco) Benth) (Huoxiang), Forsythiae Fructus (Forsythia suspensa (Thunb.) Vahl) (Lianqiao), and Glycyrrhizae Radix et Rhizoma (Glycyrrhiza uralensis Fisch.) (Gancao).

#### 2.1.1. Collection of Chemical Ingredients

The chemical ingredient information of GWK was acquired from three chemical databases: Traditional Chinese Medicine Database and Analysis Platform (TCMSP, http://tcmspw.com/tcmsp.php) [28], Traditional Chinese Medicines Integrated Database (TCMID, http://www.megabionet.org/tcmid) [29], and Shanghai Institute of Organic Chemistry of CAS. Chemistry Database (http://www.organchem.csdb.cn/scdb/default.asp) [30]. Common amino acids and compounds with high molecular weights were first excluded, and then, the compounds were standardized by a canonical simplified molecular-input line-entry system (SMILES) through the PubChem database (http://pubchem.ncbi.nlm.nih.gov) [31]. Furthermore, the candidate compounds were determined by positive oral bioavailability (OB) (log*K*(%*F*) > 0) according to the admetSAR database (http://lmmd.ecust.edu.cn/admetsar2) [32].

#### 2.1.2. Chemical Ingredient Target Prediction

The relevant targets of each compound were predicted using the similarity ensemble approach (SEA, http://sea.bkslab.org/), which is a protein target prediction tool based on chemical similarity. The similarity is computed from the daylight fingerprint and involves information from the MDL Drug Data Report (MDDR) database. The ligand significances are ranked by similarity scores, and a minimum spanning tree is generated. The clusters are mapped merely based on chemical similarities; nevertheless, some of the biological sensitivities are presented as well [33]. The human targets with *Max* Tc > 0.57 were selected as candidate targets.

#### 2.1.3. Disease Target Collection

Disease-original targets were partially collected from three databases: Comparative Toxicogenomics Database (CTD, https://ctdbase.org/) [34], Online Mendelian Inheritance in Man (OMIM, http://www.omim.org) [35], and Kyoto Encyclopedia of Genes and Genomes (KEGG, http://www.genome.jp/kegg) [36] by the term “Chronic hepatitis B” or “hepatitis B”. The UniProt database (http://www.uniprot.org) [37] was used to perform protein ID standardization on each target.

### 2.2. Transcriptome Microarray Analysis

The main portion of disease-original targets was obtained from differentially expressed genes (DEGs) of the chronic hepatitis B (CHB) blood sample microarray data. In this data, we selected the transcriptome data of 10 healthy donors and 9 CHB patients from our cohort to do DEG analysis. The cDNA samples were hybridized onto the NimbleGen Homo sapiens 12 × 135K Array (Roche, CAT No. A6484-00-01). The raw data were processed using NimbleScan 2.5 software. Quantile normalization and background correction were conducted [38]. R language 3.6.1 [39] “limma” package was adopted to do DEG analysis. Packages “pheatmap” and “ggplot2” were used to obtain the heatmap and volcano map. Biological functional annotation was conducted by using KEGG pathway enrichment analysis with the R package “clusterProfiler”.

### 2.3. Network Analysis

#### 2.3.1. Target-Pathway-Target (TPT) Network Construction

The molecular mechanism of traditional Chinese medicine (TCM) is difficult to clarify due to its features of complexity and holism; however, network analysis may provide access to this solution. From the perspective of “network” and “system,” network analysis developed as a novel paradigm for the research of holistic medical system [22]. The network in TCM is based on the interactions between drugs, targets, pathways, and diseases, including the drugome network, diseasome network, and molecular interactome network. The analysis of nodes, edges, motifs, and modules serves as the underlying basis for the pathogenesis of complex diseases and the mechanism of drugs; meanwhile, it also provides indications for drug investigation as well [40].

A single target is usually associated with multiple pathways, and vice versa, one pathway is related to multiple targets. The target-pathway-target (TPT) network was constructed to delineate the target interactions in terms of related pathways. The novel network analysis approach TPT network turns two-mode “target-pathway” relationships into one-mode “target-target” interactions. Targets related to similar pathways tend to be identified in the same network module. The biological information between targets and pathways was clearly demonstrated in a single network [41]. The network of the “target-target” relationship was obtained by Pajek 5.13 software [42] from two-mode network between targets and pathways. The TPT network was visualized by Gephi 0.9.2 software [43], and modularity analysis was done by the Louvain algorithm. In the TPT network, nodes represent targets, and edges represent related pathways. Each target was assessed by a network efficiency (NE) calculation, which was defined as the total reciprocals of the shortest path lengths of each pair of targets. Then, the targets were eliminated, and new NEs were calculated. The decreased value of the new NE was called the network efficiency decrease (NED) for each specific target, which revealed the efficacy of each single target in the entire TPT network. Targets with high NED values were considered key targets. The details of the TPT network and NE calculations can be referred to in our previous work [44].

In graph theory, centrality indicators are commonly identified as the measurements of node importance in a network. In this study, we selected three centrality indicators—degree, closeness, and betweenness—as the input variables in the TPT network centrality analysis. Three centrality indicators were standardized and averaged. Targets with high normalized centrality values were considered key targets. In this study, both the NED method and centrality were implemented to assess the TPT network.

#### 2.3.2. Compound-Target-Pathway (CTP) Network Construction

A compound-target-pathway (CTP) network was constructed after the key targets were identified through the TPT network. The CTP network was conducted to illustrate the scientific basis of GWK. Based on a comprehensive network analysis, the multiple relationships between compounds, targets, and relative pathways are clearly revealed. The nodes stand for key targets, correlated compounds, and hepatitis B- (HBV-) related pathways. HBV-related pathways were defined according to the KEGG term “Hepatitis B – Homo sapiens (human).” The CTP network was visualized by Cytoscape 3.9.0 software [45].

#### 2.3.3. Statistical Verification

The association between the network module and disease targets was verified using a chi-square (*χ*^2^) test. The robustness of target rank under the NE approach in the TPT network was tested by the paired sample Wilcoxon test. Spearman's test was adopted for the correlation between the NED value in the TPT network and the frequency of CHB literature on each target. The existing literature was retrieved from the PubMed database (PubMed; http://ncbi.nlm.nih.gov) using “chronic hepatitis B” AND “target name” in the field of “title/abstract.” Spearman's correlation test was then conducted on the NED values and the literature frequencies.

### 2.4. Molecular Docking

#### 2.4.1. Protein and Ligand Preparation

The three-dimensional (3D) structures of key protein targets were downloaded from the Protein Data Bank (PDB) database (https://www.rcsb.org/) [46]. Crystal structure resolution is one of the quality standards that represents atomic position precision. Protein structures with lower resolution values should be chosen. However, resolution should not be the only selection criterion. Some high-resolution structures may lack eutectic ligands, which leads to difficulties in determining the active pocket. Thus, we chose target structures from crystal structures obtained by the X-ray diffraction method with no more than 3.0 Å resolution to the greatest extent in the PDB database. The PDB structures were prepared by removing the water ions and ligands and adding hydrogens through PyMOL 4.4 software [47] and AutoDockTools 1.5.6. software [48]. The active pockets of the structures were based on the ligand coordinates in macromolecules, while the active sites of the structures without ligands were predicted by POCASA 1.1 web version (http://altair.sci.hokudai.ac.jp) [49]. The 3D structures of the correlated active ingredients were downloaded from the PubChem database. The structures were transformed into PDB formats by Open Babel 1.1 software [50] for molecular docking.

#### 2.4.2. Protein-Ligand Molecular Docking

Molecular docking was conducted using AutoDockTools 1.5.6. software with a genetic algorithm and default parameters. Docking mode visualization and file format conversion were implemented using PyMOL 4.4 software and Open Babel 1.1 software.

### 2.5. Chemical Profiling

The compositions of volatile and nonvolatile components in GWK were separately detected by ultraperformance liquid chromatography coupled with quadrupole time-of-flight mass spectrometry (UPLC-QTOF/MS) and gas chromatography-mass spectrometry (GC-MS).

#### 2.5.1. Chemicals and Reagents

Formic acid, methanol, acetonitrile, and leucine-enkephalin of LC-MS grade were purchased from Sigma-Aldrich (St. Louis, MO, USA). Ethanol (HPLC grade) was purchased from RCI Labscan Limited (Thailand). All other reagents and chemicals were of analytical grade. Milli-Q water was prepared using a Milli-Q system (Millipore, MA, USA). Samples of volatile and nonvolatile extracts of GSW were provided by Jiaheng (Hengqin, Zhuhai) Pharmaceutical Technology Co., Ltd.

#### 2.5.2. UPLC-QTOF/MS

The nonvolatile components in GWK were analyzed on a Waters ACQUITY-UPLC CLASS system (Waters Corp., Milford, USA) with an ACQUITY UPLC HSS T3 column (150 mm × 2.1 mm, 1.8 *μ*m) (Waters Corp., Milford, USA) maintained at 35°C. Elution was performed with a mobile phase of A (0.1% FA in water) and B (0.1% FA in ACN) under a gradient program: 0-25 min, 5%–50% B, and 25-45 min, 50-100% B. The flow rate was 0.4 mL/min, and the injection volume was 5 *μ*L.

Mass spectrometric detection was carried out using a quadrupole time-of-flight (QTOF) SYNAPT G2Si High-Definition Mass Spectrometer (Waters Corp., Milford, USA) with an electrospray ionization (ESI) interface. The positive and negative ion modes were used with the mass range setting at m/z 50-1500 Da. The optimized ionization conditions were set as follows: capillary voltage of 3.0 kV, source temperature of 120°C, desolvation gas flow of 900 L/h, desolvation temperature of 450°C, cone voltage of 40 V, cone gas flow of 10 L/h, the trap collision energy of 4.0 V, transfer collision energy of 2.0 V, and collision energy ramp of 30 eV to 60 eV. A scan time of 0.2 s with an interval scan time of 0.1 s was used throughout the detection process. Moreover, leucine-enkephalin (m/z 556.2771, [M+H]+) solution, at a concentration of 200 ng/*μ*L with the flow rate of 10 *μ*L/min, was used as the external reference to ensure accuracy during the MS analysis. Data were collected using the TOF MSE (MS at elevated fragmentation energy) mode, in which parent ions and fragment ion mass spectral data were collected for each detected analyte in a single chromatographic run.

#### 2.5.3. GC-MS

The volatile components in GWK were analyzed on a column (30 m × 0.32 mm × 0.25 *μ*m) of Agilent J&W GC DB-1 (Agilent, USA) by the Thermo Trace 1300 gas chromatography system coupled with Thermo ISQ LT single quadrupole mass spectrometer (Thermo Fisher, USA). The program was as follows: the column temperature was increased with a rate of 10°C/min from 50°C to 80°C (held for 2 min), 5°C/min to 140°C (held for 1 min), and 20°C/min to 280°C (held for 1 min) and then reduced with a rate of 50°C/min to 50°C (held for 2 min). The injector temperature was maintained at 280°C with splitless injection (25 : 1) and helium (purity 99.99%) as the carrier gas at a flow rate of 1.0 mL/min. The mass spectrometer was performed at a scan range of 50-500 m/z with 70 eV ionization energy and applied 280°C and 200°C of ion source and quadruple temperature, respectively. Identification of the detected peaks was conducted by comparing the mass spectrum with the individual peak to those in the NIST MS Search 2.2 standard spectral library.

## 3. Results

### 3.1. Differentially Expressed Gene (DEG) Analysis in Microarray Data

**Figure 2:**
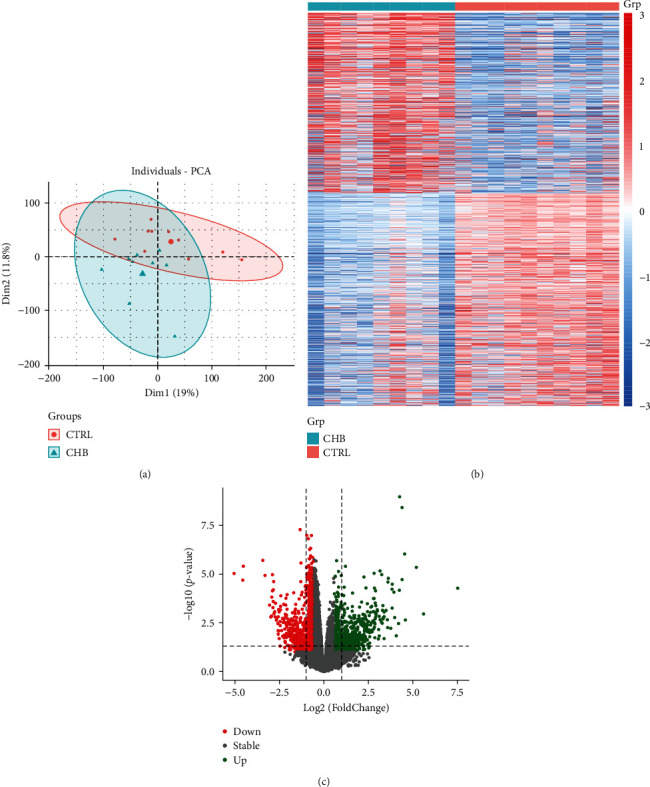
Differential expression analysis (DEG) in CHB microarray data. (a) PCA plot for CHB and control groups. (b) Heatmap for differential expression genes in the CHR and control groups. (c) Volcano map for the CHB and control groups.

Based on the transcriptome data of 10 healthy donors and 9 CHB patients, 20033 genes were obtained after data processing and cleaning, among which a total of 1129 differentially expressed genes (DEGs) were identified (*p* value < 0.05, fold change > 1.5), including 497 upregulated genes and 632 downregulated genes (Table S1). Data quality was shown by a PCA plot (Figure 2(a)). The heatmap and volcano map of the DEGs are presented in Figures 2(b) and 2(c). Disease-related targets were defined as the combination of DEGs in CHB transcriptome data and the CTD, OMIM, and KEGG databases of HBV-related targets. The total disease-related target number was 1470.

### 3.2. Target-Pathway-Target (TPT) Network

**Figure 3:**
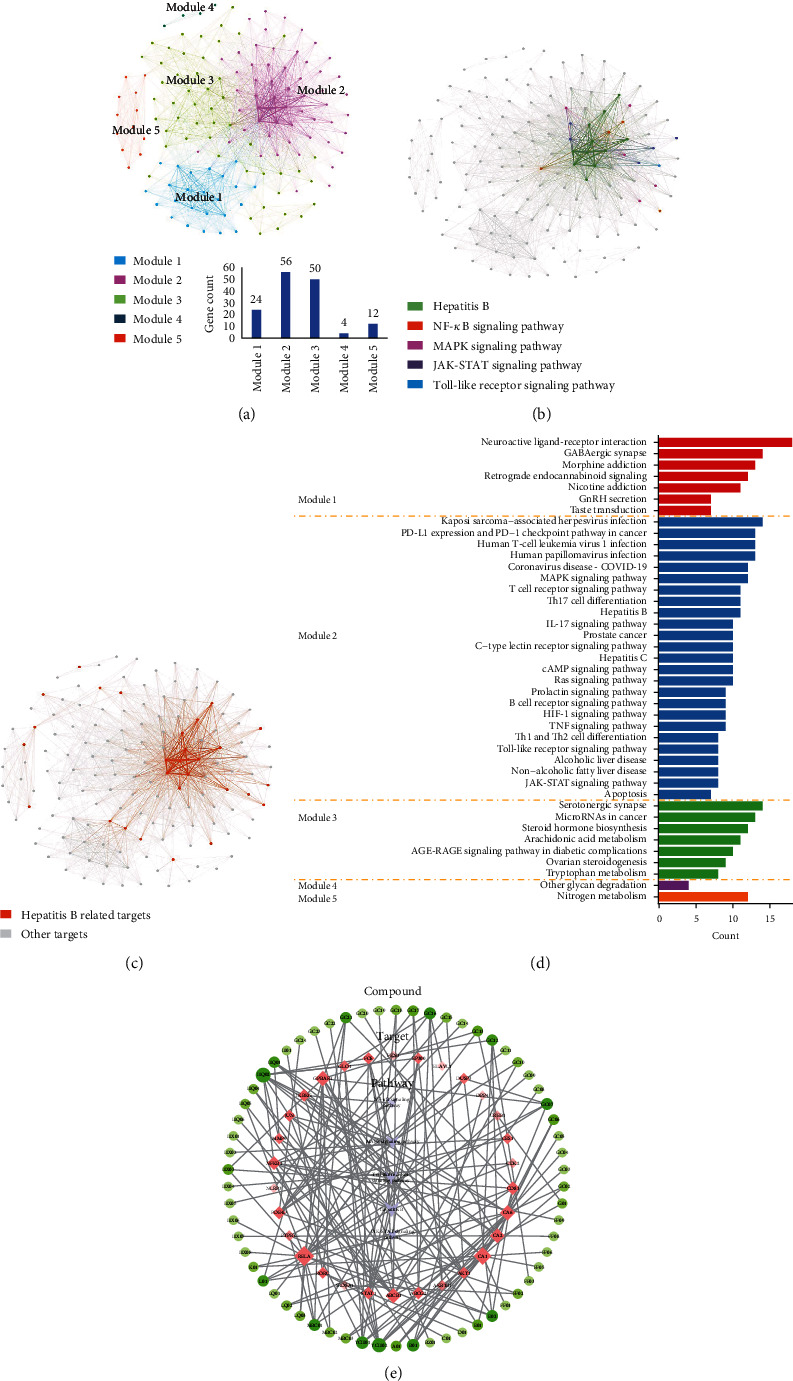
Network analysis and molecular docking for Ganweikang herbs. (a) TPT network module analysis. (b) Distribution of HBV-related signaling pathways in TPT network. (c) Distribution of HBV-related targets in TPT network. (d) KEGG pathway enrichment analysis in each TPT network module. (e) Compound-target-pathway (CTP) network, green nodes represent compounds, red nodes represent targets, and lavender nodes represent HBV-related pathways.

Eight herbs of GWK were correlated with 972 compounds, of which 330 compounds with positive oral bioavailability and 199 targets were predicted by the SEA approach (Table S2). KEGG pathway analysis enriched 146 targets, significantly associated with 95 pathways (*q*-value < 0.05). A TPT network was constructed based on 146 enriched targets (Figure 3(a)). Five modules were identified using the Louvain algorithm incorporated into Gephi software. Most of the targets enriched in HBV-related signaling pathways belonged to module 2 (Figure 3(b)), while disease-related targets showed a similar pattern (Figure 3(c)). The chi-square (*χ*^2^) test (*p* value = 0.01) result indicates an association between disease-related targets and module 2 as well. Different modules in the TPT network present diversified functions in the KEGG pathway enrichment analysis (Figure 3(d) and Table S3). HBV and related pathways, such as the “MAPK signaling pathway,” “Toll-like receptor signaling pathway,” and “JAK-STAT signaling pathway,” were enriched in module 2. Targets in other modules were enriched in different aspects of the pathways. Module 1 was associated with neurocorrelated pathways, such as the “neuroactive ligand-receptor interaction” and the “retrograde endocannabinoid signaling pathway.” Module 3 was related to metabolism, such as “arachidonic acid metabolism” and “tryptophan metabolism.” Modules 4 and 5 were relatively small and enriched in pathways such as “other glycan degradation” and “nitrogen metabolism.” Based on these results, module 2 was considered a major module in the network. Key targets were determined using the NED method (Table 1) and centrality analysis (Table 2). There was no significant difference in top target identification under the two assessment methods (*p* value = 0.314). Spearman's test showed a significant correlation between NED values and literature frequencies associated with CHB (*p* value = 0.001), which is better than the results of centrality analysis (*p* value = 0.05). Thus, CA2, NFKB1, RELA, AKT1, JUN, CA1, CA6, IKBKG, FOS, EP300, CREB1, STAT1, MMP9, CDK2, ABCB1, and ABCG2 were identified as key targets.

### 3.3. Compound-Target-Pathway (CTP) Network

The CTP network was constructed based on HBV-related pathways: “Hepatitis B,” “MAPK signaling pathway,” “NF-kappa B signaling pathway,” “Toll-like receptor signaling pathway,” and “JAK-STAT signaling pathway.” Thirty-three correlated targets and 68 compounds were included (Figure 3(e)). The importance of ingredients was investigated based on the connections between targets and pathways in the CTP network for further studies. The node information of the CTP network is shown in Table S4.

### 3.4. Molecular Docking

**Figure 4:**
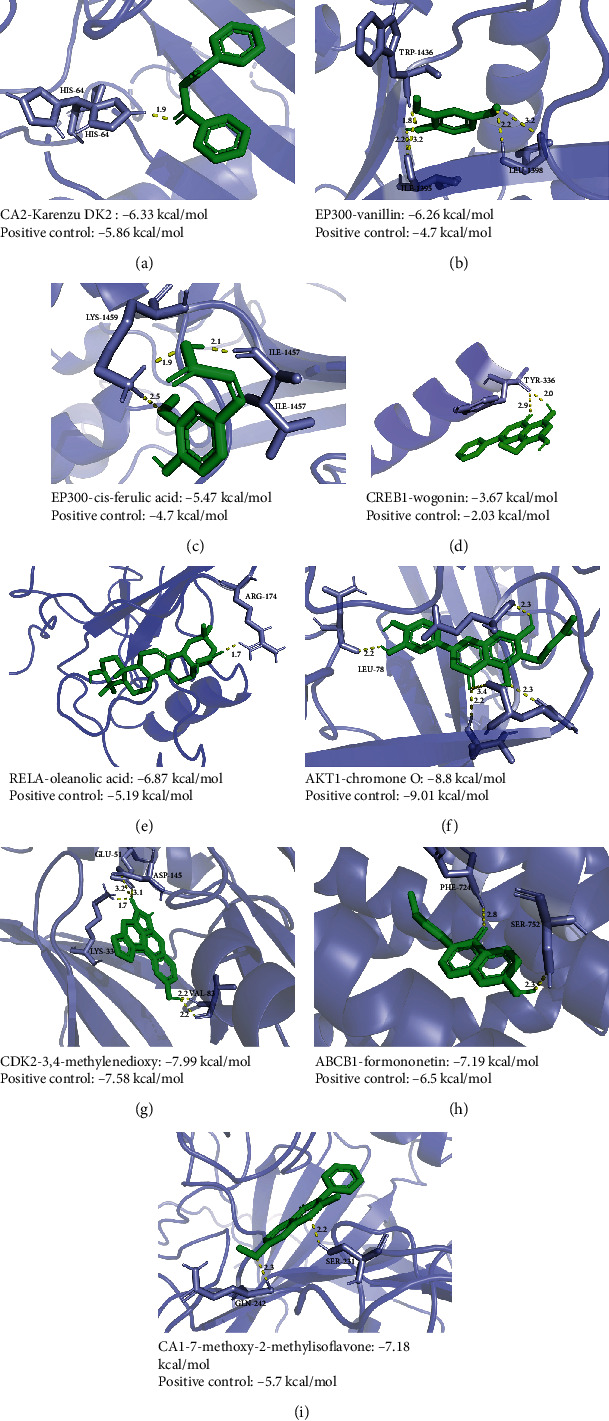
Schematic diagrams for the binding modes between active ingredients and targets: (a) CA2 with karenzu DK2 (DBM); (b) EP300 with vanillin; (c) EP300 with *cis*-ferulic acid; (d) CREB1 with wogonin; (e) RELA with oleanolic acid; (f) AKT1 with chromone O; (g) CDK2 with 3,4-methylendioxy; (h) ABCB1 with formononetin; (i) CA1 with 7-methoxy-2-methylisoflavone.

Sixteen key targets implemented molecular docking with 43 associated active ingredients (Table 3). In our study, most of the structures could meet our standards obtained by the X-ray diffraction method with no more than 3.0 Å resolution except ABCG2 (electron microscope method). Stronger or close affinities were presented between some of the targets and correlated ingredients compared with positive control compounds, such as karenzu DK2 with CA2 (-6.33 kcal/mol) (Figure 4(a)), EP300 with vanillin (-6.26 kcal/mol) (Figure 4(b)) and *cis*-ferulic acid (-5.47 kcal/mol) (Figure 4(c)), wogonin with CREB1 (-3.75 kcal/mol) (Figure 4(d)), oleanolic acid with RELA (-7.61 kcal/mol) (Figure 4(e)), chromone O with AKT1 (-8.8 kcal/mol) (Figure 4(f)), 3,4-methylenedioxy-10-hydroxy aristololactam (aristololactam IIIa) with CDK2 (-7.99 kcal/mol) (Figure 4(g)), formonentin with ABCB1 (-7.19 kcal/mol) (Figure 4(h)), and 7-methoxy-2-methylisoflavone with CA1 (-7.18 kcal/mol) (Figure 4(i)). According to the docking results, the key ingredients were recognized as ferulic acid, oleanolic acid, ursolic acid, tormentic acid, 11-deoxyglycyrrhetic acid, dibenzoyl methane, anisaldehyde, wogonin, protocatechuic acid, psoralen, caffeate, dimethylcaffeic acid, vanillin, aristololactam IIIa, formonentin, *β*-amyrenyl acetate, and 7-methoxy-2-methyl isoflavone. The positive control binding modes can be seen in Figure S1, and the binding patterns between the positive ligands and targets are shown in Figure S2. The detailed molecular docking affinity results of the key targets are shown in Table S5.

### 3.5. Chemical Profiling

Ultraperformance liquid chromatography with quadrupole time-of-flight mass spectrometry (UPLC-QTOF/MS) and gas chromatography-mass spectrometry (GC-MS) was adopted to analyze the volatile and nonvolatile oil compositions of GWK.

**Figure 5:**
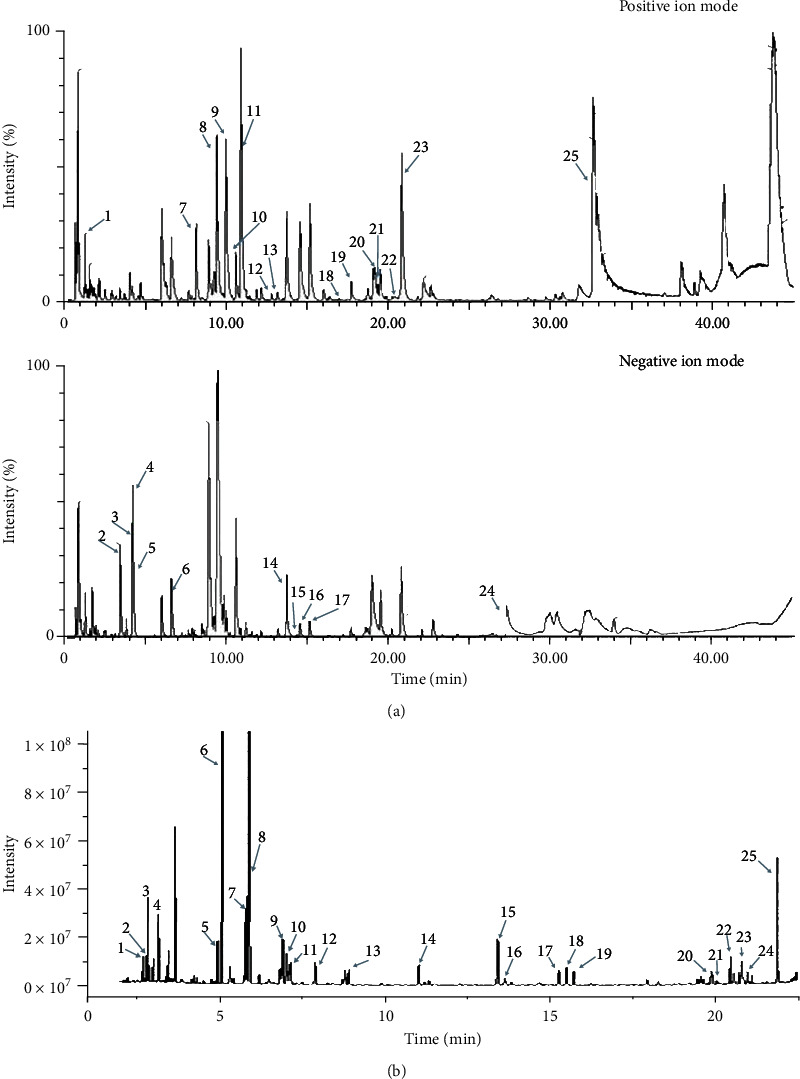
UPLC-QTOF/MS and GC-MS chromatograms of the components in GWK. (a) The UPLC-QTOF/MS chromatogram of the nonvolatile components in GWK in positive ion mode and negative ion mode. (b) The GC-MS chromatogram of the volatile components in GWK.

The UPLC-QTOF/MS chromatograms of nonvolatile components in GWK were identified under positive ion mode and negative ion mode (Figure 5(a)). Based on the detected accurate molecular weight and the major fragments obtained from the MS2 spectrum, 25 components were tentatively identified, including 6 triterpenoid saponins, 12 flavones and their glycosides, 2 phenylpropanoids, 2 organic acids, and 3 other components. Seven ingredients were correspondingly predicted as key active ingredients in the network analysis (Table 4), which further verified the network analysis and molecular docking results. Thus, the key active ingredients of GWK in the treatment of CHB were identified as ferulic acid, oleanolic acid, ursolic acid, tormentic acid, 11-deoxyglycyrrhetic acid, dibenzoyl methane, anisaldehyde, wogonin, protocatechuic acid, psoralen, caffeate, dimethylcaffeic acid, vanillin, beta-amyrenyl acetate, formonentin, aristololactam IIIa, and 7-methoxy-2-methyl isoflavone.

In the GC-MS profile, 25 volatile components of GWK were detected (Figure 5(b)). The mass spectrum of each peak under GC-MS was analyzed using the NIST MS Search 2.2 standard spectral library. Volatile components in GWK were identified as (1) 4-methylheptane, (2) n-octane, (3) 2,4-dimethylheptane, (4) 2,4-dimethyl-1-heptene, (5) 3-thujene, (6) *α*-pinene, (7) sabinene, (8) *β*-pinene, (9) o-cymene, (10) 1,2,6-dimethylnonane, (11) *β*-terpinyl acetate, (12) *γ*-terpinene, (13) (Z)-1,4-dimethylcyclooctane, (14) terpinen-4-ol, (15) 1,3-di-tert-butylbenzene, (16) 4,6-dimethyldodecane, (17) 2-isopropyl-5-methyl-1-heptanol, (18) (5E)-5-icosene, (19) 2-hexyl-1-decanol, (20) 2,4-di-t-butylphenol, (21) 4*β*H,5*α*-eremophila-1(10),11-diene, (22) *γ*-elemene, (23) trichloroacetic acid, (24) atractylon, and (25) 2,2-dimethoxy-2-phenylacetophenone. The detailed component information of UPLC-QTOF/MS and GC-MS is shown in the supplementary file (Tables S6 and S7).

## 4. Discussion

Traditional Chinese medicines (TCMs) usually present the characteristics of multicomponents, multitargets, and comprehensive curative effects, of which the mechanisms are difficult to completely clarify by canonical pharmaceutical research methods. However, network analysis turns out to be an effective approach to investigate the pharmacological mechanism complexities of TCM [51, 52]. The network analysis in this study was assessed based on the “Network Pharmacology Evaluation Methodology Guidance” [23]. Reliability, standardization, and verification were considered in the study in terms of data collection, network analysis, and verification of the results.

In this study, we focused on the molecular mechanism of the exclusive proprietary Chinese medicine Ganweikang (GWK) tablet in the treatment of chronic hepatitis B (CHB). Based on network analysis, the key targets were identified as CA2, NFKB1, RELA, AKT1, JUN, CA1, CA6, IKBKG, FOS, EP300, CREB1, STAT1, MMP9, CDK2, ABCB1, and ABCG2, and the key activate ingredients were ferulic acid, oleanolic acid, ursolic acid, tormentic acid, 11-deoxyglycyrrhetic acid, dibenzoyl methane, anisaldehyde, wogonin, protocatechuic acid, psoralen, caffeate, dimethylcaffeic acid, vanillin, *β*-amyrenyl acetate, formonentin, aristololactam IIIa, and 7-methoxy-2-methyl isoflavone. Detailed information on the key activate ingredients and targets is listed in Tables S8 and S9. Information on key activate ingredients was retrieved from the SwissADME database (http://www.swissadme.ch) [53]. The information on key targets was retrieved from the UniProt database and the GeneCards database (http://www.genecards.org) [54].

Most of the key active ingredients in GWK have been reported to be associated with CHB, HBV, or hepatopathy, mainly correlated with antivirus and liver protection. Ferulic acid, which exists as a phenolic acid in nature, has been reported to have liver protection effects. The antioxidant and anti-inflammatory effects of ferulic acid were revealed to protect acute hepatocyte injury induced by carbon tetrachloride (CCl4) in mice via NOX4/P22PHOx/ROS-JNK/P38 MAPK signaling pathway [55]. Also, ferulic acid was able to attenuate hepatic fibrosis and the activation of hepatic stellate LX-2 cells through the inhibition of TGF-*β*/Smad signaling pathway [56]. The natural triterpenoids oleanolic acid (OA) and ursolic acid (UA) are isomers. Both OA and UA have potential inhibitory effects on the tumorigenic activities mediated by HBx in HBV in vitro and in vivo. HBx protein could induce cell migration by activating SP-1 and Smad3/4 in Huh7 and FL83B cells, simultaneously promoting MMP-3 secretion and inhibiting TGF-*β*-induced apoptosis in Hep3B cells. UA almost blocked all the tumorigenic activities mediated by HBx, while OA presented partial inhibitory effects. The liver protection effects of UA and OA were imposed through the activation of the MAPK signaling pathway in western blotting results [57]. Dibenzoyl methane (DBM), also known as karenzu DK2, is a minor component in Glycyrrhizae Radix et Rhizoma, with the effects of anticancer and antioxidative damage. DBM showed protective effects against CCl4-induced liver injury in a mouse model, which was attributed to the activation of HO-1 expression and the Nrf2 signaling pathway. Moreover, DBM also activated Akt/protein kinase B, mitogen-activated protein kinases, and AMP-activated protein kinases, leading to the increase of intracellular calcium levels. Inhibitions of JNK, AMPK, or intracellular calcium signal could significantly reduce HO-1 expression induced by DBM [58]. Other compounds have also been reported to have liver protection effects, such as tormentic acid [59] and 11-deoxyglycyrrhetic acid [60].

With regard to active antivirus ingredients, the natural flavonoid wogonin has been reported to have anti-inflammation, antitumor, antivirus, neuroprotection, and antianxiety effects. Wogonin displayed inhibiting actions on HBV surface antigen (HBsAg) secretion and directly reduced HBV levels in human HBV-transfected HepG2.2.15 liver cells and mouse model [61]. Involved in various cellular signaling pathways, including PI3K-Akt, p53, NF-*κ*B, and MAPK, the antiviral effects of wogonin have been widely reported and studied. Besides HBV, other viruses, such as the replication of herpes simplex virus types 1 and 2 (HSV-1 and HSV-2), were sensitive to wogonin as well [62]. Protocatechuic acid (PCA) is a phenolic compound that widely exists, amounting to antiviral Chinese herbal medicines. HNF4*α* and HNF1*α* are members of the hepatocyte nuclear factor (HNF) family, which play important roles in the transcription of HBV-related biological targets. Previous studies found that PCA suppressed HBV antigen secretion and HBV DNA replication in HepG2.2.15 cells. Anti-HBV mechanism studies of PCA indicated that PCA inhibited HBV replication by activating the ERK1/2 pathway and subsequently downregulating HNF4*α* and HNF1*α* in HepG2.2.15 cells [63]. Furan coumarin psoralen is derived from the medicinal herb Fangfeng in the GWK prescription. It was reported that psoralen had significant inhibitory effects on HBV E antigen (HEeAg) in HepG2.2.15 cells and downregulated HBV DNA replication [64]. Formononetin is one of the ingredients of herb Huangqi with antiapoptotic and anti-inflammatory effects against multiple liver diseases, such as autoimmune hepatitis (AIH) [65] and nonalcoholic fatty liver disease (NAFLD) [66]. The antitumor capability of formononetin was also revealed in the treatment of hepatocellular carcinoma through its interaction with ubiquitin-specific protease 5 (USP5) [67]. Also, several other GWK active ingredients, such as dimethyl caffeic acid and caffeate, were reported to have antiviral and anti-inflammatory effects as well [68, 69].

Seven of the predicted active ingredients of GWK in the treatment of CHB were also detected in the chemical profiling. The representative docking affinities, such as 3,4-methylenedioxy with CDK2, formononetin with ABCB1, and 7-methoxy-2-methylisoflavone with CA1, were stronger than the positive controls with the same active pockets. The binding modes are demonstrated in Figures 4(g)–4(i) and Figure S1F-S1H, while the binding patterns are shown in Figure S2. The binding modes of wogonin with CREB1 (-3.75 kcal/mol), *β*-amyrenyl acetate with RELA (-7.56 kcal/mol), artemisinin with ABCB1 (-7.26 kcal/mol), and scopoletin with CA6 (-5.81 kcal/mol) could be referred to Figure S5A-D. These active integrates and related targets were mainly involved in antivirus, anti-inflammatory, and antioxidant activities, which indicates the molecular mechanisms of GWK in the treatment of CHB. In addition, some of the ingredients, such as vanillin, had not been reported to be directly related to HBV; nevertheless, molecular docking results showed outperformed affinities compared to positive control compounds, which indicated that these ingredients might have potential therapeutic effects, and further verifications were needed.

These results suggest that GWK may exert its anti-CHB effects through both antiviral protection and liver protection with multitargets CA2, NFKB1, RELA, AKT1, JUN, CA1, CA6, IKBKG, FOS, EP300, CREB1, STAT1, MMP9, CDK2, ABCB1, and ABCG2 and multipathways such as MAPK and NF-*κ*B signaling pathways. Moreover, some of the key targets in our study, such as the AP1 transcription factors JUN and FOS, were associated with the molecular mechanism of HBx protein in HBV infection. HBx protein, acting as a transactivator of HBV X antigen (HBxAg), not only interacts with nuclear transcription factors such as C-Jun, NF-*κ*B, and AP-1 to influence cellular activities at the transcription level but also participates in the regulation of multiple intracytoplasmic signaling pathways like the C-RAF, P53, and JAK-Stat pathways [70, 71]. These results indicate that the scientific basis for the efficacy of GWK in the treatment of CHB might be associated with multiple HBV-related signaling pathways and HBx proteins.

Certainly, several limitations should be noted in this study. First, the protein target prediction tool SEA is based on the chemical similarity of the two-dimensional (2D) structure of compounds (SMILES information), which means that the isometric compounds would be treated the same, and duplicate targets would be predicted. Second, further experimental verifications should be conducted based on the key active ingredients and targets in GWK.

## 5. Conclusion

In conclusion, GWK may exert anti-CHB effects through antiviral and liver protection. The key targets include CA2, NFKB1, RELA, AKT1, JUN, CA1, CA6, IKBKG, FOS, EP300, CREB1, STAT1, MMP9, CDK2, ABCB1, and ABCG2. The related key active ingredients are ferulic acid, oleanolic acid, ursolic acid, tormentic acid, 11-deoxyglycyrrhetic acid, dibenzoyl methane, anisaldehyde, wogonin, protocatechuic acid, psoralen, caffeate, dimethylcaffeic acid, vanillin, *β*-amyrenyl acetate, formonentin, aristololactam IIIa, and 7-methoxy-2-methyl isoflavone. Using computational and experimental methodologies, this study provides a scientific basis for further mechanism elucidation of GWK tablets in the treatment of CHB.

## Figures and Tables

**Table 1:** Network efficacy (NE) of TPT network.

ID	Modularity	NE0	NE	NED
CA2	5	5171.021	5134.971	36.05078
AKT1	2	5171.021	5137.447	33.57426
NFKB1	2	5171.021	5148.723	22.298
RELA	2	5171.021	5148.996	22.02505
JUN	2	5171.021	5150.569	20.45252
IKBKG	2	5171.021	5151.989	19.032
FOS	2	5171.021	5152.703	18.31796
STAT1	2	5171.021	5157.11	13.91142
EP300	2	5171.021	5158.114	12.90776
CREB1	2	5171.021	5158.52	12.50135
CDK2	2	5171.021	5159.81	11.21124
MMP9	2	5171.021	5160.424	10.59769
NLRP3	2	5171.021	5162.762	8.259579
ABCB1	3	5171.021	5162.824	8.197806
ABCG2	3	5171.021	5163.057	7.964864

**Table 2:** Centrality analysis of TPT network.

ID	Modularity	Closeness	Betweenness	Degree	Normalized mean
NFKB1	2	0.658879	668.6669	84	0.664042
AKT1	2	0.646789	699.2082	80	0.649083
RELA	2	0.655814	581.8133	83	0.638257
JUN	2	0.618421	340.2727	71	0.515198
IKBKG	2	0.613043	366.4912	70	0.514695
FOS	2	0.61039	278.2019	68	0.484652
CA2	5	0.382114	1430	16	0.433084
CREB1	2	0.524164	103.653	50	0.329828
GABBR1	1	0.507194	265.5581	37	0.306186
GABBR2	1	0.507194	265.5581	37	0.306186
EP300	2	0.536122	99.59604	43	0.305632
MMP9	2	0.536122	88.26423	43	0.302991
STAT1	2	0.505376	59.06365	43	0.281899
ABCB1	3	0.496479	475.2315	17	0.267778
NLRP3	2	0.482877	15.02849	29	0.203568

**Table 3:** Molecular docking results.

Target	PDB ID	Compound name	PubChem CID	Affinity (kcal/mol)
NFKB1	1SVC	Psoralen	6199	-6.11
		Tectochrysin	Positive control	-6.23
AKT1	6HHG	2-(3,4-Dihydroxyphenyl)-5,7-dihydroxy-6-(3-methylbut-2-enyl) chromone (chromone O)	14604081	-8.8
		GT4	Positive control	-9.01
JUN	5FV8	*cis*-Ferulic acid	1548883	-4.25
		Dimethylcaffeic acid	717531	-4.23
		JNK-IN-8	Positive control	-6.01
CA2	3 DC3	Caffeate	1549111	-5.7
		Protocatechuic acid	72	-5.62
		Karenzu DK2	8433	-6.33
		AZM	Positive control	-5.86
CREB1	5ZKO	Wogonin	5281703	-3.75
		666-15	Positive control	-2.03
EP300	4PZS	*cis*-Ferulic acid	1548883	-5.47
		Vanillin	1183	-6.26
		ACO	Positive control	-4.7
RELA	1NFI	3-Epioleanolic acid	11869658	-6.78
		Tormentic acid	73193	-6.29
		*β*-Amyrenyl acetate	345510	-7.56
		Ursolic acid	49867942	-7.58
		Oleanolic acid	49867939	-7.61
		Dihydroartemisinin	Positive control	-5.19
STAT1	1YVL	Dimethylcaffeic acid	717531	-5.58
		Daidzein dimethyl ether	136419	-4.56
		Glazarin	746449	-5.42
		Fludarabine	Positive control	-2.21
CDK2	2IW8	3,4-Methylenedioxy-10-hydroxy aristololactam	5319620	-7.99
		4SP	Positive control	-7.58
ABCB1	6UJN	Formononetin	10378473	-7.19
		Daidzein dimethyl ether	136419	-7.26
		Tepotinib	Positive control	-6.53
CA1	1AZM	7-Methoxy-2-methylisoflavone	354368	-7.18
		AZM	Positive control	-5.7
CA6	3FE4	Scopoletin	5280460	-5.81
		Sinapic acid	54710960	-5.63
		Zonisamide	Positive control	-8

**Table 4:** Active ingredients in network prediction and UPLC-QTOF/MS analysis.

Serial no.	Peak no.	Retention time (min)	Negative ion mode	Positive ion mode	Name	Compound type	Herb source	Biological activity	Molecular formula
1	1	1.35	191.1568[M-H]-, 111.0086	193.0256[M+H]+	Scopoletin	Coumarin	Baizhu, Gancao	Anti-inflammatory, antitumoral, antioxidative and hepatoprotective activities	C_10_H_8_O_4_
2	6	6.62	433.1342[M+HCOO-]-, 161.0251	411.1229[M+NA]+, 177.0501	Artemisinin	Sesquiterpene lactone	Mabiancao	Antitumor and antivirus activity	C_20_H_20_O_8_
3	7	8.07	469.1690[M-H]-,163.0341	—	*β*-Amyrenyl acetate	Terpenoids	Lianqiao	Antioxidant and cytotoxic agents	C_32_H_52_O_2_
4	15	14.60	283.0593[M-H]-, 211.0378	285.0722[M+H]+ , 213.0504	Wogonin	Flavonoid	Yinchaihu	Anti-inflammatory agent	C_16_H_12_O_5_
5	20	19.16	267.0641	269.0767	Formonentin	Flavonoid	Huangqi	Antioxidant, anticancer, and anti-inflammatory activities	C_16_H_12_O_4_
6	24	27.38	265.1461, 152.9934	—	7-Methoxy-2-methylisoflavone	Flavonoid	Gancao	—	C_17_H_14_O_3_
7	25	32.44	—	301.1379[M+NA]+, 149.0186	Aristololactam IIIa	Lactam	Huoxiang	CDK2 inhibition	C_16_H_9_NO_4_

## Data Availability

Transcriptome microarray data is available on GitHub community (https://github.com). Data link: https://github.com/xujiaqi0/Ganweikang-data-availability.git.

## Abbreviations

GWK: Geiweikang
TCM: Traditional Chinese medicine
TPT: Target-pathway-target
NE: Network efficacy
UPLC-QTOF-MS: Ultraperformance liquid chromatography coupled with a hybrid quadrupole orthogonal time-of-flight mass spectrometer
GC-MS: Gas chromatography-mass spectrometry.

## Supplementary Materials

Supplementary Materials Figure S1: schematic diagrams for the binding modes between targets and positive control small molecules. Figure S2: the binding patterns between active ingredients, positive control, and targets. Table S1: DEG results and disease-related targets. Table S2: compound-related targets. Table S3: KEGG pathway enrichment results on each module in TPT network. Table S4: detail information of CTP network. Table S5: molecular docking results of key targets. Table S6: detail information of UPLC-QTOF/MS analysis. Table S7: detail information of GC/MS analysis. Table S8: detail information of key active ingredients. Table S9: detail information of key targets.

## References

[1] Paccoud O., Surgers L., Lacombe K. (2019). Hepatitis B virus infection: natural history, clinical manifestations and therapeutic approach. *La Revue de Médecine Interne*.

[2] WHO Fact sheets: hepatitis B 2021. https://www.who.int/news-room/fact-sheets/detail/hepatitis-b.

[3] Di Bisceglie A. M. (2009). Hepatitis B and hepatocellular carcinoma. *Hepatology*.

[4] Zeng Y., Wu W., Fu Y. (2019). Toll-like receptors, long non-coding RNA NEAT1, and RIG-I expression are associated with HBeAg-positive chronic hepatitis B patients in the active phase. *Journal of Clinical Laboratory Analysis*.

[5] Honda M., Yamashita T., Ueda T., Takatori H., Nishino R., Kaneko S. (2006). Different signaling pathways in the livers of patients with chronic hepatitis B or chronic hepatitis C. *Hepatology*.

[6] Yu X., Lan P., Hou X. (2017). HBV inhibits LPS-induced NLRP3 inflammasome activation and IL-1*β* production via suppressing the NF-*κ*B pathway and ROS production. *Journal of Hepatology*.

[7] Guidotti L. G. (2002). The role of cytotoxic T cells and cytokines in the control of hepatitis B virus infection. *Vaccine*.

[8] Bertoletti A., Kennedy P. T. (2015). The immune tolerant phase of chronic HBV infection: new perspectives on an old concept. *Cellular & Molecular Immunology*.

[9] Suhail M., Abdel-Hafiz H., Ali A. (2014). Potential mechanisms of hepatitis B virus induced liver injury. *World Journal of Gastroenterology*.

[10] Campos-Valdez M., Monroy-Ramírez H. C., Armendáriz-Borunda J., Sánchez-Orozco L. V. (2021). Molecular mechanisms during hepatitis B infection and the effects of the virus variability. *Viruses*.

[11] Yu R., Fan R., Hou J. (2014). Chronic hepatitis B virus infection: epidemiology, prevention, and treatment in China. *Frontiers in Medicine*.

[12] Wang T., Jin W., Huang Q. (2020). Clinical efficacy and safety of eight traditional Chinese medicine combined with entecavir in the treatment of chronic hepatitis B liver fibrosis in adults: a network meta-analysis. *Evidence-based Complementary and Alternative Medicine*.

[13] Chen K.-X. (2020). Academician Kai-Xian Chen talks about the development of traditional Chinese medicine and global medicine. *World Journal of Traditional Chinese Medicine*.

[14] Zheng X., Wang J., Yang D. (2015). Antiviral therapy for chronic hepatitis B in China. *Medical Microbiology and Immunology*.

[15] Gu P., Chen H. (2014). Modern bioinformatics meets traditional Chinese medicine. *Briefings in Bioinformatics*.

[16] WY-y L. I. S., Liang J. I., Yan-da L. I. (2002). A discussion and case study of complexities in traditional Chinese medicine. *Journal of System SIMULATION*.

[17] Li S., Zhang Z. Q., Wu L. J., Zhang X. G., Li Y. D., Wang Y. Y. (2007). Understanding ZHENG in traditional Chinese medicine in the context of neuro-endocrine-immune network. *IET Systems Biology*.

[18] Hopkins A. L. (2008). Network pharmacology: the next paradigm in drug discovery. *Nature Chemical Biology*.

[19] Hopkins A. L. (2007). Network pharmacology. *Nature Biotechnology*.

[20] Li S., Zhang B., Zhang N. (2011). Network target for screening synergistic drug combinations with application to traditional Chinese medicine. *BMC Systems Biology*.

[21] Li S., Zhang B. (2013). Traditional Chinese medicine network pharmacology: theory, methodology and application. *Chinese Journal of Natural Medicines*.

[22] Wang Z. Y., Wang X., Zhang D. Y., Hu Y. J., Li S. (2022). Traditional Chinese medicine network pharmacology: development in new era under guidance of network pharmacology evaluation method guidance. *China Journal of Chinese Material Médica*.

[23] Societies WFoCM (2021). Network pharmacology evaluation methodology guidance. *World Chinese Medicine*.

[24] Tang L. L., Sheng J. F., Xu C. H., Liu K. Z. (2009). Clinical and experimental effectiveness of Astragali compound in the treatment of chronic viral hepatitis B. *The Journal of International Medical Research*.

[25] Bilia A. R., Giomi M., Innocenti M., Gallori S., Vincieri F. F. (2008). HPLC-DAD-ESI-MS analysis of the constituents of aqueous preparations of verbena and lemon verbena and evaluation of the antioxidant activity. *Journal of Pharmaceutical and Biomedical Analysis*.

[26] Shi X.-Y., Miao Q.-Y., Liu X.-G., Li P., Gao W. (2021). Screening safflower injection for constituents with activity against stroke using comprehensive chemical profiling coupled with network pharmacology. *World Journal of Traditional Chinese Medicine*.

[27] Zhao X.-S., Zeng Y.-X., Zhou Y.-K., Li R.-T., Yang M.-H. (2021). Gas chromatography-mass spectrometry for quantitative and qualitative analysis of essential oil from Curcuma wenyujin rhizomes. *World Journal of Traditional Chinese Medicine*.

[28] Ru J., Li P., Wang J. (2014). TCMSP: a database of systems pharmacology for drug discovery from herbal medicines. *Journal of Cheminformatics*.

[29] Xue R., Fang Z., Zhang M., Yi Z., Wen C., Shi T. (2013). TCMID: traditional Chinese medicine integrative database for herb molecular mechanism analysis. *Nucleic Acids Research*.

[30] Shanghai Institute of Organic Chemistry of CAS Chemistry Database [DB/OL] cited 1978-2021. http://www.organchem.csdb.cn.

[31] Wang Y., Xiao J., Suzek TO (2012). PubChem’s BioAssay database. *Nucleic Acids Research*.

[32] Cheng F., Li W., Zhou Y. (2012). admetSAR: a comprehensive source and free tool for assessment of chemical ADMET properties. *Journal of Chemical Information and Modeling*.

[33] Keiser M. J., Roth B. L., Armbruster B. N., Ernsberger P., Irwin J. J., Shoichet B. K. (2007). Relating protein pharmacology by ligand chemistry. *Nature Biotechnology*.

[34] Davis A. P., Grondin C. J., Johnson R. J. (2021). Comparative toxicogenomics database (CTD): update 2021. *Nucleic Acids Research*.

[35] Amberger J. S., Bocchini C. A., Schiettecatte F., Scott A. F., Hamosh A. (2015). OMIM.org: Online Mendelian Inheritance in Man (OMIM®), an online catalog of human genes and genetic disorders. *Nucleic Acids Research*.

[36] Kanehisa M., Goto S. (2000). KEGG: Kyoto Encyclopedia of Genes and Genomes. *Nucleic Acids Research*.

[37] Uni Prot (2021). The universal protein knowledgebase in 2021. *Nucleic Acids Research*.

[38] Lu Y., Fang Z., Li M. (2019). Dynamic edge-based biomarker non-invasively predicts hepatocellular carcinoma with hepatitis B virus infection for individual patients based on blood testing. *Journal of Molecular Cell Biology*.

[39] Team R. C. A language and environment for statistical computing 2019. https://www.R-project.org/.

[40] Li S. (2021). *Network Pharmacology*.

[41] Zuo H., Zhang Q., Su S., Chen Q., Yang F., Hu Y. (2018). A network pharmacology-based approach to analyse potential targets of traditional herbal formulas: an example of Yu Ping Feng decoction. *Scientific Reports*.

[42] Batagelj V. M. A., Mrvar A., Jünger M. (2004). Pajek — analysis and visualization of large networks. *Graph Drawing Software Mathematics and Visualization*.

[43] Bastian M., Heymann S., Jacomy M. (2009). Gephi: an open source software for exploring and manipulating networks. *Proceedings of the International AAAI Conference on Web and Social Media*.

[44] Zhang D., Zhang Y., Gao Y. (2020). Translating traditional herbal formulas into modern drugs: a network-based analysis of Xiaoyao decoction. *Chinese Medicine*.

[45] Shannon P., Markiel A., Ozier O. (2003). Cytoscape: a software environment for integrated models of biomolecular interaction networks. *Genome Research*.

[46] Ormö M., Cubitt A. B., Kallio K., Gross L. A., Tsien R. Y., Remington S. J. (1996). Crystal structure of the Aequorea victoria green fluorescent protein. *Science*.

[47] Schrödinger L., DeLano W. PyMOL 2020. http://www.pymol.org/pymol.

[48] Forli S., Huey R., Pique M. E., Sanner M. F., Goodsell D. S., Olson A. J. (2016). Computational protein-ligand docking and virtual drug screening with the AutoDock suite. *Nature Protocols*.

[49] Yu J., Zhou Y., Tanaka I., Yao M. (2010). Roll: a new algorithm for the detection of protein pockets and cavities with a rolling probe sphere. *Bioinformatics*.

[50] O'Boyle N. M., Banck M., James C. A., Morley C., Vandermeersch T., Hutchison G. R. (2011). Open Babel: an open chemical toolbox. *Journal of Cheminformatics*.

[51] Ma Y. M., Zhang X. Z., Su Z. Z. (2015). Insight into the molecular mechanism of a herbal injection by integrating network pharmacology and in vitro. *Journal of Ethnopharmacology*.

[52] Sheng S., Yang Q.-N., Zhu H.-N., Xian Y.-Y. (2021). Network pharmacology-based exploration of the mechanism of guanxinning tablet for the treatment of stable coronary artery disease. *World Journal of Traditional Chinese Medicine*.

[53] Daina A., Michielin O., Zoete V. (2017). SwissADME: a free web tool to evaluate pharmacokinetics, drug-likeness and medicinal chemistry friendliness of small molecules. *Scientific Reports*.

[54] Stelzer G., Rosen N., Plaschkes I. (2016). The GeneCards suite: from gene data mining to disease genome sequence analyses. *Current Protocols in Bioinformatics*.

[55] Luna-Vital D., Luzardo-Ocampo I., Cuellar-Nuñez M. L., Loarca-Piña G., Gonzalez de Mejia E. (2020). Maize extract rich in ferulic acid and anthocyanins prevents high-fat-induced obesity in mice by modulating SIRT1, AMPK and IL-6 associated metabolic and inflammatory pathways. *The Journal of Nutritional Biochemistry*.

[56] Mu M., Zuo S., Wu R. M. (2019). Ferulic acid attenuates liver fibrosis and hepatic stellate cell activation via inhibition of TGF-*β*/Smad signaling pathway [corrigendum]. *Drug Design, Development and Therapy*.

[57] Jesus J. A., Lago J. H., Laurenti M. D., Yamamoto E. S., Passero L. F. (2015). Antimicrobial activity of oleanolic and ursolic acids: an update. *Evidence-based Complementary and Alternative Medicine*.

[58] Cao M., Wang H., Guo L., Yang S., Liu C., Khor TO (2017). Dibenzoylmethane protects against CCl4-induced acute liver injury by activating Nrf 2 via JNK, AMPK, and calcium signaling. *The AAPS Journal*.

[59] Jiang W. P., Huang S. S., Matsuda Y. (2017). Protective effects of tormentic acid, a major component of suspension cultures of Eriobotrya japonica cells, on acetaminophen-induced hepatotoxicity in mice. *Molecules*.

[60] Latief U., Ahmad R. (2018). Herbal remedies for liver fibrosis: a review on the mode of action of fifty herbs. *Journal of Traditional and Complementary Medicine*.

[61] Guo Q., Zhao L., You Q. (2007). Anti-hepatitis B virus activity of wogonin in vitro and in vivo. *Antiviral Research*.

[62] Chu Y., Lv X., Zhang L. (2020). Wogonin inhibits in vitro herpes simplex virus type 1 and 2 infection by modulating cellular NF-*κ*B and MAPK pathways. *BMC Microbiology*.

[63] Dai X. Q., Cai W. T., Wu X., Chen Y., Han F. M. (2017). Protocatechuic acid inhibits hepatitis B virus replication by activating ERK1/2 pathway and down-regulating HNF4*α* and HNF1*α* in vitro. *Life Sciences*.

[64] Parvez M. K., Tabish Rehman M., Alam P., Al-Dosari M. S., Alqasoumi S. I., Alajmi M. F. (2019). Plant-derived antiviral drugs as novel hepatitis B virus inhibitors: cell culture and molecular docking study. *Saudi Pharmaceutical Journal*.

[65] Liu G., Zhao W., Bai J., Cui J., Liang H., Lu B. (2021). Formononetin protects against concanavalin-A-induced autoimmune hepatitis in mice through its anti-apoptotic and anti-inflammatory properties. *Biochemistry and Cell Biology*.

[66] Wang Y., Zhao H., Li X. (2019). Formononetin alleviates hepatic steatosis by facilitating TFEB-mediated lysosome biogenesis and lipophagy. *The Journal of Nutritional Biochemistry*.

[67] Meng J., Ai X., Lei Y. (2019). USP5 promotes epithelial-mesenchymal transition by stabilizing SLUG in hepatocellular carcinoma. *Theranostics*.

[68] Khan F., Bamunuarachchi N. I., Tabassum N., Kim Y. M. (2021). Caffeic acid and its derivatives: antimicrobial drugs toward microbial pathogens. *Journal of Agricultural and Food Chemistry*.

[69] Wang G. F., Shi L. P., Ren Y. D. (2009). Anti-hepatitis B virus activity of chlorogenic acid, quinic acid and caffeic acid in vivo and in vitro. *Antiviral Research*.

[70] Natoli G., Avantaggiati M. L., Chirillo P. (1994). Induction of the DNA-binding activity of c-jun/c-fos heterodimers by the hepatitis B virus transactivator pX. *Molecular and Cellular Biology*.

[71] Wu Y. H., Ai X., Liu F. Y., Liang H. F., Zhang B. X., Chen X. P. (2016). c-Jun N-terminal kinase inhibitor favors transforming growth factor-*β* to antagonize hepatitis B virus X protein-induced cell growth promotion in hepatocellular carcinoma. *Molecular Medicine Reports*.

