# Supplementary material for: Mechanisms of Ganweikang Tablets against Chronic Hepatitis B: A Comprehensive Study of Network Analysis, Molecular Docking, and Chemical Profiling
Source: Biomed Res Int. 2023 May 8;2023:8782892. doi: 10.1155/2023/8782892 (PMC10185428; doi:10.1155/2023/8782892)
Supplement: Supplementary Materials — Figure S1: schematic diagrams for the binding modes between targets and positive control small molecules. Figure S2: the binding patterns between active ingredients, positive control, and targets. Table S1: DEG results and disease-related targets. Table S2: compound-related targets. Table S3: KEGG pathway enrichment results on each module in TPT network. Table S4: detail information of CTP network. Table S5: molecular docking results of key targets. Table S6: detail information of UPLC-QTOF/MS analysis. Table S7: detail information of GC/MS analysis. Table S8: detail information of key active ingredients. Table S9: detail information of key targets. [file 8782892.f1.zip › Supplementary figures (2).pdf]

Mechanisms of Ganweikang Tablets against Chronic Hepatitis B: A Comprehensive Study of Network Analysis, Molecular Docking and Chemical Profiling

Jia-Qi Xu[1,2], Shi-Bing Su[3], CY Chen[4,6], J Gao[6,7], ZM Cao[4,6], JL Guan[5,6], Lin-Xuan Xiao[1], Mingming Zhao[1], Hua Yu[1*], Yuan-Jia Hu[1,2*]

Supplementary figures:

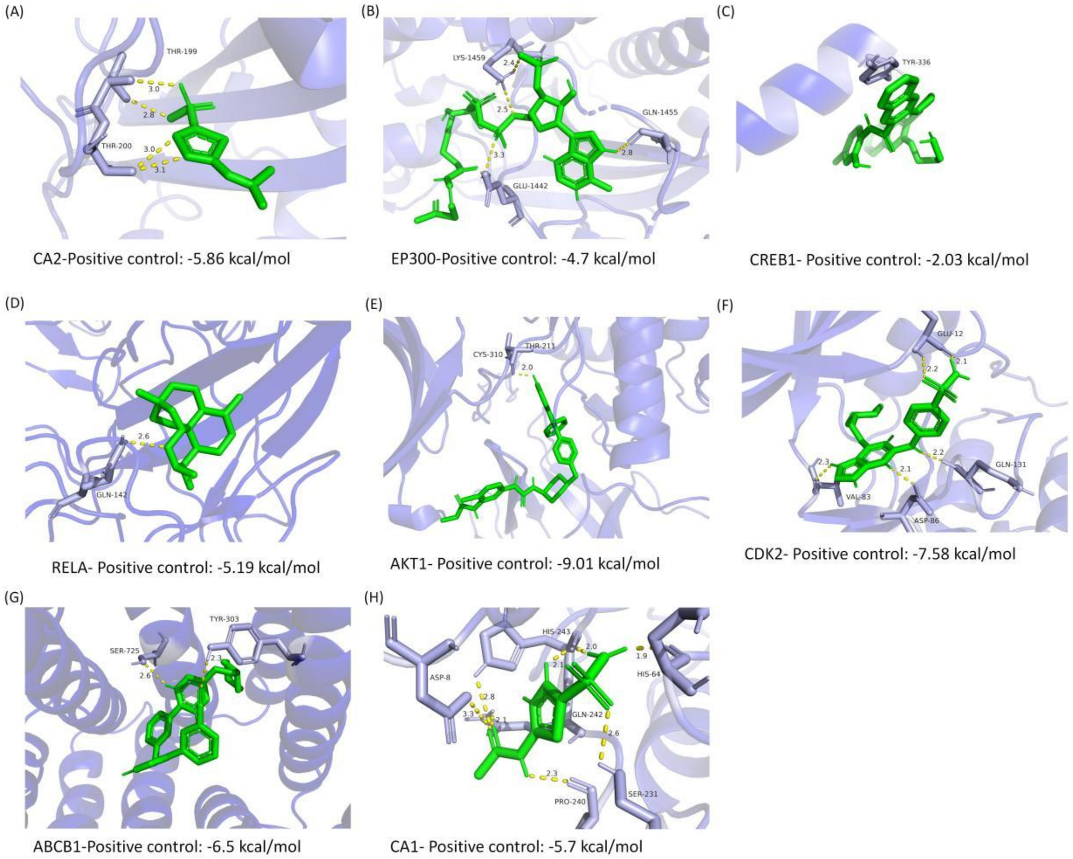

Figure S1 Schematic diagrams for the binding modes between targets and positive control small molecules

(A) CA2 with positive control; (B) EP300 with positive control; (C) CREB1 with positive control; (D) RELA with positive control; (E) AKT1 with positive control; (F) CDK2 with positive control; (G) ABCB1 with positive control; (H) CA1 with positive control

(A)

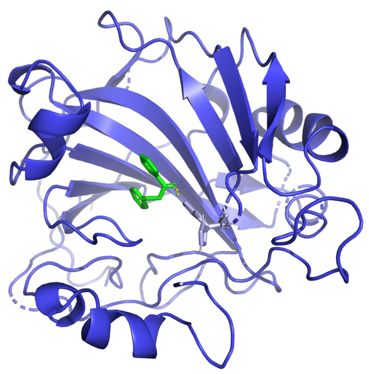

CA2- DBM : -6.33 kcal/mol

(B)

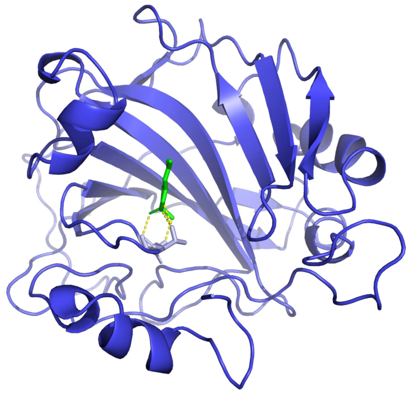

CA2- Positive control: -5.86 kcal/mol

(C)

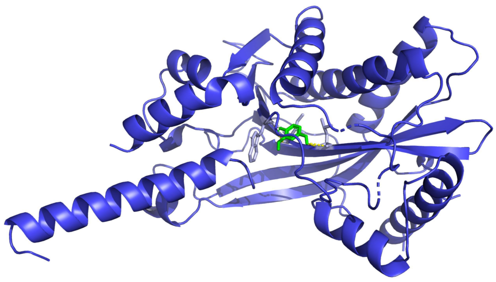

EP300-vanillin: -6.26 kcal/mol

(D)

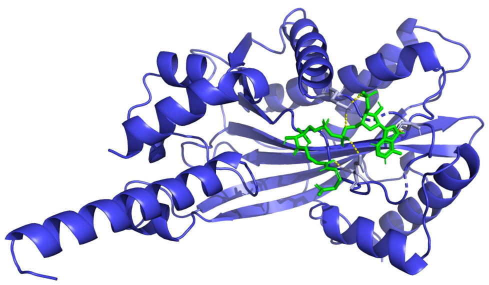

EP300-Positive control: -4.7 kcal/mol

(E)

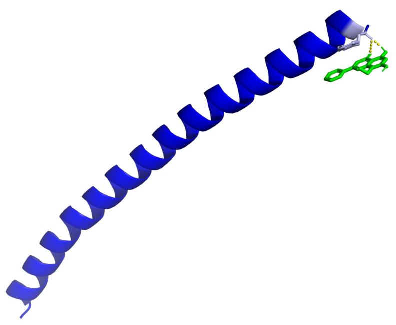

CREB1- wogonin: -3.67 kcal/mol

(F)

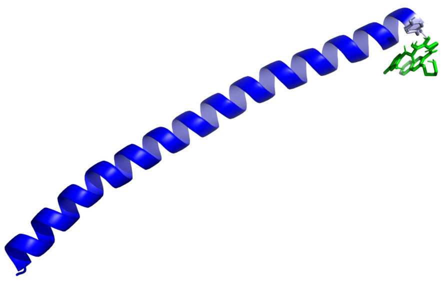

CREB1-Positive control: -2.03 kcal/mol

(G)

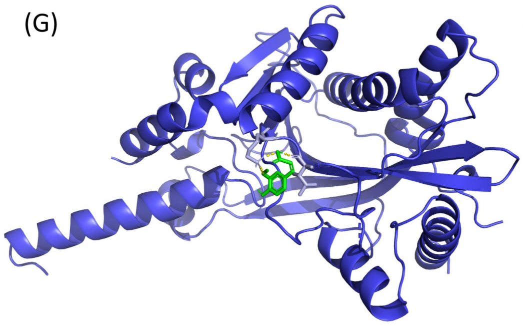

EP300-cis-ferulic acid: - 5.47kcal/mol

(H)

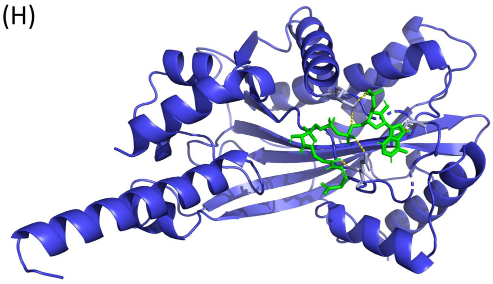

EP300-Positive control: - 4.7kcal/mol

(I)

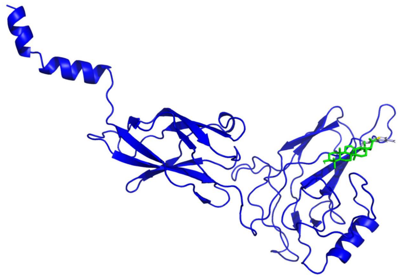

RELA- oleanolic acid: -6.87 kcal/mol

(J)

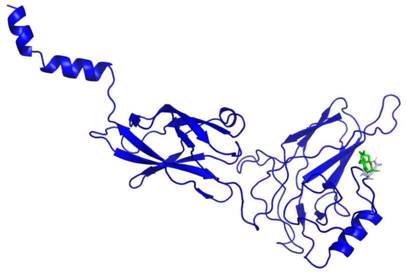

RELA-Positive control: -5.19 kcal/mol

(K)

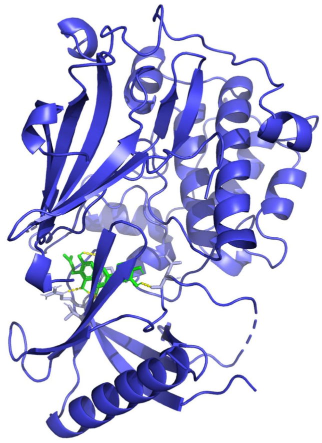

AKT1- chromone O: -8.8 kcal/mol

(L)

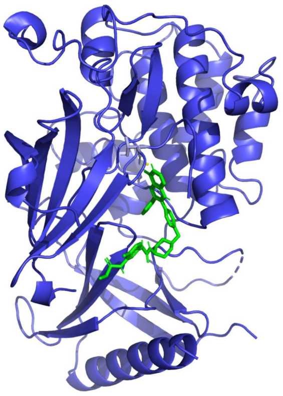

AKT-1-Positive control: -9.01 kcal/mol

(M)

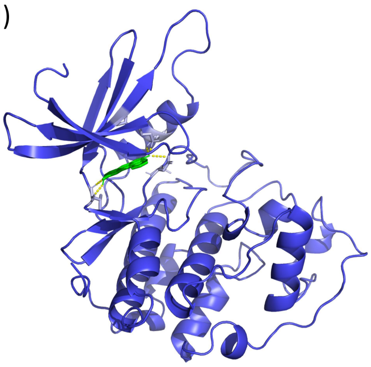

CDK2- 3,4-methylenedioxy: -7.99 kcal/mol

(N)

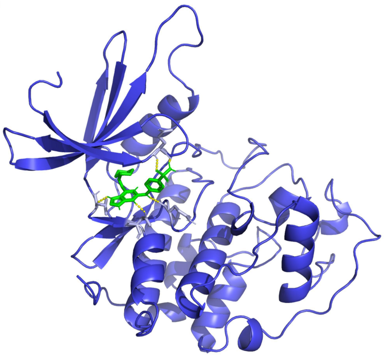

CDK2-Positive control: -7.58 kcal/mol

(O)

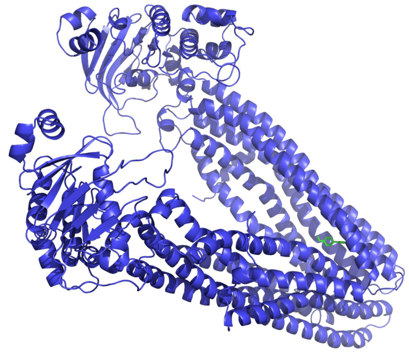

ABCB1-formononetin: -7.19 kcal/mol

(P)

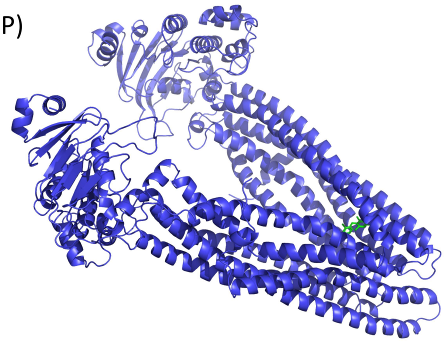

ABCB1-Positive control: -6.27 kcal/mol

(Q)

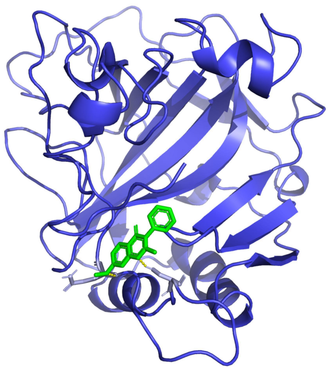

CA1- 7-methoxy-2 methylisoflavone: -7.18 kcal/mol

(R)

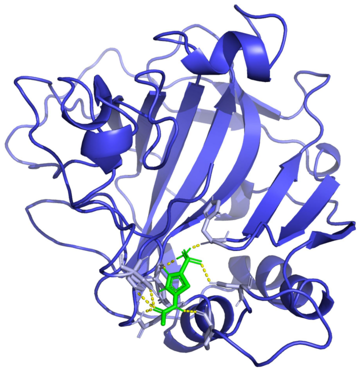

CA1-Positive control: -5.7 kcal/mol

FigureS2 The binding patterns between active ingredients, positive control and targets

(A) CA2 with Karenzu DK2; (B) CA2 with positive control; (C) EP300 with vanillin; (D) EP300 with positive control; (E) CREB1 with wogonin; (F) CREB1 with positive control; (G) EP300 with cis-ferulic acid; (H) EP300 with positive control; (I) RELA with oleanolic acid; (J) RELA with positive control; (K) AKT1 with chromone O; (L) AKT1 with positive control; (M) CDK2 with 3,4-methylendioxy; (N) CDK2 with positive control; (O) ABCB1 with formononetin; (P) ABCB1 with positive control; (Q) CA1 with 7-methoxy-2-methylisoflavone; (R) CA1 with positive control
