# Supplementary material for: Mechanisms of Ganweikang Tablets against Chronic Hepatitis B: A Comprehensive Study of Network Analysis, Molecular Docking, and Chemical Profiling
Source: Biomed Res Int. 2023 May 8;2023:8782892. doi: 10.1155/2023/8782892 (PMC10185428; doi:10.1155/2023/8782892)
Supplement: Supplementary Materials — Figure S1: schematic diagrams for the binding modes between targets and positive control small molecules. Figure S2: the binding patterns between active ingredients, positive control, and targets. Table S1: DEG results and disease-related targets. Table S2: compound-related targets. Table S3: KEGG pathway enrichment results on each module in TPT network. Table S4: detail information of CTP network. Table S5: molecular docking results of key targets. Table S6: detail information of UPLC-QTOF/MS analysis. Table S7: detail information of GC/MS analysis. Table S8: detail information of key active ingredients. Table S9: detail information of key targets. [file 8782892.f1.zip › Supplementary table S1.docx]

| Gene name | logFC | AveExpr | P.Value | DEG |
| --- | --- | --- | --- | --- |
| ASGR1 | 4.258233586 | 5.050539584 | 1.05E-09 | up |
| TMEM144 | 4.398109453 | 5.190125684 | 3.77E-09 | up |
| MS4A4A | 4.543682976 | 6.030578663 | 9.17E-07 | up |
| BRI3 | 0.716653233 | 10.77943742 | 2.03E-06 | up |
| ABCA1 | 1.214438456 | 11.12666992 | 3.95E-06 | up |
| ETV7 | 5.20010912 | 5.514817463 | 4.46E-06 | up |
| SMAD1 | 3.158032653 | 4.843153584 | 6.85E-06 | up |
| MTMR11 | 0.824797072 | 10.033197 | 7.25E-06 | up |
| CTCFL | 2.825874689 | 6.485409621 | 9.04E-06 | up |
| LGALS9B | 3.252989197 | 4.815941089 | 1.05E-05 | up |
| CYTSB | 0.979006672 | 9.829571553 | 1.18E-05 | up |
| GAPT | 0.639821283 | 10.66458079 | 1.28E-05 | up |
| HHEX | 0.628735933 | 11.36211818 | 1.35E-05 | up |
| SCO2 | 2.253456692 | 9.313181384 | 1.40E-05 | up |
| C13orf31 | 3.623630048 | 5.545689868 | 1.63E-05 | up |
| C14orf143 | 4.398867077 | 5.259402168 | 1.90E-05 | up |
| SLC27A3 | 3.600779441 | 5.562941537 | 2.53E-05 | up |
| MOV10 | 3.84606459 | 5.765073479 | 3.27E-05 | up |
| OSCAR | 0.827585656 | 10.38521416 | 4.62E-05 | up |
| DRAM1 | 1.147177508 | 8.689824142 | 4.68E-05 | up |
| PLXNB2 | 2.366901106 | 3.984448632 | 5.09E-05 | up |
| CCRL2 | 2.844107603 | 6.930997895 | 5.19E-05 | up |
| IFI27 | 7.523750166 | 7.369576247 | 5.25E-05 | up |
| MYOF | 3.832270994 | 4.513207711 | 5.93E-05 | up |
| SASH1 | 4.243278331 | 6.376393826 | 6.70E-05 | up |
| ITSN1 | 2.907036902 | 4.9278137 | 7.18E-05 | up |
| RHCE | 3.939444677 | 4.615956032 | 8.40E-05 | up |
| ALDH3B1 | 0.755849411 | 10.01048763 | 8.63E-05 | up |
| TFEC | 1.611935406 | 9.9620265 | 9.36E-05 | up |
| DNMT1 | 0.592777639 | 10.45849466 | 0.000102396 | up |
| GBA | 0.820059644 | 9.071220605 | 0.000102577 | up |
| TNS3 | 1.02871084 | 8.020754742 | 0.000115047 | up |
| VRK2 | 1.117662278 | 9.478778474 | 0.00012621 | up |
| CDC42EP4 | 0.791892356 | 9.790184105 | 0.000134575 | up |
| NFIL3 | 0.808400844 | 10.18601832 | 0.000140694 | up |
| CD1D | 0.668292456 | 10.29158668 | 0.000146636 | up |
| PTMS | 2.825046353 | 5.180628332 | 0.000177741 | up |
| EXT1 | 2.78806987 | 5.197978405 | 0.000179554 | up |
| SLC6A12 | 0.719181766 | 1.671070137 | 0.000211447 | up |
| PRKCD | 0.735852922 | 9.969271211 | 0.000261001 | up |
| HNMT | 2.120475617 | 6.781472568 | 0.000270829 | up |
| SCLT1 | 0.966848928 | 9.052068921 | 0.000281044 | up |
| RUNDC3A | 2.888167708 | 4.796627511 | 0.000304492 | up |
| CPNE8 | 2.443841774 | 7.152869932 | 0.000331279 | up |
| DHRS9 | 1.180701856 | 10.17464353 | 0.000331815 | up |
| NSUN3 | 0.963446917 | 9.265293342 | 0.000371761 | up |
| HMOX1 | 2.924613444 | 5.683676711 | 0.000411845 | up |
| MCOLN1 | 3.319347166 | 5.242297632 | 0.000412654 | up |
| ZNF124 | 1.039401741 | 9.059575563 | 0.00041335 | up |
| TRIM21 | 0.645075472 | 11.48033882 | 0.000425947 | up |
| SLC2A6 | 2.693785673 | 5.9280478 | 0.000451831 | up |
| SESTD1 | 1.60272199 | 8.215899611 | 0.000460482 | up |
| ERMAP | 0.930328498 | 8.4127722 | 0.000461704 | up |
| TCF7L2 | 0.709201494 | 11.333901 | 0.000480297 | up |
| UCHL3 | 0.748457422 | 9.132472263 | 0.000487269 | up |
| FANCL | 0.942730833 | 9.645978211 | 0.000493159 | up |
| NHSL1 | 2.007561967 | 4.065717705 | 0.000494806 | up |
| NLRP3 | 2.646602693 | 6.801456547 | 0.000496101 | up |
| DHX58 | 3.838524917 | 6.903252795 | 0.000552526 | up |
| LGALS9 | 1.333698361 | 8.962757605 | 0.000566093 | up |
| ANKRD32 | 0.86025625 | 10.61853255 | 0.000586395 | up |
| CDKN1A | 0.884924683 | 9.6538795 | 0.000589731 | up |
| ARHGEF11 | 0.783869673 | 8.516226347 | 0.000603206 | up |
| F5 | 1.095418732 | 8.964547047 | 0.000618165 | up |
| NOD2 | 0.716569511 | 10.67872626 | 0.000631327 | up |
| FHDC1 | 2.999824246 | 4.224488689 | 0.000670432 | up |
| C19orf38 | 0.654068878 | 9.601625 | 0.000685191 | up |
| MAFB | 1.156584278 | 9.960529711 | 0.000714552 | up |
| PRIC285 | 3.295145561 | 5.882690974 | 0.000717744 | up |
| SAMD4A | 3.50001912 | 7.430527174 | 0.000725929 | up |
| LOC100288142 | 3.013822294 | 5.779107084 | 0.000770154 | up |
| TMCC2 | 1.595626266 | 9.515414758 | 0.000800813 | up |
| SORT1 | 0.853846739 | 9.851441868 | 0.000819267 | up |
| UBXN11 | 2.786569006 | 5.912039889 | 0.000835143 | up |
| C19orf77 | 2.453681303 | 8.300695121 | 0.000848596 | up |
| GRN | 0.774433211 | 10.21492068 | 0.000854951 | up |
| LYL1 | 2.802260788 | 4.266757279 | 0.000858147 | up |
| SCPEP1 | 0.705559333 | 10.03481532 | 0.000861568 | up |
| CACNA1A | 3.389399308 | 5.857952405 | 0.000881794 | up |
| PARP9 | 0.772367644 | 12.50685116 | 0.000897735 | up |
| SNORA33 | 2.622193708 | 6.250398126 | 0.000918698 | up |
| C18orf49 | 2.91887269 | 8.082967716 | 0.000928759 | up |
| APOBEC3A | 1.167043211 | 10.98432084 | 0.000956387 | up |
| NAGK | 0.59142435 | 11.63436003 | 0.00104876 | up |
| EPN1 | 0.709714012 | 8.720101532 | 0.001051508 | up |
| SIGLEC1 | 5.606141671 | 7.737205732 | 0.00110161 | up |
| ITIH4 | 2.928804591 | 5.994018911 | 0.001142957 | up |
| KCNK6 | 2.19597335 | 4.720595853 | 0.001150409 | up |
| LRRC25 | 0.625538478 | 9.676361 | 0.001153594 | up |
| SLC46A2 | 2.935633068 | 5.362803942 | 0.00116199 | up |
| DDX60L | 0.821354244 | 12.36053474 | 0.001163566 | up |
| SLC22A4 | 0.75531105 | 10.98856887 | 0.001219792 | up |
| TBC1D2 | 2.266861866 | 4.336240763 | 0.001225203 | up |
| STX11 | 0.660696578 | 11.28676374 | 0.001227165 | up |
| RBBP8 | 2.287961253 | 5.9620396 | 0.001248677 | up |
| JAK2 | 0.782100078 | 10.86963416 | 0.001291445 | up |
| LGALS9 | 1.131614011 | 9.715299789 | 0.001292632 | up |
| ODF3B | 1.733362366 | 8.470151074 | 0.001326094 | up |
| C14orf145 | 2.548223516 | 5.539732384 | 0.001349093 | up |
| LMO2 | 0.678485261 | 11.65089961 | 0.001361124 | up |
| CYSLTR1 | 1.683832864 | 8.719888621 | 0.001363371 | up |
| C11orf75 | 1.971878277 | 3.460332895 | 0.001384804 | up |
| GADD45A | 0.8022787 | 9.699354053 | 0.001436603 | up |
| ZBP1 | 3.10752432 | 7.299224584 | 0.001463886 | up |
| RASSF4 | 0.716522578 | 9.593984132 | 0.001466656 | up |
| LHFPL2 | 0.781630589 | 10.43387487 | 0.001476545 | up |
| ATP8B4 | 2.32830886 | 7.474380784 | 0.001499624 | up |
| IRF7 | 1.438425789 | 10.10438868 | 0.001500697 | up |
| AHSP | 3.39281647 | 7.181891321 | 0.001521669 | up |
| IFI44L | 2.909507522 | 11.21360847 | 0.001585623 | up |
| IFIT1B | 3.98295964 | 6.364881453 | 0.001695602 | up |
| SLC43A3 | 1.017473834 | 8.0792882 | 0.001706276 | up |
| FAM129B | 1.057831894 | 2.375092616 | 0.001714509 | up |
| OPRL1 | 2.310825919 | 5.608799821 | 0.001731571 | up |
| RTP4 | 3.330094487 | 7.945173858 | 0.00173471 | up |
| CEACAM1 | 1.529970021 | 9.462737732 | 0.001762645 | up |
| NUDT16 | 1.740953039 | 7.058450616 | 0.001779482 | up |
| MAP2K6 | 1.989456336 | 7.737683574 | 0.001835649 | up |
| AGTRAP | 0.643215829 | 8.603854642 | 0.001852908 | up |
| CD300E | 0.784885822 | 10.58508992 | 0.001855487 | up |
| SPG20 | 0.682231776 | 8.4489417 | 0.001859687 | up |
| OASL | 1.68642615 | 11.21596929 | 0.001883606 | up |
| MIIP | 2.171671108 | 4.451920684 | 0.001954046 | up |
| SIGLEC5 | 0.705071344 | 11.02137276 | 0.001980701 | up |
| FMNL2 | 3.326531827 | 5.766299342 | 0.002002389 | up |
| SLFN12 | 2.879688984 | 4.712659663 | 0.00201796 | up |
| ZNF385A | 1.926354668 | 3.9045859 | 0.002098586 | up |
| CMPK2 | 2.228665539 | 10.90546603 | 0.002100423 | up |
| DDAH2 | 1.916150941 | 4.060640816 | 0.002151309 | up |
| NAPA | 2.387020966 | 7.347641832 | 0.002153384 | up |
| HBQ1 | 3.158062521 | 4.848191342 | 0.002171827 | up |
| PAGE2 | 3.568015677 | 5.758551479 | 0.002197301 | up |
| IFI6 | 4.583332766 | 6.613548153 | 0.002259634 | up |
| STAB1 | 1.939913636 | 7.361917879 | 0.002297366 | up |
| RBM45 | 1.917166036 | 5.6351431 | 0.002319045 | up |
| HIST2H2AA3 | 0.630408767 | 11.10617458 | 0.002467286 | up |
| ZNF117 | 0.918553944 | 10.894347 | 0.002486935 | up |
| SS18 | 0.865209191 | 3.037400953 | 0.002493121 | up |
| HK3 | 2.106533899 | 3.587900937 | 0.002500243 | up |
| TBC1D8 | 2.758134756 | 6.439778784 | 0.002535979 | up |
| PRAM1 | 0.749445122 | 9.533287921 | 0.002569567 | up |
| XAF1 | 1.174141206 | 12.5000705 | 0.002607799 | up |
| OAS3 | 1.538067678 | 11.95952147 | 0.002636901 | up |
| CARD6 | 1.923963074 | 8.4134023 | 0.002683753 | up |
| ANKRD34B | 2.507543077 | 3.902060253 | 0.002688987 | up |
| CPEB3 | 0.683352628 | 9.9192115 | 0.002692168 | up |
| GTPBP2 | 0.703179767 | 9.829555053 | 0.00272419 | up |
| HBD | 0.842649411 | 12.54745342 | 0.002798648 | up |
| CEACAM3 | 1.064098396 | 8.125412768 | 0.002816495 | up |
| IFI44 | 1.17842855 | 12.90682945 | 0.002829559 | up |
| ANGPTL6 | 1.288303038 | 2.780106147 | 0.002844281 | up |
| RSAD2 | 2.471538244 | 11.18518684 | 0.002858799 | up |
| NTNG2 | 0.828747489 | 11.4666225 | 0.002903719 | up |
| AGRN | 3.239143529 | 7.547972005 | 0.002911772 | up |
| ACVR1B | 1.17822294 | 7.473462963 | 0.002952737 | up |
| SLC39A1 | 1.808429788 | 4.578573526 | 0.00295342 | up |
| NINJ1 | 0.739138372 | 9.363248026 | 0.002983482 | up |
| ZBTB7B | 1.738741484 | 4.190658847 | 0.003026588 | up |
| APOBEC3A | 1.556827522 | 9.664820316 | 0.003061164 | up |
| NDC80 | 2.122741443 | 6.286359495 | 0.003071334 | up |
| GRINA | 2.844001707 | 5.749014863 | 0.003087163 | up |
| SYCP3 | 2.249139872 | 5.741758095 | 0.003139062 | up |
| C5orf56 | 2.06968791 | 7.226083374 | 0.003156677 | up |
| POU2F2 | 0.797098789 | 7.973190211 | 0.00317326 | up |
| ZNF200 | 1.079948966 | 8.571940363 | 0.003176809 | up |
| AGPAT3 | 1.424347498 | 7.5284907 | 0.003223748 | up |
| CXorf21 | 2.661060661 | 6.475282168 | 0.003223801 | up |
| GYPB | 2.853664254 | 7.690154389 | 0.003234553 | up |
| PLEK2 | 3.284371126 | 3.929807274 | 0.003351686 | up |
| MT2A | 0.847096822 | 12.67888229 | 0.003352685 | up |
| OAS1 | 1.801457439 | 11.35267039 | 0.003355808 | up |
| CXCL10 | 4.197140068 | 5.613675489 | 0.003442794 | up |
| HERC5 | 2.152975991 | 10.64712285 | 0.003547245 | up |
| CTRL | 2.186558261 | 3.823668674 | 0.003552076 | up |
| CARD17 | 2.873491731 | 3.792709442 | 0.0035991 | up |
| BTN2A2 | 2.301318562 | 5.676906926 | 0.003638863 | up |
| BLVRA | 0.873556739 | 10.56185968 | 0.003711229 | up |
| IL15 | 2.080291871 | 7.477428253 | 0.003857592 | up |
| REC8 | 0.735458144 | 10.32721521 | 0.00387899 | up |
| IFIT3 | 1.67766125 | 11.78200639 | 0.003988244 | up |
| CTSL1 | 2.457860546 | 3.883610142 | 0.003993636 | up |
| NID1 | 2.383180883 | 3.744850405 | 0.004118786 | up |
| RPH3A | 2.626748612 | 3.636594053 | 0.00412736 | up |
| ALB | 1.021688174 | 1.286220224 | 0.004133937 | up |
| NIPSNAP3A | 2.158625303 | 6.915063153 | 0.00413928 | up |
| C17orf87 | 0.709442361 | 10.54939939 | 0.004194231 | up |
| TOM1 | 1.680312787 | 4.405382116 | 0.004326236 | up |
| CSF1R | 0.765603744 | 9.264047105 | 0.004366987 | up |
| APOL2 | 2.415803123 | 5.249336405 | 0.004468401 | up |
| H1F0 | 1.303195788 | 8.287983758 | 0.004473132 | up |
| CBWD5 | 2.187114688 | 7.725394284 | 0.004573 | up |
| COQ2 | 0.768673629 | 7.718126521 | 0.00460587 | up |
| LILRA6 | 0.72882475 | 11.30496203 | 0.004619154 | up |
| MAFG | 2.274247814 | 6.098831911 | 0.004687572 | up |
| TMEM45A | 2.249164388 | 3.828522147 | 0.00478906 | up |
| MOBKL2C | 1.626791388 | 6.807691384 | 0.004944715 | up |
| STXBP2 | 1.679389598 | 7.593907016 | 0.005010249 | up |
| ZNF438 | 1.569259783 | 3.675205132 | 0.00508547 | up |
| GORASP1 | 1.2521918 | 8.211827984 | 0.005183815 | up |
| LILRB4 | 1.1998088 | 9.596439684 | 0.005204569 | up |
| TCN1 | 2.845256377 | 4.686588711 | 0.00525565 | up |
| C3orf14 | 1.906809364 | 3.525557268 | 0.005265112 | up |
| RBCK1 | 0.865396649 | 9.154594968 | 0.005346588 | up |
| TRIM38 | 0.68337555 | 10.42059566 | 0.005389635 | up |
| SPTA1 | 1.672411054 | 3.2884676 | 0.005413605 | up |
| LOC162632 | 1.509571277 | 8.278937468 | 0.005419643 | up |
| TRIM6 | 3.585119897 | 5.330894058 | 0.005573992 | up |
| SLC26A6 | 2.40594857 | 6.178637179 | 0.005688008 | up |
| NCF1 | 0.594457039 | 11.88794776 | 0.005701004 | up |
| LAP3 | 1.547795133 | 9.657535316 | 0.005711579 | up |
| FUT4 | 1.617668422 | 7.168827389 | 0.005823977 | up |
| NBPF9 | 2.411567219 | 4.970156184 | 0.005826432 | up |
| ISG15 | 1.673003211 | 11.94326411 | 0.005863052 | up |
| SERPING1 | 3.761861766 | 6.639629611 | 0.006071185 | up |
| IFIT1 | 1.577582072 | 12.72181187 | 0.006136936 | up |
| GATA1 | 2.356030633 | 4.180993211 | 0.006210747 | up |
| PLSCR1 | 1.102747478 | 11.67521137 | 0.006464644 | up |
| ADM | 0.7056598 | 11.38961611 | 0.006505317 | up |
| MS4A14 | 2.799159218 | 6.106593384 | 0.006534077 | up |
| CDK11A | 1.824615067 | 9.481067947 | 0.006563379 | up |
| DUSP1 | 0.669020806 | 9.839910342 | 0.006606173 | up |
| CD36 | 1.071663607 | 11.07423419 | 0.006634286 | up |
| MRPL44 | 0.741288072 | 8.453704605 | 0.006654111 | up |
| TNNT3 | 1.900955193 | 4.6807299 | 0.006693926 | up |
| LOC200030 | 2.288303782 | 6.349377111 | 0.006745454 | up |
| IFITM3 | 0.8075078 | 13.51539289 | 0.006836157 | up |
| VEGFA | 1.292142572 | 9.143962684 | 0.00686677 | up |
| TRIM10 | 3.079234386 | 7.159455079 | 0.006994495 | up |
| LYSMD2 | 0.808478939 | 9.484234658 | 0.007037589 | up |
| MCTP1 | 0.705306233 | 11.03392753 | 0.007068624 | up |
| SHARPIN | 2.409407363 | 4.844913195 | 0.007140404 | up |
| FRMD3 | 0.918576444 | 9.917458658 | 0.007298131 | up |
| SLC26A8 | 2.5542082 | 4.145735795 | 0.007327253 | up |
| VASP | 1.870702054 | 3.746107416 | 0.007453303 | up |
| WIPI1 | 0.755121522 | 9.543720842 | 0.007462926 | up |
| MARCO | 0.893344843 | 2.175864137 | 0.007507715 | up |
| SPATA1 | 1.288302758 | 3.555640395 | 0.007518322 | up |
| EPB41L3 | 0.625000778 | 11.271108 | 0.007641951 | up |
| PNPT1 | 2.686610687 | 8.053831442 | 0.00765732 | up |
| CCR1 | 0.929158817 | 10.51000271 | 0.00791157 | up |
| SUSD1 | 0.674989829 | 8.662861584 | 0.00802539 | up |
| MX1 | 1.343390094 | 12.11974853 | 0.008048895 | up |
| RNF135 | 0.681354678 | 9.522646053 | 0.008059192 | up |
| GMPR | 1.29023205 | 10.3540885 | 0.008132744 | up |
| BCL2L1 | 2.621019374 | 4.108053063 | 0.008158865 | up |
| IP6K1 | 1.21850796 | 3.392680921 | 0.00818326 | up |
| P2RX1 | 2.42851318 | 6.376563468 | 0.008203934 | up |
| XRCC4 | 0.664426331 | 7.975205842 | 0.008220186 | up |
| ADAM9 | 0.654085304 | 9.122379021 | 0.008475366 | up |
| EPSTI1 | 2.279560244 | 10.66186739 | 0.008517791 | up |
| MR1 | 1.715098604 | 6.998739468 | 0.008743086 | up |
| TTC25 | 2.506985302 | 3.941196174 | 0.00876163 | up |
| SLC36A1 | 0.88967695 | 9.294471974 | 0.008801774 | up |
| IFIT5 | 0.970667039 | 12.270335 | 0.008908151 | up |
| DDX58 | 1.173700567 | 10.75985203 | 0.008937429 | up |
| GRIPAP1 | 0.597985778 | 10.35652442 | 0.00899681 | up |
| TFPI | 2.437268037 | 5.314118516 | 0.009004219 | up |
| HPS1 | 2.719742971 | 5.680404842 | 0.009057103 | up |
| C1GALT1 | 1.613282102 | 7.605580279 | 0.009304747 | up |
| CCL3 | 1.760441629 | 3.643133963 | 0.009479869 | up |
| LILRA5 | 2.645299232 | 8.267796468 | 0.009488048 | up |
| LPAR6 | 0.605646739 | 10.94813345 | 0.009767705 | up |
| CFD | 2.007130608 | 3.189639847 | 0.01004643 | up |
| TREX1 | 2.558384131 | 6.939623247 | 0.010137933 | up |
| TRIM34 | 1.328922323 | 3.474609232 | 0.010294523 | up |
| HIST1H2AE | 0.938405278 | 10.31433176 | 0.010329323 | up |
| HIST1H2BD | 0.679888111 | 10.48717026 | 0.010365048 | up |
| IFI35 | 1.012881478 | 11.52928926 | 0.010509663 | up |
| SNX20 | 1.176738831 | 3.344419084 | 0.010831124 | up |
| RELT | 1.778064856 | 6.951741547 | 0.011134629 | up |
| UBA7 | 0.680048622 | 9.520654895 | 0.011155367 | up |
| LILRB3 | 0.611731528 | 9.806185816 | 0.011169142 | up |
| OAF | 0.732597253 | 2.882274332 | 0.0111693 | up |
| APOB48R | 1.597157031 | 4.327349647 | 0.011294291 | up |
| NEURL | 0.88864147 | 2.720802963 | 0.011336004 | up |
| CA1 | 1.570810767 | 9.510151658 | 0.011394036 | up |
| EIF2AK2 | 0.609060656 | 12.62360032 | 0.011558555 | up |
| C19orf66 | 0.861919167 | 9.73533 | 0.011778945 | up |
| RIN2 | 0.835394689 | 11.16948797 | 0.011858036 | up |
| DDX60 | 1.049357122 | 11.619932 | 0.011922719 | up |
| PLEC | 0.854017456 | 3.319957184 | 0.01194816 | up |
| SAMD9 | 0.6170544 | 11.19442353 | 0.011968813 | up |
| LHPP | 1.723976653 | 3.8373681 | 0.011971548 | up |
| NAGA | 0.794288486 | 8.330691184 | 0.012094339 | up |
| PQLC1 | 1.743988758 | 7.372580758 | 0.012105797 | up |
| NME4 | 1.982711096 | 4.0535004 | 0.012131437 | up |
| IFFO1 | 2.254484668 | 5.6124635 | 0.012251003 | up |
| TMEM179B | 0.732577176 | 7.865049021 | 0.012276679 | up |
| LGALS3 | 0.685328744 | 11.95554761 | 0.012298223 | up |
| SRGAP2 | 0.930340183 | 9.694457105 | 0.012523635 | up |
| CARD9 | 1.573249583 | 3.548753737 | 0.012648329 | up |
| ARID3A | 1.452997292 | 3.358652805 | 0.012709981 | up |
| GPBAR1 | 0.717353437 | 2.648077126 | 0.012740546 | up |
| APOL1 | 0.7018945 | 9.563090842 | 0.01306207 | up |
| HBM | 1.251145614 | 11.41345525 | 0.013164311 | up |
| LMNB1 | 1.00932197 | 9.009121511 | 0.01324945 | up |
| PCK2 | 1.845346119 | 4.166668189 | 0.013293523 | up |
| RNASE2 | 0.846438133 | 11.57331358 | 0.013307324 | up |
| RNF185 | 1.682832921 | 6.404942774 | 0.013504552 | up |
| KLHDC8B | 1.651250242 | 3.439715047 | 0.013525168 | up |
| MYL4 | 0.696215622 | 12.12982995 | 0.013536529 | up |
| PTPRO | 2.23414019 | 5.674214942 | 0.013574945 | up |
| CASP10 | 0.75436404 | 8.408115795 | 0.013693625 | up |
| NT5C3 | 0.697330144 | 12.16614558 | 0.013712593 | up |
| OAS2 | 0.984869178 | 12.22935758 | 0.013728021 | up |
| SENP3 | 2.090525294 | 5.431394074 | 0.013957979 | up |
| PARP12 | 0.593228433 | 12.67569384 | 0.014030743 | up |
| ZCCHC2 | 0.774343111 | 11.59757911 | 0.014082977 | up |
| EMR1 | 0.788103828 | 9.308829789 | 0.014152685 | up |
| DCLRE1B | 1.30941273 | 3.825598505 | 0.014497972 | up |
| DUSP3 | 1.224823881 | 8.366434963 | 0.014552472 | up |
| KCNH7 | 1.278569448 | 2.561777389 | 0.014569281 | up |
| USP18 | 4.072042366 | 5.440209163 | 0.014713117 | up |
| RAB20 | 2.127840422 | 3.342569911 | 0.01480185 | up |
| LRPAP1 | 1.353270872 | 7.028231395 | 0.014821805 | up |
| HBG1 | 1.051909472 | 13.79583166 | 0.014866724 | up |
| OSBP2 | 3.11297267 | 6.274501632 | 0.014888829 | up |
| TNK2 | 0.870626904 | 9.456305758 | 0.014937362 | up |
| ADAMTSL4 | 1.363748424 | 3.444571716 | 0.015231108 | up |
| NEXN | 1.795283437 | 8.532220147 | 0.015541816 | up |
| SAMD9L | 0.928373122 | 11.31794329 | 0.015600012 | up |
| DNTTIP1 | 0.630358957 | 8.469015795 | 0.015705396 | up |
| ALAS1 | 1.51104011 | 4.675315032 | 0.015797273 | up |
| FAM40A | 1.628218102 | 4.275762526 | 0.015941934 | up |
| C4orf33 | 1.621548386 | 8.349818037 | 0.016094789 | up |
| RIPK3 | 1.971732072 | 5.5571875 | 0.016149531 | up |
| HERC6 | 2.32950116 | 8.537768358 | 0.016159843 | up |
| TNNT1 | 2.577323662 | 4.118719084 | 0.016443046 | up |
| PAPLN | 1.039068657 | 3.596407526 | 0.016500027 | up |
| SIGLEC9 | 1.890611428 | 5.084615032 | 0.016607849 | up |
| EXOC7 | 0.656643163 | 8.431813168 | 0.016625351 | up |
| KIAA0319L | 0.976097822 | 9.347757526 | 0.016732665 | up |
| PDLIM7 | 0.678526789 | 8.905816526 | 0.016787096 | up |
| DRAP1 | 0.687002133 | 9.909358763 | 0.016791042 | up |
| PPCDC | 0.643580979 | 9.321280058 | 0.017066651 | up |
| HBG1 | 0.785562717 | 13.85196261 | 0.017089003 | up |
| DCAF11 | 1.528913129 | 6.810863268 | 0.017202153 | up |
| CPT1A | 2.226861034 | 7.617985821 | 0.01729578 | up |
| KIAA1539 | 0.62975004 | 8.717531111 | 0.01751719 | up |
| NMNAT3 | 1.472413516 | 3.284947074 | 0.017520195 | up |
| CCDC21 | 1.026606957 | 3.294531416 | 0.017665206 | up |
| RHBDF2 | 1.880800833 | 6.532244158 | 0.017853318 | up |
| FCGR1B | 1.286519819 | 9.311549195 | 0.018010647 | up |
| RNF31 | 1.16626696 | 7.878297784 | 0.018213404 | up |
| SRD5A1 | 1.914035036 | 6.838511995 | 0.018221652 | up |
| CES1 | 2.865188489 | 7.538569047 | 0.018438803 | up |
| FBXO6 | 2.131100318 | 3.854272132 | 0.018492813 | up |
| CD68 | 1.009018633 | 10.37066989 | 0.018549378 | up |
| ASGR2 | 1.705582066 | 7.275645984 | 0.018718237 | up |
| SLC25A16 | 1.403826263 | 6.490421305 | 0.019064879 | up |
| SSH3 | 1.67535879 | 4.228672516 | 0.019103028 | up |
| RANBP10 | 2.336960443 | 5.239562353 | 0.019357 | up |
| ATP5E | 1.268873526 | 7.244103784 | 0.019361005 | up |
| TMEM86B | 0.8571002 | 2.702316432 | 0.019533012 | up |
| HSH2D | 0.651312378 | 11.08291279 | 0.019683584 | up |
| IGF2BP3 | 2.250485402 | 4.412221221 | 0.01981615 | up |
| TCN2 | 0.817548126 | 1.968316168 | 0.019883732 | up |
| RARA | 0.754341278 | 8.172676053 | 0.019887906 | up |
| NFKBIE | 1.535554897 | 3.592339379 | 0.019921361 | up |
| LTBR | 1.385653029 | 6.908007421 | 0.0200626 | up |
| PLIN3 | 1.545404371 | 4.208849989 | 0.020230091 | up |
| FAR2 | 1.651106213 | 7.921213063 | 0.020255591 | up |
| ADCY4 | 2.110640663 | 5.463982326 | 0.020412599 | up |
| SPATS2L | 3.385288981 | 6.119872268 | 0.020436395 | up |
| DDI2 | 0.654335203 | 8.719992884 | 0.020920565 | up |
| KLF4 | 0.880530772 | 8.838509974 | 0.021075197 | up |
| C19orf42 | 1.468408842 | 6.573049063 | 0.021333164 | up |
| LY6E | 3.066052424 | 7.502702742 | 0.021449259 | up |
| TDRD7 | 0.749050589 | 10.49061624 | 0.021626838 | up |
| SMARCD3 | 0.889961717 | 8.834116763 | 0.021846638 | up |
| FLVCR2 | 1.071380983 | 3.075675305 | 0.021869943 | up |
| IFT74 | 2.385716079 | 8.067276247 | 0.021900223 | up |
| FBP1 | 2.016034047 | 6.333674416 | 0.021912574 | up |
| IFIH1 | 0.855478367 | 11.65396789 | 0.022144675 | up |
| IL17RA | 1.457433458 | 7.367180737 | 0.022300778 | up |
| ZNF701 | 1.176009959 | 3.807584779 | 0.022483224 | up |
| SMPDL3A | 2.380772511 | 6.368216447 | 0.022653069 | up |
| SLC6A8 | 2.250541016 | 4.201962484 | 0.022730593 | up |
| PLCB3 | 1.817602232 | 6.222262268 | 0.023248514 | up |
| C5orf62 | 2.102029487 | 6.471124658 | 0.023431688 | up |
| CCDC146 | 1.949925942 | 6.158467411 | 0.023522192 | up |
| GOLGA6L4 | 2.445509984 | 5.310864426 | 0.023955423 | up |
| PDCD1LG2 | 1.770591007 | 2.941306463 | 0.024087367 | up |
| NFKB2 | 1.251400707 | 3.354844016 | 0.024252489 | up |
| NCKAP5L | 1.054143786 | 5.480660437 | 0.02431772 | up |
| TRMT2A | 1.336898547 | 3.965474753 | 0.024366918 | up |
| CAMK1 | 0.914932993 | 3.107073653 | 0.024489676 | up |
| FXYD6 | 1.263050947 | 2.655340211 | 0.02455875 | up |
| ITLN1 | 2.173711886 | 4.472964868 | 0.02467243 | up |
| CEBPA | 0.645225361 | 8.732566816 | 0.025180575 | up |
| OGFR | 1.572997273 | 6.236920732 | 0.025252052 | up |
| SIRPD | 1.67703342 | 3.560012132 | 0.025462627 | up |
| NPEPL1 | 1.497378862 | 6.969711384 | 0.02615483 | up |
| ACTA2 | 0.745405233 | 2.922216089 | 0.026371535 | up |
| ZFYVE26 | 0.79364179 | 7.974073095 | 0.026494065 | up |
| CDK14 | 0.680365833 | 9.827655053 | 0.026854699 | up |
| BPGM | 1.02670885 | 10.33283592 | 0.026903324 | up |
| SLC1A5 | 1.388048836 | 3.899473437 | 0.027026226 | up |
| TTLL3 | 1.769294627 | 4.591432668 | 0.027043834 | up |
| EFEMP2 | 1.791392978 | 4.707501668 | 0.027445357 | up |
| GABBR1 | 0.691777093 | 10.18043163 | 0.027462624 | up |
| C15orf52 | 1.574763756 | 3.723526626 | 0.027499793 | up |
| LIG4 | 1.720875903 | 6.805197574 | 0.02761378 | up |
| NUSAP1 | 2.241112204 | 5.848267505 | 0.027886558 | up |
| E2F2 | 0.674708333 | 11.78051468 | 0.027903498 | up |
| SIRT2 | 0.665448042 | 3.499510811 | 0.027937088 | up |
| BCL3 | 0.588234589 | 9.376170737 | 0.027989656 | up |
| C19orf28 | 1.72254032 | 5.699354232 | 0.028020518 | up |
| ARL5B | 1.86029084 | 5.057253158 | 0.028092055 | up |
| IRF5 | 2.314789918 | 9.962925295 | 0.029075443 | up |
| LCN2 | 1.531969524 | 9.023401695 | 0.029134972 | up |
| ERI2 | 0.829451097 | 3.317352358 | 0.029312937 | up |
| ANKDD1A | 1.766850578 | 5.416561921 | 0.029356595 | up |
| TRIM5 | 0.685659033 | 10.73905616 | 0.029888638 | up |
| SLC22A15 | 1.527832581 | 7.907521 | 0.030932816 | up |
| CBWD1 | 1.721475408 | 6.548994089 | 0.030976845 | up |
| LRRC33 | 0.838566344 | 3.334216737 | 0.031034121 | up |
| SIDT2 | 1.976187499 | 6.687339205 | 0.031314324 | up |
| TADA2A | 1.16152824 | 3.465127047 | 0.031437209 | up |
| SNTB1 | 0.659432361 | 9.936087447 | 0.03217368 | up |
| PRRG4 | 1.492635924 | 7.845749989 | 0.032200695 | up |
| FAM20C | 1.944105463 | 6.323924432 | 0.032246659 | up |
| FAM164A | 1.083703177 | 8.087917574 | 0.032319793 | up |
| TP53I3 | 0.938796044 | 3.075178463 | 0.032465741 | up |
| TGM2 | 1.113998377 | 2.569328032 | 0.032572863 | up |
| GPD2 | 0.606310694 | 9.696671553 | 0.032657466 | up |
| HSD17B13 | 0.706110392 | 2.685138574 | 0.032768046 | up |
| LOC643332 | 0.811228111 | 11.37440495 | 0.03277125 | up |
| NUDT1 | 1.697409873 | 4.639315916 | 0.032960455 | up |
| ADPRH | 1.849680821 | 4.781222289 | 0.033258538 | up |
| CAMTA2 | 1.699448236 | 5.027896516 | 0.033591026 | up |
| TMLHE | 1.316676671 | 4.186060289 | 0.033740022 | up |
| STRADA | 1.221947704 | 7.009646474 | 0.033843215 | up |
| LTB4R | 1.270823472 | 8.672792763 | 0.033867364 | up |
| CCDC18 | 1.661732567 | 6.551138179 | 0.033977925 | up |
| TMEM55B | 1.519110054 | 6.583400437 | 0.034026019 | up |
| DOCK4 | 1.297239902 | 9.385203379 | 0.034145985 | up |
| BTNL8 | 0.693479833 | 9.708001447 | 0.034318115 | up |
| NOTCH2NL | 0.69067848 | 8.517176011 | 0.034374379 | up |
| CCDC23 | 1.550915662 | 5.117374321 | 0.034645194 | up |
| NEIL3 | 2.576425102 | 7.788970611 | 0.03471729 | up |
| LRG1 | 0.730429433 | 9.787289289 | 0.036465256 | up |
| LOC653562 | 2.15459158 | 3.849663632 | 0.036643178 | up |
| FAM26F | 0.596471778 | 11.90734776 | 0.037076299 | up |
| TRIM69 | 1.035023332 | 3.0305151 | 0.037228861 | up |
| ZNRF1 | 1.046730318 | 3.233255684 | 0.037444376 | up |
| NT5M | 0.962026192 | 3.198758547 | 0.037595593 | up |
| LPP | 0.600545094 | 9.501831079 | 0.03811354 | up |
| C9orf40 | 1.45714769 | 3.908456163 | 0.038155021 | up |
| PDZD8 | 1.228293287 | 3.382334716 | 0.03952142 | up |
| FNTB | 1.94981952 | 7.2030233 | 0.039569422 | up |
| ASNA1 | 1.25795235 | 4.106438016 | 0.039632185 | up |
| TRIM7 | 0.910837394 | 3.103986242 | 0.039702493 | up |
| ABCG2 | 2.481062306 | 3.088028509 | 0.03981466 | up |
| TOLLIP | 1.426232616 | 4.002292311 | 0.040067313 | up |
| AIM2 | 0.779957683 | 11.03603729 | 0.040162055 | up |
| SECTM1 | 0.603571261 | 9.032146026 | 0.040377187 | up |
| ZNF684 | 1.993708768 | 3.863142274 | 0.04077788 | up |
| ATF5 | 1.514126708 | 3.396747084 | 0.041311279 | up |
| HMBS | 2.1079564 | 6.340602163 | 0.04170678 | up |
| MAN2B1 | 0.585227953 | 7.680618921 | 0.042019411 | up |
| C12orf4 | 0.701011758 | 7.973478421 | 0.04205877 | up |
| WBP4 | 1.219631717 | 6.299233037 | 0.042256181 | up |
| SDHAP2 | 1.131727713 | 4.198276468 | 0.042422344 | up |
| SGK494 | 1.693374566 | 5.203518084 | 0.042439851 | up |
| CA2 | 0.671147822 | 10.38763453 | 0.042513675 | up |
| LQK1 | 1.555059584 | 3.500997932 | 0.043109844 | up |
| DNPEP | 1.418561296 | 5.155748447 | 0.043297372 | up |
| KIAA1598 | 0.795720429 | 9.691045663 | 0.043412564 | up |
| NUDT4 | 1.943432611 | 3.744020442 | 0.044130468 | up |
| ANK1 | 2.460894768 | 7.507113805 | 0.044131535 | up |
| FZD1 | 1.392176293 | 4.232464368 | 0.044264371 | up |
| ROPN1L | 1.737821303 | 6.016654142 | 0.044376578 | up |
| PLOD1 | 0.765034658 | 3.296729858 | 0.044790212 | up |
| TST | 1.386999828 | 4.300004368 | 0.045520101 | up |
| MASTL | 1.788425481 | 4.490089942 | 0.045821526 | up |
| FLJ36031 | 0.939046697 | 7.656820463 | 0.046371348 | up |
| KIF1C | 0.998640083 | 7.444431989 | 0.046525611 | up |
| SBNO2 | 1.140117531 | 3.498231053 | 0.046691651 | up |
| CPT1B | 1.955730311 | 5.817442468 | 0.046747866 | up |
| TRIM14 | 1.176309489 | 8.331846932 | 0.046818555 | up |
| ERAP2 | 0.814793783 | 10.5894175 | 0.047206298 | up |
| NRM | 1.432839826 | 4.935289532 | 0.047263475 | up |
| PHKG1 | 2.231447141 | 3.648582747 | 0.047502077 | up |
| FAM100B | 0.998079401 | 3.424646263 | 0.047779303 | up |
| ARHGAP6 | 1.760478461 | 3.6866367 | 0.047794959 | up |
| FTSJD2 | 0.6526903 | 10.06604129 | 0.047884582 | up |
| PPP4C | 0.684257609 | 8.060495605 | 0.047920996 | up |
| PTPRN2 | 1.65747633 | 7.886165137 | 0.048447703 | up |
| DISC1 | 0.744050759 | 9.331450226 | 0.048772819 | up |
| SERTAD3 | 1.444225356 | 6.089803847 | 0.048938123 | up |
| LSMD1 | 1.328058561 | 5.129680221 | 0.048964845 | up |
| RNASE1 | 1.121257086 | 2.785979947 | 0.049226569 | up |
| SNORA41 | 1.597886546 | 5.674843737 | 0.049303766 | up |
| UVRAG | 1.108739447 | 5.583291589 | 0.049390964 | up |
| SLC29A1 | 1.919408306 | 6.007204558 | 0.049485356 | up |
| CXXC1 | 0.846488984 | 3.572047826 | 0.049939862 | up |
| TOM1L1 | -1.335491892 | 2.594322847 | 5.14E-08 | down |
| SLAIN1 | -0.962516522 | 9.546341421 | 1.02E-07 | down |
| FBL | -0.700654006 | 12.18582921 | 1.03E-07 | down |
| SPOCK2 | -0.872200061 | 11.87964555 | 1.50E-07 | down |
| DYRK2 | -0.741792378 | 11.48796776 | 4.71E-07 | down |
| GMPS | -0.802358422 | 9.668888684 | 5.39E-07 | down |
| MOAP1 | -0.744859006 | 10.4202645 | 1.15E-06 | down |
| SLC38A1 | -0.634068206 | 12.88085955 | 1.39E-06 | down |
| GOT2 | -0.815919411 | 9.864031658 | 1.60E-06 | down |
| FAM102A | -0.806119617 | 12.10801982 | 1.63E-06 | down |
| NR3C2 | -3.431999611 | 6.032642774 | 1.96E-06 | down |
| ZNF607 | -0.605814696 | 1.569817934 | 2.29E-06 | down |
| NMT2 | -1.287843939 | 9.389148511 | 2.67E-06 | down |
| ITGA6 | -0.695145161 | 12.17801276 | 2.82E-06 | down |
| CD3G | -0.853965072 | 10.41092858 | 3.59E-06 | down |
| CXCR6 | -4.530002481 | 6.500640021 | 3.90E-06 | down |
| SPON1 | -0.815201786 | 1.839610816 | 4.49E-06 | down |
| KLRB1 | -0.979705583 | 12.46205218 | 4.54E-06 | down |
| ZMYND11 | -0.673741756 | 11.06633737 | 4.65E-06 | down |
| TTC3 | -0.6171029 | 11.486403 | 5.95E-06 | down |
| DPH5 | -0.787397833 | 10.35411858 | 6.23E-06 | down |
| USP14 | -0.638510678 | 10.59151147 | 7.23E-06 | down |
| RRAS2 | -0.847302933 | 10.60789658 | 8.46E-06 | down |
| ANKRD46 | -0.78783555 | 9.343543289 | 8.80E-06 | down |
| SLC4A10 | -5.050298921 | 5.332333453 | 9.25E-06 | down |
| ATIC | -0.9011879 | 9.929195421 | 9.80E-06 | down |
| OCIAD2 | -0.957198756 | 9.899181263 | 9.96E-06 | down |
| PREPL | -0.699839317 | 10.81642132 | 1.02E-05 | down |
| HLTF | -0.683175233 | 10.48515458 | 1.03E-05 | down |
| PIK3IP1 | -0.830391044 | 10.81044897 | 1.04E-05 | down |
| ZFP82 | -2.891899456 | 7.015125889 | 1.06E-05 | down |
| RHOH | -0.853778889 | 10.24805642 | 1.06E-05 | down |
| MAP9 | -3.308266084 | 5.770046832 | 1.16E-05 | down |
| ITK | -0.682092311 | 12.13258184 | 1.19E-05 | down |
| DOCK9 | -0.803536767 | 10.73282842 | 1.27E-05 | down |
| ZNF2 | -0.808431472 | 9.615743026 | 1.30E-05 | down |
| PTPLAD1 | -0.958253056 | 9.499967658 | 1.46E-05 | down |
| MRPS17 | -0.740996089 | 10.35989955 | 1.55E-05 | down |
| ZNF395 | -0.794271361 | 10.16928545 | 1.62E-05 | down |
| RSL1D1 | -0.613107433 | 11.45169579 | 1.70E-05 | down |
| C12orf23 | -0.826831928 | 10.24918192 | 1.77E-05 | down |
| BCL2 | -0.598285094 | 11.26275245 | 1.83E-05 | down |
| PFAS | -0.926970283 | 9.356919763 | 1.83E-05 | down |
| LRRN3 | -4.561638983 | 6.050586889 | 2.03E-05 | down |
| CD2 | -0.610580622 | 11.570848 | 2.23E-05 | down |
| ZNF260 | -0.772711422 | 10.0323565 | 2.24E-05 | down |
| TCF7 | -0.646618883 | 12.40565039 | 2.37E-05 | down |
| SGK223 | -2.823624963 | 3.857309568 | 2.41E-05 | down |
| NELL2 | -1.296723394 | 10.58083355 | 2.43E-05 | down |
| SNHG8 | -0.715196817 | 11.63809871 | 2.46E-05 | down |
| GLO1 | -0.637211989 | 9.928923842 | 2.52E-05 | down |
| FAIM3 | -0.767500678 | 10.72746795 | 2.71E-05 | down |
| AMOT | -0.701403022 | 2.267569263 | 2.82E-05 | down |
| RFC1 | -0.609399461 | 9.702447447 | 2.98E-05 | down |
| OXNAD1 | -0.685038067 | 11.43385868 | 3.22E-05 | down |
| FAM98B | -0.677462833 | 9.973582053 | 3.35E-05 | down |
| EPRS | -0.647571544 | 9.616125684 | 3.43E-05 | down |
| SCAMP3 | -0.751507322 | 8.638875947 | 3.61E-05 | down |
| SIRPG | -1.021885067 | 10.19997058 | 3.86E-05 | down |
| THEMIS | -0.758834378 | 11.85051674 | 3.88E-05 | down |
| C12orf57 | -0.905966744 | 9.619196658 | 3.91E-05 | down |
| TMEM30B | -0.7512692 | 2.414766742 | 4.07E-05 | down |
| CHIC1 | -0.6638833 | 10.62134426 | 4.24E-05 | down |
| DSEL | -0.906641253 | 1.718373974 | 4.25E-05 | down |
| ABHD14B | -1.047978033 | 9.270435553 | 4.31E-05 | down |
| MYST4 | -0.729729767 | 10.40507413 | 4.52E-05 | down |
| CHMP7 | -0.631396078 | 10.65401695 | 4.66E-05 | down |
| IL24 | -0.807129072 | 10.77725705 | 4.86E-05 | down |
| BCL11B | -0.673478967 | 11.76094645 | 4.90E-05 | down |
| TMEM48 | -0.911745156 | 9.134728516 | 5.21E-05 | down |
| ARID5B | -0.595597278 | 11.01786747 | 5.41E-05 | down |
| BLMH | -0.61299295 | 9.753403868 | 5.57E-05 | down |
| TMEM209 | -0.637986883 | 10.52236824 | 5.83E-05 | down |
| SLC39A14 | -2.845498306 | 7.169899932 | 6.37E-05 | down |
| RLN1 | -0.711630627 | 1.007286478 | 6.47E-05 | down |
| LYRM7 | -1.017630411 | 9.575377342 | 6.57E-05 | down |
| ZNF781 | -0.705345468 | 2.013453579 | 6.71E-05 | down |
| SKAP1 | -0.837912261 | 10.96502734 | 6.72E-05 | down |
| NPAT | -0.66156445 | 9.931611816 | 6.80E-05 | down |
| TMEM99 | -2.891443698 | 5.210745495 | 7.19E-05 | down |
| SEC22A | -0.696106528 | 8.702649279 | 7.69E-05 | down |
| SHQ1 | -0.600381644 | 9.477437395 | 8.02E-05 | down |
| ZNF518B | -0.725625006 | 9.903612342 | 8.35E-05 | down |
| IL7R | -1.218326278 | 11.73905808 | 8.52E-05 | down |
| MTUS1 | -0.873302743 | 2.032480129 | 8.66E-05 | down |
| C11orf1 | -1.696791994 | 8.336308474 | 9.02E-05 | down |
| B3GALT2 | -1.008690624 | 1.872543518 | 9.62E-05 | down |
| PLS1 | -0.667102953 | 1.840796024 | 9.83E-05 | down |
| PEBP1 | -0.795614322 | 11.05228216 | 0.000101826 | down |
| RORC | -0.634344144 | 2.233014295 | 0.000104975 | down |
| MSH2 | -2.068236724 | 7.2129221 | 0.00011262 | down |
| ZNF764 | -0.786232456 | 9.340756 | 0.000114189 | down |
| ZFP30 | -1.264765638 | 2.963261747 | 0.000117336 | down |
| RCAN3 | -0.925158206 | 10.27434582 | 0.000120124 | down |
| ARL6 | -0.765861911 | 2.191092105 | 0.000125856 | down |
| RAB39B | -2.419428137 | 6.671858363 | 0.000129783 | down |
| USP36 | -0.7355767 | 10.59819663 | 0.000133008 | down |
| NR1D2 | -0.612710233 | 11.37880124 | 0.000143773 | down |
| ADAM12 | -0.813365237 | 2.0890365 | 0.000144552 | down |
| SLC25A23 | -2.929067712 | 5.129785663 | 0.00015192 | down |
| GRAMD3 | -1.083616822 | 8.115238321 | 0.000153867 | down |
| LBH | -0.824646772 | 10.84076889 | 0.000159983 | down |
| NUCB2 | -0.622196711 | 10.93209684 | 0.000161829 | down |
| MAGED1 | -2.132602062 | 7.550390716 | 0.000172054 | down |
| RNGTT | -0.617998672 | 10.76623674 | 0.00017296 | down |
| CD96 | -1.3894764 | 8.931129668 | 0.000188377 | down |
| KIAA1430 | -0.689868339 | 9.798712211 | 0.000192746 | down |
| IPO5 | -0.637914 | 9.531467211 | 0.000199179 | down |
| PECI | -0.772356806 | 9.720871026 | 0.00020014 | down |
| ATP8A2 | -0.708510838 | 1.572204963 | 0.000203647 | down |
| ZNF146 | -0.618592867 | 11.56468653 | 0.000206705 | down |
| ANKRD19 | -0.691285561 | 10.17855489 | 0.000208386 | down |
| CD8A | -0.730494933 | 13.19044418 | 0.00020986 | down |
| CD27 | -0.860578806 | 9.860252658 | 0.000212874 | down |
| MID2 | -0.737277608 | 2.410962668 | 0.000213871 | down |
| PATZ1 | -0.777528806 | 10.05804613 | 0.000214008 | down |
| PLEKHA1 | -0.629498917 | 11.01559155 | 0.000224348 | down |
| KIAA0802 | -0.68613363 | 1.389791313 | 0.000225081 | down |
| TMEM41A | -0.908423517 | 8.388375674 | 0.000229434 | down |
| MUTED | -0.713453989 | 9.772248711 | 0.000229942 | down |
| ZNF354C | -0.655557228 | 9.827582342 | 0.000232932 | down |
| PPIH | -0.692440911 | 9.663692974 | 0.000234936 | down |
| LDOC1L | -0.611691856 | 10.91719853 | 0.00023633 | down |
| ATF7IP2 | -0.692623628 | 10.38390347 | 0.000236524 | down |
| MBLAC2 | -0.804358467 | 8.591191495 | 0.000246322 | down |
| PLAG1 | -2.85057491 | 6.031661584 | 0.000252934 | down |
| ZNF256 | -0.676881089 | 2.630835316 | 0.000262496 | down |
| SLAMF6 | -0.617857917 | 10.88544571 | 0.000274534 | down |
| VPRBP | -1.025705422 | 8.821555637 | 0.000290276 | down |
| SESN1 | -1.015512933 | 9.619329958 | 0.000299237 | down |
| CA6 | -0.733419629 | 2.400217542 | 0.000299404 | down |
| METTL8 | -2.7185677 | 6.731565705 | 0.000313536 | down |
| CD28 | -0.623965411 | 11.12428574 | 0.000314324 | down |
| GNPNAT1 | -0.616992178 | 9.541761447 | 0.000326957 | down |
| ZNF331 | -0.821525217 | 8.901325026 | 0.000327594 | down |
| ZNF559 | -0.705656256 | 10.44820447 | 0.000345404 | down |
| PRPS1 | -2.256968977 | 6.561239042 | 0.000354178 | down |
| APBA2 | -1.583914911 | 9.462464616 | 0.000356912 | down |
| IARS2 | -0.587510156 | 9.715112316 | 0.000359228 | down |
| PRKCQ | -0.814549567 | 9.752509632 | 0.000363234 | down |
| IFFO2 | -0.727734359 | 2.683382295 | 0.000367426 | down |
| C14orf126 | -0.651962006 | 8.673604742 | 0.00038093 | down |
| USP13 | -2.143361317 | 3.826881637 | 0.000387377 | down |
| BEX2 | -3.052520679 | 4.566464242 | 0.00039029 | down |
| TACR3 | -0.639182891 | 0.913423437 | 0.000399397 | down |
| CCR7 | -1.112292111 | 10.09219229 | 0.00040142 | down |
| PDE6B | -0.7658581 | 8.910815211 | 0.000407622 | down |
| MGAT4A | -0.611827161 | 11.54720468 | 0.000408324 | down |
| GRWD1 | -0.850958144 | 9.006025463 | 0.000422427 | down |
| MRPS24 | -0.640933733 | 10.55206421 | 0.000426043 | down |
| TMEM117 | -0.756136993 | 1.648320425 | 0.000440191 | down |
| NUBPL | -2.434376059 | 5.200283368 | 0.000450235 | down |
| FBXO21 | -0.587198111 | 9.413589026 | 0.000456917 | down |
| IMPDH2 | -0.772380311 | 10.04082884 | 0.0004655 | down |
| NOB1 | -0.613956478 | 9.427145211 | 0.000486953 | down |
| PRR12 | -0.652196833 | 9.229115658 | 0.000489679 | down |
| CCDC25 | -0.641884611 | 10.06090689 | 0.000509283 | down |
| PLEKHB1 | -0.673036229 | 2.775964305 | 0.000518584 | down |
| D4S234E | -2.863317041 | 3.963057458 | 0.000524199 | down |
| PARP16 | -0.658062067 | 10.16382979 | 0.000560905 | down |
| CCNB1IP1 | -0.7234732 | 9.521816868 | 0.000564144 | down |
| KDSR | -0.835734428 | 9.637696447 | 0.000574643 | down |
| RHPN2 | -0.780212278 | 1.506258803 | 0.000583124 | down |
| RPRD2 | -0.8021228 | 8.962529195 | 0.000588571 | down |
| PRPF6 | -0.641467926 | 3.135174142 | 0.000598472 | down |
| FCF1 | -0.659402756 | 9.468310526 | 0.000615124 | down |
| MRPL3 | -0.631654744 | 10.07149692 | 0.000620617 | down |
| BACH2 | -1.595945411 | 8.720225358 | 0.000623431 | down |
| TADA1 | -1.214374922 | 8.628581974 | 0.000635389 | down |
| ALKBH3 | -2.070596857 | 5.285059553 | 0.000637092 | down |
| SNX25 | -2.54580224 | 5.511688916 | 0.00065607 | down |
| FAM84B | -0.751220389 | 9.654924263 | 0.000664396 | down |
| SPINK2 | -0.773146382 | 1.448761086 | 0.000664931 | down |
| POLR1E | -2.440667078 | 7.854697042 | 0.000674681 | down |
| TTC27 | -0.776781289 | 8.318538868 | 0.000680557 | down |
| CD99L2 | -0.762401811 | 9.505424 | 0.000683222 | down |
| C1QBP | -0.628240322 | 9.531854579 | 0.000736921 | down |
| CRTAM | -0.871842733 | 9.933016263 | 0.000760994 | down |
| NSMCE1 | -0.680570389 | 10.53630555 | 0.000781019 | down |
| SAMM50 | -0.602175 | 9.604347211 | 0.000793522 | down |
| SLC39A8 | -0.812976411 | 8.675824611 | 0.000795474 | down |
| PIGK | -1.628528366 | 7.585228437 | 0.0007974 | down |
| PJA1 | -1.429749748 | 3.679000189 | 0.000802657 | down |
| DNAH7 | -0.737370856 | 1.015231728 | 0.00083272 | down |
| SSPN | -2.762281777 | 4.508921647 | 0.000850608 | down |
| ZBTB3 | -0.603583606 | 9.242282026 | 0.000853549 | down |
| BRWD1 | -0.609065106 | 9.043053626 | 0.000858252 | down |
| TTC9 | -2.515404072 | 7.314732358 | 0.00086907 | down |
| DMRTC1 | -0.603954313 | 2.185522558 | 0.000879022 | down |
| BTNL9 | -0.602808143 | 1.605153741 | 0.000885317 | down |
| RALGAPA1 | -0.604435 | 10.16228468 | 0.000888405 | down |
| TMEM203 | -0.748728161 | 9.000877042 | 0.000900742 | down |
| KLF12 | -0.650537561 | 10.66081358 | 0.000906808 | down |
| LRRC1 | -1.661059089 | 7.902800437 | 0.000907465 | down |
| STMN3 | -0.735479667 | 10.44213839 | 0.000922274 | down |
| FAM128A | -1.765660818 | 6.873665489 | 0.000964924 | down |
| C4orf46 | -0.627474583 | 8.968069447 | 0.000974902 | down |
| SAE1 | -0.588107456 | 9.448754184 | 0.000976173 | down |
| SIT1 | -0.607480422 | 9.778503316 | 0.001011717 | down |
| VMAC | -1.41295329 | 3.932057726 | 0.001019367 | down |
| NCALD | -1.149281844 | 9.539094237 | 0.001022854 | down |
| HOOK1 | -0.666050943 | 2.275230995 | 0.001025712 | down |
| EID2 | -0.633555149 | 3.041718189 | 0.001030096 | down |
| ZNF709 | -2.802760427 | 5.7051994 | 0.001053569 | down |
| FAM173B | -0.867002236 | 3.388201811 | 0.001070711 | down |
| OXCT1 | -1.606583211 | 7.5387253 | 0.001095977 | down |
| SPAG16 | -2.168940058 | 6.004194621 | 0.001148889 | down |
| MED9 | -0.676863523 | 2.877063026 | 0.00116651 | down |
| LCK | -0.649582167 | 10.61412018 | 0.001203974 | down |
| RIOK2 | -0.591173606 | 9.412481447 | 0.001221981 | down |
| ZFP1 | -2.101816422 | 7.002693747 | 0.001261693 | down |
| KIAA1737 | -0.740255433 | 8.727124789 | 0.001283186 | down |
| TWISTNB | -0.704869478 | 9.651512158 | 0.001323201 | down |
| NOSIP | -0.632989144 | 10.74177224 | 0.001348722 | down |
| AGAP1 | -0.59806131 | 1.992630711 | 0.001377884 | down |
| KLRG1 | -0.849429222 | 11.84566429 | 0.001405504 | down |
| PFN2 | -2.943773087 | 5.599966789 | 0.001406081 | down |
| GZMK | -0.715066761 | 12.51365082 | 0.001456967 | down |
| HSF2 | -0.8213874 | 9.487096458 | 0.001460003 | down |
| FEN1 | -2.148010032 | 5.897148374 | 0.001525505 | down |
| NDUFAF4 | -2.56105649 | 6.347259768 | 0.001548694 | down |
| ZNF286A | -2.739930211 | 5.846460921 | 0.001563296 | down |
| NT5E | -2.982322993 | 5.914177847 | 0.001600238 | down |
| LEO1 | -1.658162678 | 6.401226621 | 0.001619241 | down |
| NCRNA00219 | -0.659478083 | 10.47368139 | 0.001668538 | down |
| C18orf19 | -1.527761033 | 7.539251021 | 0.001694975 | down |
| RNF144A | -0.838185389 | 10.45676232 | 0.001723353 | down |
| FAM113B | -1.293250573 | 7.926679663 | 0.001723697 | down |
| METT10D | -0.669815883 | 8.895149342 | 0.001745858 | down |
| LOC728554 | -0.719087278 | 9.105819711 | 0.001780865 | down |
| DSC1 | -0.591025533 | 0.983259658 | 0.001784852 | down |
| LDLRAP1 | -0.875209433 | 9.917647911 | 0.001806599 | down |
| TKTL1 | -2.649294141 | 5.142160326 | 0.001814319 | down |
| AXIN2 | -2.059778948 | 5.436832605 | 0.00188 | down |
| THNSL1 | -2.265964938 | 5.808244147 | 0.001899407 | down |
| TMEM38B | -2.32967968 | 6.8311613 | 0.001902564 | down |
| SH3YL1 | -1.018389889 | 10.44130679 | 0.001905468 | down |
| CDR2 | -0.814702822 | 3.019048137 | 0.001906817 | down |
| MRPL45 | -0.614107861 | 9.360277053 | 0.001960881 | down |
| DPP4 | -1.820818917 | 8.246761279 | 0.001980322 | down |
| CASK | -0.725705606 | 8.817130763 | 0.001989624 | down |
| ZNF264 | -1.023564228 | 8.149244537 | 0.0020556 | down |
| DUSP16 | -1.333760933 | 8.612474605 | 0.002063818 | down |
| JMY | -0.685581656 | 9.722700895 | 0.002092298 | down |
| MLLT3 | -2.252979239 | 5.093081358 | 0.002110391 | down |
| ZNF441 | -1.805469648 | 4.917357705 | 0.002173739 | down |
| C1orf156 | -1.031672111 | 8.475633211 | 0.002200453 | down |
| PLEKHA5 | -1.897687056 | 3.296427705 | 0.002212544 | down |
| FAM134B | -0.916315556 | 9.674346989 | 0.00222808 | down |
| KLK1 | -2.668747986 | 3.684146047 | 0.002321357 | down |
| TMEM109 | -1.772185444 | 7.360437837 | 0.002389669 | down |
| SH2D1A | -0.796118506 | 10.21869918 | 0.002435355 | down |
| TDP1 | -1.630866813 | 7.156438221 | 0.00245167 | down |
| BCAS4 | -1.725763739 | 8.301320016 | 0.002465507 | down |
| ROBO1 | -0.902349664 | 1.785949742 | 0.00247475 | down |
| NRCAM | -1.574765198 | 2.096343071 | 0.002517166 | down |
| FLNB | -1.800792811 | 8.358832679 | 0.002572493 | down |
| KBTBD6 | -1.543091889 | 8.018277137 | 0.002624601 | down |
| RANBP2 | -1.914248681 | 7.129774295 | 0.00265405 | down |
| CD81 | -0.731415561 | 8.544055947 | 0.002663007 | down |
| MOCS2 | -0.629788822 | 9.029159079 | 0.002713686 | down |
| SIGLECP3 | -2.462791953 | 3.782886758 | 0.002728034 | down |
| PHAX | -1.310575161 | 7.705905789 | 0.002778027 | down |
| LOC100128551 | -2.022677546 | 3.867946774 | 0.002783216 | down |
| NBEA | -0.603349658 | 2.060059874 | 0.002852895 | down |
| ANKH | -0.76752395 | 10.80113347 | 0.002918338 | down |
| ZNF772 | -1.347073078 | 8.992956653 | 0.002929448 | down |
| TC2N | -0.960135511 | 9.758609368 | 0.002999455 | down |
| METTL2B | -1.123802164 | 7.592564779 | 0.003014919 | down |
| ITM2A | -0.625140061 | 11.22420647 | 0.003016662 | down |
| GINS3 | -2.196719364 | 5.884282542 | 0.003034527 | down |
| ZNF549 | -2.288672977 | 6.130629658 | 0.003041257 | down |
| TRA@ | -0.860517511 | 10.13478784 | 0.003044748 | down |
| HIBADH | -0.730279067 | 9.391028237 | 0.00309591 | down |
| ABCD2 | -0.596368357 | 2.605709116 | 0.003105027 | down |
| BHLHB9 | -0.689797867 | 2.584819442 | 0.003108513 | down |
| PRMT1 | -0.945950492 | 8.028788858 | 0.00313153 | down |
| ZZZ3 | -0.857596689 | 8.199251189 | 0.003268665 | down |
| ZNF543 | -0.657764826 | 3.001043858 | 0.003324907 | down |
| ZNF398 | -0.678335383 | 9.944108289 | 0.00334929 | down |
| UNG | -1.992124783 | 6.445309495 | 0.003424702 | down |
| ZNF671 | -1.562052364 | 7.543134779 | 0.003435014 | down |
| EOMES | -0.598016939 | 12.27447037 | 0.003448743 | down |
| CD8B | -2.588844322 | 9.182149421 | 0.003462526 | down |
| ZNF204P | -1.544561874 | 2.861280558 | 0.003516659 | down |
| KIF21A | -1.175410639 | 9.540247289 | 0.003541909 | down |
| ALDH18A1 | -1.611125989 | 7.912589358 | 0.003566297 | down |
| KCNA3 | -1.942763267 | 4.905068716 | 0.003649197 | down |
| FAM171A1 | -1.475355856 | 8.014511526 | 0.00369982 | down |
| SC5DL | -0.886475922 | 8.085551253 | 0.003718331 | down |
| AQR | -0.664139283 | 9.567607158 | 0.0037264 | down |
| THAP2 | -1.022859669 | 3.219695605 | 0.003732929 | down |
| SDCCAG8 | -0.612654933 | 8.936599632 | 0.003750933 | down |
| LPAR5 | -0.782757372 | 2.482682442 | 0.003760572 | down |
| GFM1 | -0.840798638 | 8.015407321 | 0.003761952 | down |
| PASK | -1.894160967 | 9.778620658 | 0.003771564 | down |
| C21orf45 | -2.072903771 | 5.945651774 | 0.003849493 | down |
| RWDD2B | -0.595230472 | 9.107340974 | 0.003870842 | down |
| ICOS | -1.697436277 | 7.283786347 | 0.003877298 | down |
| CD7 | -2.114391123 | 7.0342998 | 0.003898634 | down |
| GDPD1 | -0.749997274 | 2.777107463 | 0.003903546 | down |
| ZNF275 | -1.75661555 | 6.1368638 | 0.003939475 | down |
| CD5 | -0.853088711 | 9.439269947 | 0.003950805 | down |
| SLC12A2 | -0.686514794 | 8.826410832 | 0.004008607 | down |
| LRRC16A | -2.08128874 | 3.932013526 | 0.004013831 | down |
| WDR89 | -1.620589194 | 6.384681137 | 0.004050234 | down |
| MAGEF1 | -0.691282528 | 9.375136132 | 0.004083824 | down |
| C2orf89 | -1.174941867 | 9.140693489 | 0.004155622 | down |
| LOC220930 | -1.690539116 | 4.354782674 | 0.004202526 | down |
| AIP | -0.679402074 | 8.274427295 | 0.00428593 | down |
| NT5DC1 | -0.6336219 | 8.513903811 | 0.004298613 | down |
| CEP68 | -0.828934978 | 9.035643589 | 0.004306952 | down |
| CIAPIN1 | -0.760083611 | 8.170514595 | 0.004312022 | down |
| AKR1B1 | -0.661305789 | 9.162579789 | 0.00432389 | down |
| BDH2 | -0.765001828 | 9.286816263 | 0.004385706 | down |
| ID3 | -2.449672787 | 5.2997595 | 0.004407518 | down |
| SPRY1 | -0.589483839 | 2.152254323 | 0.004465577 | down |
| DBF4 | -0.810049911 | 8.599495047 | 0.004493387 | down |
| NUDCD2 | -1.710617207 | 6.739510879 | 0.004538365 | down |
| ICMT | -0.971108283 | 8.522664789 | 0.004936262 | down |
| ZNF569 | -0.630910412 | 2.203607889 | 0.004949846 | down |
| ZNF329 | -1.078146379 | 3.310118174 | 0.005025446 | down |
| PCGF6 | -1.476827544 | 3.704036811 | 0.005032818 | down |
| PRSS35 | -0.648954115 | 1.062631999 | 0.005056611 | down |
| DLAT | -0.847164506 | 7.9481243 | 0.005057515 | down |
| RSAD1 | -0.656445344 | 8.972330605 | 0.005057824 | down |
| CASD1 | -0.685958833 | 9.113599832 | 0.005100426 | down |
| MYC | -2.080525546 | 4.774246389 | 0.0051452 | down |
| TCEAL8 | -0.608451656 | 9.574618158 | 0.00518965 | down |
| BEX4 | -0.63498265 | 8.996160395 | 0.005199073 | down |
| TGFBR3 | -0.946083217 | 10.19901987 | 0.005205459 | down |
| USP37 | -0.594265644 | 9.561063947 | 0.00520701 | down |
| RAD51C | -0.608488122 | 9.294598684 | 0.00520837 | down |
| DCTPP1 | -0.8059878 | 8.495975905 | 0.005211055 | down |
| ASB1 | -0.756550372 | 9.899175884 | 0.005391801 | down |
| MRPL39 | -0.634551011 | 8.825600105 | 0.005453275 | down |
| PTCH1 | -0.990822689 | 10.56510224 | 0.005494749 | down |
| CCR6 | -0.683228156 | 10.79490121 | 0.005542105 | down |
| REG4 | -0.778208829 | 1.522416601 | 0.00568811 | down |
| INPP4B | -1.863859112 | 7.202541558 | 0.00575815 | down |
| PDCD7 | -0.740031056 | 8.711077174 | 0.005870794 | down |
| GLB1L2 | -0.631313238 | 1.365377317 | 0.005901431 | down |
| ZNF181 | -1.485088207 | 7.455447605 | 0.005963762 | down |
| EPHX2 | -1.113041717 | 9.120088363 | 0.005998797 | down |
| PHB | -1.902085822 | 6.841278779 | 0.006011425 | down |
| EIF3C | -0.601128144 | 10.55189679 | 0.006131033 | down |
| HMGA1 | -0.608532967 | 11.03577784 | 0.006246077 | down |
| PIGU | -0.954467857 | 3.539784137 | 0.006267623 | down |
| FAM83H | -1.787083758 | 4.243452221 | 0.00627825 | down |
| GPM6A | -0.629446543 | 1.196948301 | 0.006386026 | down |
| TRIM59 | -0.904595628 | 8.033773984 | 0.006415197 | down |
| SDC2 | -0.683684167 | 2.245488705 | 0.006427681 | down |
| FIGNL1 | -0.720757222 | 9.045613084 | 0.006442324 | down |
| RPL35A | -1.135897583 | 9.8595535 | 0.00645415 | down |
| HPS4 | -1.624557578 | 7.579959326 | 0.006480221 | down |
| NFX1 | -0.595067689 | 8.932671132 | 0.006525478 | down |
| ZNF3 | -0.633321133 | 9.132670579 | 0.006597363 | down |
| C2orf40 | -0.681660122 | 2.073263763 | 0.006639566 | down |
| PDGFD | -0.691635633 | 2.443391121 | 0.006667206 | down |
| CD1C | -2.197275072 | 8.029601742 | 0.006671976 | down |
| TAF9B | -0.728377089 | 9.041272974 | 0.006704752 | down |
| XPNPEP3 | -0.659638406 | 8.970360853 | 0.006769338 | down |
| ELAVL1 | -0.6613614 | 8.214007847 | 0.007048382 | down |
| RRP15 | -1.317864533 | 8.024326679 | 0.007153116 | down |
| PPAT | -0.737292089 | 10.88919808 | 0.007172956 | down |
| NAP1L3 | -1.077105037 | 2.837939521 | 0.007173638 | down |
| ZNF814 | -1.969514169 | 5.012808816 | 0.007222559 | down |
| ZFP64 | -1.425428133 | 7.8631729 | 0.007330922 | down |
| DDHD2 | -0.662182511 | 10.311044 | 0.007340023 | down |
| GRLF1 | -0.622509644 | 9.736326211 | 0.007397837 | down |
| KIAA1715 | -0.881597689 | 10.24516989 | 0.007445378 | down |
| SNORD72 | -1.098037789 | 8.509023737 | 0.007569683 | down |
| TRAPPC2 | -1.998323651 | 7.205686779 | 0.007612279 | down |
| USP44 | -2.375800446 | 4.292861732 | 0.007905278 | down |
| ORMDL3 | -0.68881455 | 9.388317274 | 0.007938162 | down |
| SHMT1 | -2.572179696 | 5.671497132 | 0.007967544 | down |
| EIF2AK3 | -0.603975539 | 8.670422574 | 0.008003494 | down |
| COBLL1 | -2.539270613 | 7.122983284 | 0.008108123 | down |
| AUTS2 | -1.055831594 | 9.933807395 | 0.008204652 | down |
| CYTH3 | -1.918722779 | 3.904822605 | 0.008266162 | down |
| LY9 | -1.680534831 | 7.601277005 | 0.008299744 | down |
| ZNF566 | -1.582477467 | 8.019422311 | 0.008324578 | down |
| SLC16A1 | -0.671493906 | 8.997399789 | 0.00841572 | down |
| TXK | -0.661868294 | 10.10602929 | 0.008425575 | down |
| TMIGD2 | -1.204229073 | 3.177657842 | 0.008426974 | down |
| ATP6V0E2 | -1.151803748 | 3.712152921 | 0.008557817 | down |
| PAICS | -1.562135704 | 7.455883142 | 0.008610087 | down |
| SGCB | -0.828741519 | 2.877887358 | 0.00866908 | down |
| PRPSAP1 | -1.757623664 | 7.497529442 | 0.008851644 | down |
| SNPH | -0.70682482 | 2.123376413 | 0.008854415 | down |
| VEGFB | -1.938405804 | 6.370811537 | 0.008916502 | down |
| CAPN7 | -0.735246544 | 8.260652021 | 0.008928816 | down |
| ZNF805 | -0.605675467 | 9.819577395 | 0.009167186 | down |
| ZNF561 | -1.339323794 | 7.734638516 | 0.009325242 | down |
| C7orf29 | -0.657845253 | 2.792563111 | 0.009461168 | down |
| AMAC1 | -0.723934542 | 2.150628558 | 0.009784476 | down |
| ALG8 | -0.625724453 | 8.041186726 | 0.010000046 | down |
| ZNF382 | -1.827796154 | 6.761937832 | 0.010056045 | down |
| MAPK9 | -0.894892806 | 8.613441458 | 0.01009046 | down |
| ADAT2 | -1.38776201 | 3.447639358 | 0.010300726 | down |
| CTGF | -0.628253601 | 1.243777226 | 0.01033041 | down |
| ZFP90 | -0.7378649 | 9.455906647 | 0.010608632 | down |
| PEG10 | -0.595305976 | 1.774070439 | 0.010856966 | down |
| TMEM204 | -1.266498376 | 2.542622537 | 0.010903204 | down |
| SUCLG2 | -0.672205787 | 7.937966353 | 0.010985595 | down |
| C1orf57 | -2.105176333 | 6.201301711 | 0.011062222 | down |
| TSPAN13 | -0.791265361 | 11.39916034 | 0.011264187 | down |
| WRN | -1.47734295 | 7.328472321 | 0.011308579 | down |
| COCH | -0.672443041 | 2.169827979 | 0.011317784 | down |
| SNRPN | -0.617129711 | 9.753367 | 0.011341444 | down |
| CDKN2AIP | -1.24907208 | 6.475805758 | 0.011451428 | down |
| NHEDC2 | -1.836083641 | 5.7903109 | 0.011628388 | down |
| NOP58 | -0.880857322 | 8.577889895 | 0.011647663 | down |
| IFT57 | -1.403563706 | 8.475910384 | 0.011656605 | down |
| DEXI | -1.484597698 | 7.163194332 | 0.011685409 | down |
| HN1L | -1.411564839 | 7.923747374 | 0.011812413 | down |
| PID1 | -0.932732122 | 9.995443421 | 0.01196033 | down |
| FAM162A | -1.200254967 | 8.195131874 | 0.012038151 | down |
| FANCE | -1.419945334 | 7.258195705 | 0.012168301 | down |
| OSBPL10 | -2.565221454 | 6.252127511 | 0.012175733 | down |
| PHLDA1 | -1.8519516 | 4.369659468 | 0.012181738 | down |
| PVRIG | -1.981855567 | 7.275332811 | 0.012539182 | down |
| ZNF831 | -1.984395461 | 6.190794353 | 0.012593728 | down |
| CCDC65 | -1.553900406 | 8.517436463 | 0.012940336 | down |
| KEAP1 | -1.041840508 | 6.760380632 | 0.013076237 | down |
| HAUS5 | -0.67691815 | 9.098292611 | 0.013179531 | down |
| GPRASP1 | -1.895273944 | 7.866132758 | 0.013290267 | down |
| SFXN2 | -1.861614031 | 5.158303147 | 0.013389927 | down |
| POLD2 | -1.670364408 | 7.089330284 | 0.013416052 | down |
| FBLN5 | -2.224862978 | 4.256515937 | 0.013539873 | down |
| ZNF823 | -1.245178082 | 3.485150263 | 0.013607254 | down |
| SNHG8 | -1.001744378 | 8.814464705 | 0.013631069 | down |
| MANEAL | -1.01241931 | 3.034779668 | 0.013643398 | down |
| ENOX2 | -1.014461348 | 7.348160647 | 0.013732312 | down |
| TXLNG | -1.146125333 | 7.957412726 | 0.013754719 | down |
| KBTBD8 | -1.538244597 | 7.129147353 | 0.01385251 | down |
| C2orf44 | -0.678282378 | 8.695870947 | 0.014417012 | down |
| UFSP2 | -1.162584994 | 7.872200395 | 0.014475879 | down |
| GPATCH4 | -1.680798296 | 5.959788226 | 0.014567393 | down |
| RANBP1 | -0.946290633 | 7.654129468 | 0.014653202 | down |
| IPO11 | -1.820282479 | 6.874506821 | 0.014880703 | down |
| PITPNC1 | -0.737174994 | 10.49627889 | 0.01490857 | down |
| TSPAN3 | -1.805670044 | 6.877707311 | 0.015144384 | down |
| ZMAT3 | -2.163886544 | 9.599465011 | 0.015232996 | down |
| ALKBH8 | -0.860369333 | 7.833979932 | 0.015262505 | down |
| GPR68 | -2.319027334 | 6.596132158 | 0.015480191 | down |
| GPX7 | -2.003636851 | 4.5663756 | 0.015604633 | down |
| KIF3A | -0.663838111 | 10.19823837 | 0.015761966 | down |
| MGMT | -1.3112259 | 7.669408795 | 0.015782743 | down |
| TYW1 | -1.943980576 | 5.098255916 | 0.015863609 | down |
| ZNF573 | -1.559491278 | 8.115282205 | 0.015939784 | down |
| RBM15B | -0.632069656 | 9.158985484 | 0.016046191 | down |
| ZNF557 | -1.037903581 | 8.206352932 | 0.016096807 | down |
| PELP1 | -1.833877318 | 5.974542711 | 0.016275178 | down |
| MYBBP1A | -0.91347966 | 7.561913968 | 0.016606993 | down |
| CDKN2AIPNL | -1.468124317 | 6.520903942 | 0.016838307 | down |
| GATA3 | -1.525041983 | 9.037291542 | 0.017188967 | down |
| TRAF3IP1 | -0.86211255 | 8.517080247 | 0.017307274 | down |
| UNC119B | -0.749766444 | 9.189603274 | 0.017550098 | down |
| GMEB1 | -0.688138074 | 3.190395489 | 0.017700835 | down |
| ALG9 | -0.691132517 | 8.035118489 | 0.01772748 | down |
| BHLHE41 | -0.605014066 | 1.9133077 | 0.017769325 | down |
| GIT1 | -0.605152689 | 9.205443026 | 0.017858203 | down |
| ORAI2 | -1.287457589 | 8.447392068 | 0.01799365 | down |
| YES1 | -2.110204368 | 5.685112 | 0.018040727 | down |
| BTBD11 | -2.235901206 | 6.402436553 | 0.018690815 | down |
| ZNF91 | -0.727201256 | 10.09135695 | 0.018848925 | down |
| ZNF26 | -1.029675701 | 7.872034168 | 0.018898247 | down |
| UTP15 | -1.427925353 | 5.471642426 | 0.019042214 | down |
| HOPX | -0.784550789 | 10.01514908 | 0.019142885 | down |
| COQ3 | -0.87821199 | 3.477580437 | 0.019177001 | down |
| DIS3L | -1.361717076 | 7.673746242 | 0.019320419 | down |
| MRPL2 | -0.662136461 | 9.064737663 | 0.019435344 | down |
| CXXC5 | -1.470450694 | 8.835337374 | 0.019608942 | down |
| DNAJA3 | -1.389732738 | 7.303213005 | 0.019769667 | down |
| ROR2 | -0.845401632 | 2.039014705 | 0.020298307 | down |
| SCD5 | -2.511695361 | 6.099772616 | 0.020373178 | down |
| LMBR1 | -0.859667189 | 8.391030626 | 0.020520834 | down |
| ACN9 | -1.506512466 | 7.362956242 | 0.020529432 | down |
| MTERFD2 | -1.273573474 | 7.611991484 | 0.020534977 | down |
| TAF3 | -1.614831288 | 7.222435026 | 0.020569651 | down |
| CLPP | -1.446124996 | 6.960201763 | 0.020583399 | down |
| ZNF649 | -1.015715183 | 3.140137305 | 0.020653593 | down |
| HLA-DOA | -1.513321589 | 9.837416758 | 0.020750219 | down |
| TMEM42 | -1.332649601 | 7.164968916 | 0.020942642 | down |
| COX11 | -0.726889994 | 8.828194237 | 0.020990479 | down |
| RAD54B | -1.447002974 | 3.713414268 | 0.021158829 | down |
| NHEDC1 | -1.098162838 | 2.193126463 | 0.021262235 | down |
| NIPSNAP1 | -1.738675009 | 5.746235721 | 0.02128907 | down |
| AFF3 | -1.6730194 | 9.341193026 | 0.021477703 | down |
| ZNF84 | -0.785194986 | 8.265680863 | 0.021511366 | down |
| TSGA14 | -1.683845419 | 5.510660942 | 0.021654467 | down |
| ZNF530 | -0.609591814 | 2.536750353 | 0.021666426 | down |
| RECK | -1.124789844 | 8.274126911 | 0.021717222 | down |
| KIF20B | -1.374582506 | 7.836720421 | 0.021758723 | down |
| ZNF184 | -1.597378899 | 4.070709647 | 0.021805133 | down |
| HYLS1 | -1.134091142 | 6.947724853 | 0.021863797 | down |
| WDR54 | -1.351402667 | 8.966142595 | 0.022252863 | down |
| SLC3A1 | -0.785269053 | 3.239265868 | 0.022351243 | down |
| TGFB3 | -1.042046299 | 2.680416632 | 0.022435218 | down |
| ACPL2 | -0.965789544 | 8.273098242 | 0.022442838 | down |
| LIN54 | -0.782555319 | 4.300515232 | 0.022468307 | down |
| LIMA1 | -1.60344705 | 7.5709018 | 0.02268389 | down |
| XCL2 | -0.726103772 | 10.38994266 | 0.022698458 | down |
| TASP1 | -1.516588116 | 6.208432616 | 0.022710219 | down |
| MAGEH1 | -1.398381057 | 4.565355563 | 0.022960995 | down |
| FDX1 | -1.829697267 | 5.338795095 | 0.023176565 | down |
| MTCP1NB | -1.33963229 | 7.496502826 | 0.023374704 | down |
| C12orf45 | -1.871276791 | 6.191886711 | 0.023590976 | down |
| TECR | -1.311331452 | 7.763287526 | 0.02371375 | down |
| ABHD15 | -1.131754943 | 3.538434963 | 0.02380512 | down |
| ABL1 | -1.847790616 | 5.600847521 | 0.023955635 | down |
| FAM19A1 | -1.089186997 | 2.672978947 | 0.024108735 | down |
| ZNF227 | -1.091972733 | 7.328372858 | 0.024113553 | down |
| TPD52 | -0.679404333 | 9.999038895 | 0.024235135 | down |
| ZNF721 | -0.611008411 | 9.511237263 | 0.024261565 | down |
| LOC284889 | -1.165513352 | 3.801185158 | 0.024426327 | down |
| TECR | -1.331635333 | 7.631773768 | 0.024785422 | down |
| TOX | -1.0263169 | 8.720349889 | 0.024844076 | down |
| AK5 | -0.72432099 | 2.740626874 | 0.024866971 | down |
| MCM6 | -1.665959812 | 6.062363932 | 0.0248924 | down |
| CTLA4 | -0.625457033 | 9.382375079 | 0.024924167 | down |
| HS3ST3B1 | -0.680808633 | 9.1095167 | 0.025284691 | down |
| PIGP | -0.705228183 | 9.095046395 | 0.025297183 | down |
| GDF11 | -1.039372958 | 7.482637121 | 0.025337089 | down |
| ZNF544 | -1.310134422 | 8.562313011 | 0.025393413 | down |
| STRBP | -1.331070872 | 7.954189495 | 0.025978559 | down |
| ZSCAN18 | -1.220864578 | 3.030888426 | 0.025992667 | down |
| UBAP2 | -0.997505867 | 8.114520321 | 0.026061627 | down |
| ZFAND1 | -0.6625647 | 9.234629463 | 0.027362464 | down |
| LMO7 | -1.772109418 | 4.944716889 | 0.027431683 | down |
| TNRC6C | -1.395884222 | 8.991266584 | 0.027638749 | down |
| ZNF284 | -1.375225978 | 8.955460768 | 0.02810186 | down |
| PDRG1 | -1.315887059 | 6.388011647 | 0.028103588 | down |
| BRMS1L | -1.520729284 | 6.624544289 | 0.028159813 | down |
| TFB2M | -1.651835168 | 7.427444653 | 0.028296908 | down |
| CACHD1 | -0.693539828 | 2.349819484 | 0.028387488 | down |
| RRM1 | -1.771903597 | 6.107174374 | 0.028548802 | down |
| CAMK2N1 | -2.182864142 | 6.076873042 | 0.02861635 | down |
| THEM4 | -0.967755378 | 9.088211058 | 0.028795015 | down |
| TMEM9 | -0.8976879 | 8.845925316 | 0.029737146 | down |
| LOC339803 | -0.605517544 | 2.3933626 | 0.02977371 | down |
| GPD1L | -0.958388389 | 8.957464553 | 0.029850741 | down |
| DNAH11 | -0.774188083 | 9.963492079 | 0.030235765 | down |
| AATF | -0.674823211 | 7.7922352 | 0.030272594 | down |
| ZCCHC11 | -1.682859434 | 5.703500347 | 0.030443992 | down |
| PLXDC1 | -1.215264169 | 3.433887332 | 0.030504153 | down |
| INO80 | -1.556298923 | 6.595894463 | 0.030619176 | down |
| KCTD2 | -1.256096342 | 7.337308116 | 0.03090855 | down |
| PTPRM | -2.182035931 | 5.262840553 | 0.031003389 | down |
| PHF14 | -1.178685277 | 7.378550905 | 0.031134858 | down |
| UMPS | -0.864512489 | 8.529713084 | 0.031339084 | down |
| PRIM1 | -1.501383371 | 4.175784863 | 0.03142371 | down |
| UGDH | -0.883143051 | 8.043690521 | 0.031688612 | down |
| MRPS17 | -1.566407102 | 5.966796768 | 0.031941636 | down |
| TRUB2 | -1.332754034 | 6.242282547 | 0.032215685 | down |
| EEF1E1 | -1.159469907 | 7.547066453 | 0.032457845 | down |
| TSR1 | -1.442669709 | 6.432085758 | 0.032816843 | down |
| MKL2 | -1.434883417 | 8.168422032 | 0.032861943 | down |
| PKIG | -2.168505366 | 5.187891858 | 0.032944067 | down |
| POLR3D | -1.529053988 | 4.315929979 | 0.033282512 | down |
| ZMAT4 | -0.658763398 | 2.0730767 | 0.033581989 | down |
| KIAA0564 | -1.459857274 | 7.697711789 | 0.033699053 | down |
| NBPF1 | -1.178247244 | 8.775935484 | 0.033856953 | down |
| PTPRCAP | -1.229196106 | 8.566578263 | 0.034214684 | down |
| WBP11 | -0.613771211 | 8.335071768 | 0.034502339 | down |
| SNRPN | -1.679870558 | 4.682043821 | 0.034760928 | down |
| ZNF134 | -0.946928226 | 3.161906921 | 0.034930292 | down |
| TMTC3 | -1.120019933 | 6.485408842 | 0.034984871 | down |
| ZNF295 | -0.765854789 | 8.242579979 | 0.035069454 | down |
| CD40LG | -0.883667224 | 3.249600568 | 0.035164924 | down |
| TRIM37 | -1.459171556 | 4.439871574 | 0.035253926 | down |
| CLIC5 | -1.288624432 | 3.456175363 | 0.035256081 | down |
| ORC5L | -1.2897396 | 7.117137616 | 0.035540419 | down |
| FLVCR1 | -0.983527567 | 8.722769421 | 0.035558229 | down |
| ZSCAN2 | -1.022602122 | 8.513374421 | 0.035647629 | down |
| POU6F1 | -1.670994756 | 8.107948368 | 0.036226633 | down |
| CD247 | -0.697151344 | 9.351804916 | 0.037051068 | down |
| TSEN2 | -1.51568923 | 4.508512474 | 0.037328029 | down |
| NAA10 | -0.633922961 | 8.557878253 | 0.03815856 | down |
| FGFBP3 | -0.89846745 | 3.010458105 | 0.038227669 | down |
| MNAT1 | -0.769856141 | 2.787467932 | 0.038649125 | down |
| MSH3 | -0.745101332 | 3.092725274 | 0.0394908 | down |
| SLC25A15 | -1.306914648 | 6.632835537 | 0.03954098 | down |
| HADH | -1.005922211 | 8.604115532 | 0.039666198 | down |
| AKT3 | -0.625574144 | 9.320967105 | 0.040192594 | down |
| TEX261 | -0.867238262 | 7.725836168 | 0.040205049 | down |
| SYNJ2 | -1.466186517 | 5.234308047 | 0.040217475 | down |
| PRKCA | -1.005317889 | 8.902937189 | 0.040238839 | down |
| SMARCB1 | -0.893036716 | 7.767220268 | 0.040707077 | down |
| AEBP2 | -0.703752661 | 8.494899526 | 0.041022988 | down |
| ORAI1 | -1.228160627 | 6.221014363 | 0.04115272 | down |
| RALGPS2 | -0.950879606 | 8.542833795 | 0.041451103 | down |
| C12orf65 | -1.177903179 | 5.246090153 | 0.042191222 | down |
| TXLNA | -0.905885669 | 6.535604742 | 0.04228423 | down |
| MTPAP | -1.395470783 | 7.572707174 | 0.042414986 | down |
| KANK3 | -1.502989742 | 5.300618111 | 0.042657777 | down |
| N4BP2 | -1.029084403 | 7.560485337 | 0.042981046 | down |
| ALMS1 | -1.329724977 | 3.934521584 | 0.043052256 | down |
| RBAK | -0.737604094 | 3.220101537 | 0.043235207 | down |
| ABI2 | -1.176115022 | 8.479075084 | 0.043352437 | down |
| C3orf45 | -1.311822484 | 5.750141442 | 0.0435159 | down |
| CBX7 | -0.638313772 | 10.43622739 | 0.043671427 | down |
| ZMYM3 | -0.598197322 | 8.583316974 | 0.043707029 | down |
| BTLA | -1.313200652 | 8.483912516 | 0.043815089 | down |
| C1orf107 | -0.860381624 | 3.399066189 | 0.04394323 | down |
| CAMK1D | -0.730866289 | 10.01650908 | 0.044077483 | down |
| ZNF43 | -1.578246813 | 5.671045184 | 0.044609266 | down |
| BCL9L | -0.988439 | 8.031412453 | 0.044639101 | down |
| ZXDB | -1.173825511 | 7.930022689 | 0.044699285 | down |
| PLEKHG1 | -2.04044144 | 5.193656247 | 0.045054353 | down |
| DSTYK | -0.706824917 | 8.336129547 | 0.045279155 | down |
| TSPYL5 | -1.271846297 | 3.184682921 | 0.045293894 | down |
| ELOVL4 | -1.112183174 | 2.767796321 | 0.045295819 | down |
| BOLA1 | -0.915604331 | 3.359109505 | 0.045413571 | down |
| GLT25D2 | -1.989349192 | 4.506179668 | 0.045580618 | down |
| ZNF365 | -1.158048808 | 2.221019895 | 0.045708844 | down |
| SLC25A42 | -1.305136936 | 7.333661921 | 0.045809355 | down |
| RBBP9 | -1.329190859 | 4.765455647 | 0.046174151 | down |
| DLG3 | -0.938544467 | 8.727643605 | 0.046200772 | down |
| BCKDHB | -1.200698139 | 8.6590605 | 0.046322346 | down |
| RPP38 | -1.174705612 | 6.689785089 | 0.04636504 | down |
| LZTFL1 | -1.008829872 | 8.873897668 | 0.047019248 | down |
| ARHGAP5 | -1.553194887 | 4.740211626 | 0.047064917 | down |
| USP11 | -1.40987378 | 6.588666432 | 0.04709686 | down |
| CSRP2BP | -1.549626408 | 6.549039968 | 0.047536753 | down |
| ISOC1 | -1.037969623 | 7.751963168 | 0.047893047 | down |
| EPPK1 | -1.833534551 | 7.994976674 | 0.04803128 | down |
| CDCA7L | -0.597886428 | 10.71798379 | 0.048597431 | down |
| TSPYL2 | -1.003501578 | 8.489155047 | 0.048796443 | down |
| THRA | -0.901660707 | 3.6011538 | 0.048850126 | down |
| S100B | -2.462397733 | 4.069247444 | 0.04922326 | down |
