# Supplementary material for: Mechanisms of Ganweikang Tablets against Chronic Hepatitis B: A Comprehensive Study of Network Analysis, Molecular Docking, and Chemical Profiling
Source: Biomed Res Int. 2023 May 8;2023:8782892. doi: 10.1155/2023/8782892 (PMC10185428; doi:10.1155/2023/8782892)
Supplement: Supplementary Materials — Figure S1: schematic diagrams for the binding modes between targets and positive control small molecules. Figure S2: the binding patterns between active ingredients, positive control, and targets. Table S1: DEG results and disease-related targets. Table S2: compound-related targets. Table S3: KEGG pathway enrichment results on each module in TPT network. Table S4: detail information of CTP network. Table S5: molecular docking results of key targets. Table S6: detail information of UPLC-QTOF/MS analysis. Table S7: detail information of GC/MS analysis. Table S8: detail information of key active ingredients. Table S9: detail information of key targets. [file 8782892.f1.zip › Supplementary table S2.docx]

| **Herb No.** | **PUBID** | **Molecular formular** | **MW** | **Canonical SMILES** | **Name** |
| --- | --- | --- | --- | --- | --- |
| HQ1 | 70719 | C6H7NO | 109.14 | CC1=C(C=CC=N1)O | 3-Hydroxy-2-picoline |
| HQ2 | 5359024 | C17H14O6 | 314.31 | COC1=C(C(=C(C=C1)C2=COC3=C(C2=O)C=CC(=C3)O)O)OC | 7-hydroxy-3-(2-hydroxy-3,4-dimethoxy-phenyl)chromone |
| HQ3 | 15689652 | C18H20O5 | 316.38 | COC1=CC2=C(CC(CO2)C3=C(C(=C(C=C3)OC)OC)O)C=C1 | 7-O-methylisomucronulatol |
| HQ4 | 736186 | C10H10O4 | 194.2 | COC1=C(C=C(C=C1)/C=C/C(=O)O)O | isoferulic acid |
| HQ5 | 108213 | C20H18O10 | 418.38 | COC1=C2C(=C(C(=C1)C(=O)OC)C3=C4C(=C(C=C3C(=O)OC)OC)OCO4)OCO2 | Bifendate |
| HQ6 | 1548883 | C10H10O4 | 194.2 | COC1=C(C=CC(=C1)/C=C\\C(=O)O)O | FERULIC ACID (CIS) |
| HQ7 | 5281708 | C15H10O4 | 254.25 | C1=CC(=CC=C1C2=COC3=C(C2=O)C=CC(=C3)O)O | daidzein |
| HQ8 | 938 | C6H5NO2 | 123.12 | C1=CC(=CN=C1)C(=O)O | nicotinic acid |
| HQ9 | 28782 | C6H14N4O2 | 174.24 | C(CC(C(=O)[O-])N)C[NH+]=C(N)N | Protonated arginine |
| HQ10 | 44257336 | C17H14O6 | 314.29 | COC1=C(C=C(C=C1)C2=COC3=CC(=CC(=C3C2=O)O)OC)O | 5,3'-Dihydroxy-7,4'-dimethoxyisoflavone |
| HQ11 | 5318279 | C22H24N2O4 | 380.4 | COC1=C(C=C2C3C4C(=CCC(=O)N4C2=C1)C5CC36CCN6CC5=CCO)O | 2-hydroxy-3-methoxystrychnine |
| HQ12 | 5316874 | C16H16O2 | 240.3 | COC1=CC(=CC(=C1)C=CC2=CC=CC=C2)OC | 3,5-dimethoxystilbene |
| HQ13 | 5318203 | C26H36O4 | 412.6 | CC1CC(=O)C2=C(O1)C(=C(C(C2=O)(C)CC=C(C)CCC=C(C)C)O)CC=C(C)C | 4-hydroxy-2,6-dimethyl-6-(3,7-dimethyl-2,6-octadienyl)-8-(3-methyl-2-butenyl)-2h-1-benzopyran-5,7(3h,6h)-dione |
| HQ14 | 29927927 | C9H10O4 | 182.17 | COC1=CC=C(CC1)C(=O)C(=O)O | 2-(4-Methoxycyclohexa-1,3-dien-1-yl)-2-oxoacetic acid |
| HQ15 | 5306422 | C14H14N4O5 | 318.28 | CN1C(=CC(=N1)[N+](=O)[O-])C(=O)NCC2=CC=C(C=C2)C(=O)OC | Methyl 4-({[(1-methyl-3-nitro-1H-pyrazol-5-yl)carbonyl]amino}methyl)benzoate |
| HQ16 | 25243900 | C5H13N4O3+ | 177.18 | C(CO[NH+]=C(N)N)C(C(=O)[O-])[NH3+] | canavanine |
| HQ17 | 3 | C7H8O4 | 156.14 | C1=CC(C(C(=C1)C(=O)O)O)O | isoastragaloside1, 3 |
| HQ18 | 5317378 | C13H10N2O2 | 226.23 | COC1=CN=C(C2=C1C3=CC=CC=C3N2)C=O | kumugansine a |
| HQ19 | 6267 | C4H8N2O3 | 132.12 | C(C(C(=O)O)N)C(=O)N | Asparagine |
| HQ20 | 119 | C4H9NO2 | 103.12 | C(CC(=O)O)CN | gamma-Aminobutyric acid |
| HQ21 | 145742 | C5H9NO2 | 115.13 | C1CC(NC1)C(=O)O | Proline |
| HQ22 | 6322 | C6H14N4O2 | 174.2 | C(CC(C(=O)O)N)CN=C(N)N | arginine |
| MBC1 | 18676629 | C2H2O4 | 90.04 | [H+].[H+].C(=O)(C(=O)[O-])[O-] | OXL |
| MBC2 | 521334 | C15H24 | 204.39 | CC(=C1CCC2(CCCC(=C)C2C1)C)C | gamma-selinene |
| MBC3 | 12444745 | C11H14O5 | 226.25 | CC1CC(=O)C2C1C(OC=C2C(=O)OC)O | cornin_qt |
| MBC4 | 73193 | C30H48O5 | 488.78 | CC1CCC2(CCC3(C(=CCC4C3(CCC5C4(CC(C(C5(C)C)O)O)C)C)C2C1(C)O)C)C(=O)O | Tormentic acid |
| MBC5 | 163040 | C9H12O4 | 184.21 | C1=COC(C2C1C(C=C2CO)O)O | aucubigenin |
| MBC6 | 12391 | C15H32 | 212.47 | CCCCCCCCCCCCCCC | MYS |
| MBC7 | 3032853 | C15H26 | 206.37 | CC1CCC(C2C1C=CC(C2)C)C(C)C | cadinene |
| MBC8 | 3036251 | C29H52O3 | 448.7 | CCC(CCC(C)C1CCC2C1(CCC3C2CC(C4(C3(CCC(C4)O)C)O)O)C)C(C)C | Stigmastane-3beta,5alpha,6beta-triol |
| MBC9 | 5320351 | C20H20O8 | 388.4 | COC1=C(C=C(C=C1)C2=C(C(=O)C3=C(C(=C(C=C3O2)OC)OC)O)OC)OC | artemisetin |
| MBC10 | 88298 | C10H16O | 152.23 | CC1=CC(C2CC1C2(C)C)O | verbenol |
| LQ1 | 6989 | C10H14O | 150.24 | CC1=CC(=C(C=C1)C(C)C)O | thymol |
| LQ2 | 3663 | C30H16O8 | 504.46 | CC1=CC(=O)C2=C(C3=C(C=C(C4=C3C5=C2C1=C6C(=CC(=O)C7=C(C8=C(C=C(C4=C8C5=C67)O)O)O)C)O)O)O | hypericin |
| LQ3 | 384877 | C22H26O6 | 386.48 | COC1=C(C=C(C=C1)CC2COC(=O)C2CC3=CC(=C(C=C3)OC)OC)OC | (3R,4R)-3,4-bis[(3,4-dimethoxyphenyl)methyl]oxolan-2-one |
| LQ4 | 12410 | C31H64 | 436.95 | CCCCCCCCCCCCCCCCCCCCCCCCCCCCCCC | Hentriacontan |
| LQ5 | 591119 | C10H10O3 | 178.2 | CCC1C2=C(C(=CC=C2)O)C(=O)O1 | 3-ethyl-7hydroxyphthalide |
| LQ6 | 184824 | C8H14O3 | 158.22 | C1CC(CCC1=O)(CCO)O | 4-hydroxy-4-(2-hydroxyethyl)cyclohexan-1-one |
| LQ7 | 345510 | C32H52O2 | 468.84 | CC(=O)OC1CCC2(C(C1(C)C)CCC3(C2CC=C4C3(CCC5(C4CC(CC5)(C)C)C)C)C)C | ß-amyrin acetate |
| LQ8 | 4978 | C30H16O9 | 520.46 | CC1=CC(=O)C2=C(C3=C(C=C(C4=C3C5=C2C1=C6C(=CC(=O)C7=C(C8=C(C=C(C4=C8C5=C67)O)O)O)CO)O)O)O | Psuedohypericin |
| LQ9 | 171335 | C30H18O9 | 522.48 | CC1=CC(=O)C2=C(C3=C(C=C(C4=C3C(=C5C6=CC(=CC(=O)C6=C(C7=C(C=C(C4=C57)O)O)O)CO)C2=C1)O)O)O | Protopseudohypericin |
| LQ10 | 164660 | C30H18O8 | 506.48 | CC1=CC(=O)C2=C(C3=C(C=C(C4=C3C(=C5C6=CC(=CC(=O)C6=C(C7=C(C=C(C4=C57)O)O)O)C)C2=C1)O)O)O | Protohypericin |
| LQ11 | 122635 | C15H12O4 | 256.27 | CC1=CC2=C(C(=C1)O)C(=O)C3=C(C2)C=C(C=C3O)O | Emodinanthrone |
| LQ12 | 5281631 | C13H8O4 | 228.21 | C1=CC(=C2C(=C1)OC3=C(C2=O)C=C(C=C3)O)O | Euxanthone |
| LQ13 | 10975 | C10H18O | 154.28 | CC(C)(CCCC(=C)C=C)O | Myrcenol |
| LQ14 | 231114 | C14H12O3 | 228.26 | CC(=CC1=C(C2=CC=CC=C2C(=O)C1=O)O)C | Norlapachol |
| LQ15 | 21952380 | C4H6O4 | 118.1 | [H+].[H+].C(CC(=O)[O-])C(=O)[O-] | succinic acid |
| LQ16 | 1549111 | C9H8O4 | 180.17 | C1=CC(=C(C=C1/C=C\\C(=O)O)O)O | Caffeate |
| LQ17 | 7462 | C10H16 | 136.26 | CC1=CC=C(CC1)C(C)C | Terpilene |
| LQ18 | 10057860 | C10H12O2 | 164.2 | CC1=CC(=O)C2C(C1=O)C2(C)C | (+-)-car-3-ene-2,5-dione |
| LQ19 | 8748 | C10H18O | 154.25 | CC(=C)C1CCC(CC1)(C)O | cis-belta-terpineol |
| LQ20 | 81722 | C10H18O | 154.25 | CC(C)(C1CCC(=C)CC1)O | delta-terpineol |
| LQ21 | 5317384 | C20H25NO3 | 327.4 | COC1CC23C(=CCN2CCCC4=CC(=C(C=C34)OC)OC)C=C1 | fortuneine |
| LQ22 | 5320290 | C16H14O6 | 302.28 | COC1=C(C(=C2C(=C1)OC3=C(C2=O)C=C(C=C3)O)OC)OC | onjixanthone i |
| LQ23 | 363707 | C8H16O3 | 160.21 | C1CC(CCC1O)(CCO)O | rengyol |
| LQ24 | 10725564 | C8H10O3 | 154.16 | C1COC2C1(C=CC(=O)C2)O | rengyolone |
| LQ25 | 14353410 | C8H14O3 | 158.19 | C1CC2(CCC1(O2)CCO)O | rengyoxide |
| LQ26 | 10487440 | C10H10O2 | 174.14 | C=CC[14C]1=[14CH][14C]2=[14C]([14CH]=[14CH]1)OCO2 | safrole |
| LQ27 | 8417 | C11H10O4 | 206.19 | COC1=C(C=C2C(=C1)C=CC(=O)O2)OC | scoparone |
| LQ28 | 10774324 | C10H14O4 | 198.22 | CC(=O)OC1CCC(=CC(=O)O)CC1 | suspenolicacid |
| LQ29 | 49867942 | C30H48O3 | 456.7 | CC1CC(C2CCC3(C(=CCC4C3(CCC5C4(CCC(C5(C)C)O)C)C)C2C1C)C)C(=O)O | ursolic acid |
| LQ30 | 442484 | C10H16 | 136.23 | CC(C)C1CCC(=C)C=C1 | ß-phellandrene |
| FF1 | 101866713 | C13H15NO5 | 265.2 | CCOC(=O)C1CCC(=O)N1CC(=O)C2=CC=CO2 | divaricataester,a |
| FF2 | 3083616 | C11H10O5 | 220.24 | COC1=C(C(=C2C(=C1)C=CC(=O)O2)O)OC | fraxidin |
| FF3 | 5385192 | C12H8O5 | 232.2 | COC1=C2C(=C(C3=C1OC=C3)O)C=CC(=O)O2 | 4-hydroxy-9-methoxyfuro[3,2-g]chromen-7-one |
| FF4 | 334704 | C14H14O4 | 246.28 | CC(C)(C1CC2=C(O1)C=C3C(=C2)C=CC(=O)O3)O | Marmesin |
| FF5 | 6184 | C6H12O | 100.18 | CCCCCC=O | hexanal |
| FF6 | 2758 | C10H18O | 154.28 | CC1(C2CCC(O1)(CC2)C)C | 1,8-cineole |
| FF7 | 442353 | C15H24 | 204.39 | CC1=CCC2(CC1)C(=C)CCCC2(C)C | beta-Chamigrene |
| FF8 | 7362 | C5H4O2 | 96.09 | C1=COC(=C1)C=O | Furol |
| FF9 | 11463 | C10H16 | 136.26 | CC1=CCC(=C(C)C)CC1 | Tereben |
| FF10 | 93081 | C15H24 | 204.39 | CC1CCC(C2C13C2C(=C)CC3)C(C)C | beta-Cubebene |
| FF11 | 11051711 | C10H16 | 136.26 | CC(C)C12CCC(=C)C1C2 | (1S,5S)-1-isopropyl-4-methylenebicyclo[3.1.0]hexane |
| FF12 | 439570 | C10H14O | 150.24 | CC1=CCC(CC1=O)C(=C)C | l-carvone |
| FF13 | 21883788 | C4H4O4 | 116.08 | [H+].[H+].C(=C/C(=O)[O-])\\C(=O)[O-] | FUM |
| FF14 | 10228 | C15H16O3 | 244.31 | CC(=CCC1=C(C=CC2=C1OC(=O)C=C2)OC)C | osthol |
| FF15 | 19602 | C9H14O | 138.23 | CCCCCC1=CC=CO1 | PENTYLFURAN |
| FF16 | 10812 | C10H14 | 134.24 | CC1=CC(=CC=C1)C(C)C | m-Cymol |
| FF17 | 5283349 | C10H16O | 152.26 | CCCCC/C=C/C=C/C=O | trans-2,4-decadienal |
| FF18 | 12444324 | C10H16 | 136.26 | CC1=CCC2(C1C2)C(C)C | (5S)-1-isopropyl-4-methylbicyclo[3.1.0]hex-3-ene |
| FF19 | 326 | C10H12O | 148.22 | CC(C)C1=CC=C(C=C1)C=O | cuminal |
| FF20 | 1252759 | C10H16O | 152.26 | CC1=CCC(C1(C)C)CC=O | 2-[(1R)-2,2,3-trimethyl-1-cyclopent-3-enyl]ethanal |
| FF21 | 88301 | C10H16O | 152.26 | CC1(C2CC=C(C1C2)CO)C | 19894-97-4 |
| FF22 | 12306047 | C15H24 | 204.39 | CC1=CC2C(CC1)C(=CCC2C(C)C)C | muurolene |
| FF23 | 92874 | C10H14O | 150.24 | CC1=CC(=O)C2CC1C2(C)C | l-Verbenone |
| FF24 | 1201529 | C10H14O | 150.24 | CC1(C2CC=C(C1C2)C=O)C | (1S,5R)-7,7-dimethyl-4-bicyclo[3.1.1]hept-3-enecarboxaldehyde |
| FF25 | 7410 | C8H8O | 120.16 | CC(=O)C1=CC=CC=C1 | Hypnon |
| FF26 | 68081 | C16H14O4 | 270.3 | CC(=CCOC1=C2C=CC(=O)OC2=CC3=C1C=CO3)C | isoimperatorin |
| FF27 | 11062489 | C10H16 | 136.26 | CC1(C2CCC1(C=C2)C)C | (1S,4R)-1,7,7-trimethylbicyclo[2.2.1]hept-2-ene |
| FF28 | 14529 | C10H14O | 150.24 | CC1=CC=C(C=C1)C(C)(C)O | p-Cymen-8-ol |
| FF29 | 8900 | C7H16 | 100.23 | CCCCCCC | Heptan |
| FF30 | 3083857 | C12H18O2 | 194.3 | CCCCC1C2CCCC=C2C(=O)O1 | 1(3H)-Isobenzofuranone, 3-butyl-3a,4,5,6-tetrahydro-, cis-(-)- |
| FF31 | 14257 | C11H24 | 156.35 | CCCCCCCCCCC | UND |
| FF32 | 931 | C10H8 | 128.18 | C1=CC=C2C=CC=CC2=C1 | naphthalene |
| FF33 | 636822 | C12H16O3 | 208.28 | C/C=C/C1=CC(=C(C=C1OC)OC)OC | Azaron |
| FF34 | 68079 | C13H10O5 | 246.23 | COC1=C2C=COC2=C(C3=C1C=CC(=O)O3)OC | isopimpinellin |
| FF35 | 21980959 | C2H4O2 | 60.06 | [H+].CC(=O)[O-] | acetic acid |
| FF36 | 5273569 | C10H8O5 | 208.18 | COC1=C(C(=C2C(=C1)C=CC(=O)O2)O)O | fraxetin |
| FF37 | 68082 | C12H8O4 | 216.2 | COC1=C2C=CC(=O)OC2=C3C=COC3=C1 | Isobergapten |
| FF38 | 8141 | C9H20 | 128.29 | CCCCCCCCC | nonane |
| FF39 | 6403 | C6H14 | 86.2 | CCC(C)(C)C | Neohexane |
| FF40 | 5318565 | C11H10O5 | 222.21 | COC1=C(C(=C2C(=C1)C=CC(=O)O2)OC)O | Phytodolor |
| FF41 | 10211 | C17H18O7 | 334.35 | CC(C)(C(COC1=C2C(=C(C3=C1OC(=O)C=C3)OC)C=CO2)O)O | Byakangelicin |
| FF42 | 22227 | C10H16O | 152.26 | CC1CCC(CC1=O)C(=C)C | d-Dihydrocarvone |
| FF43 | 8257 | C5H10O | 86.13 | CC(C)(C=C)O | 2-methylbut-3-en-2-ol |
| FF44 | 87436 | C6H9NS | 127.21 | C=CCCCN=C=S | 4-pentenyl isothiocyanate |
| FF45 | 40923 | C8H16O | 128.21 | CCCC(CCC=C)O | 7-octen-4-ol |
| FF46 | 5318539 | C15H22O | 218.33 | CC1=CC=C(C=C1)C2(CCC(C2(C)C)O)C | alpha-cuparenol |
| FF47 | 2355 | C12H8O4 | 216.19 | COC1=C2C=CC(=O)OC2=CC3=C1C=CO3 | bergapten |
| FF48 | 10286 | C9H6O2 | 146.14 | C1=CC=C2C(=C1)C(=O)C=CO2 | chromone |
| FF49 | 11084908 | C15H22 | 202.33 | CC1=CC=C(C=C1)C2(CCCC2(C)C)C | cuparene |
| FF50 | 5317956 | C22H28O5 | 372.5 | CC1C(C2C(=O)C1(C=C(C2(C)O)OC)CC=C)C3=CC(=C(C=C3)OC)OC | hancinol |
| FF51 | 12398 | C17H36 | 240.5 | CCCCCCCCCCCCCCCCC | heptadecane |
| FF52 | 53628050 | C6H10O | 98.14 | [CH2-]CCCCC#[O+] | hexanal |
| FF53 | 53627517 | C6H12O | 100.16 | [CH2+]CCCCC[O-] | hexanol |
| FF54 | 442977 | C27H43NO3 | 429.6 | CC1CCC2C(C3CCC4C(C3CN2C1)CC5C4CC(=O)C6C5(CCC(C6)O)C)(C)O | imperialine |
| FF55 | 356 | C8H18 or CH3-(CH2)6-CH3 | 114.23 | CCCCCCCC | n-octane |
| FF56 | 177751 | C14H12O4 | 244.24 | CC1(CC(=O)C2=C(O1)C=C3C(=C2)C=CC(=O)O3)C | naphthalene |
| FF57 | 10965344 | C10H8O4 | 192.17 | C1CC(=O)C2=C(C=CC(=C2C1=O)O)O | naphthazarin |
| FF58 | 8063 | C5H10O or CH3(CH2)3CHO | 86.13 | CCCCC=O | pentanal |
| FF59 | 98608 | C17H16O5 | 300.3 | CC(=CCOC1=C2C(=C(C3=C1OC(=O)C=C3)OC)C=CO2)C | phellopterin |
| FF60 | 6054 | C8H10O or C6H5CH2CH2OH | 122.16 | C1=CC=C(C=C1)CCO | phenethyl alcohol |
| FF61 | 6199 | C11H6O3 | 186.16 | C1=CC(=O)OC2=CC3=C(C=CO3)C=C21 | psoralen |
| FF62 | 14260 | C14H28 or CH3(CH2)11CH=CH2 | 196.37 | CCCCCCCCCCCCC=C | tetradecane |
| FF63 | 10104370 | C15H24 | 204.35 | CC1=CCC(CC1)C(=C)CCC=C(C)C | ß-bisabolene |
| HX1 | 12304985 | C15H26O | 222.41 | CC1CCC2C1C3C(C3(C)C)CCC2(C)O | 49070_FLUKA |
| HX2 | 5317844 | C15H24 | 204.39 | CC1CCC(CC2=C1CCC2C)C(=C)C | alpha-Guaiene |
| HX3 | 6949 | C15H24 | 204.39 | CC1CCC(=C(C)C)CC2=C1CCC2C | guaiene |
| HX4 | 6584 | C3H6O2 | 74.09 | CC(=O)OC | Tereton |
| HX5 | 10446 | C20H38 | 278.58 | CC(C)CCCC(C)CCCC(C)CCCC(=C)C=C | neophytadiene |
| HX6 | 6428995 | C9H8O | 132.17 | C1=CC=C(C=C1)/C=C\\C=O | cis-Cinnamaldehyde |
| HX7 | 16212927 | C15H24 | 204.39 | CC(C)C1CCC2(C3C1C4C2(C4C3)C)C | ()-Cyclosativene |
| HX8 | 101731 | C15H24 | 204.39 | CC1CCC2=C1CC3CCC2(C3(C)C)C | ß-patchoulene |
| HX9 | 5281168 | C6H10O | 98.16 | CCC/C=C/C=O | Hexenal |
| HX10 | 12306055 | C15H24 | 204.39 | CC1=CC2C(CCC(=C2CC1)C)C(C)C | d-cadinol |
| HX11 | 18554 | C6H8O | 96.14 | CCC1=CC=CO1 | ETHYL FURAN |
| HX12 | 11401461 | C15H24 | 204.39 | CC1=CCC2(CCCC(C23C1C3)(C)C)C | widdrene |
| HX13 | 995 | C14H10 | 178.24 | C1=CC=C2C(=C1)C=CC3=CC=CC=C32 | PEY |
| HX14 | 165536 | C15H22O | 218.37 | CC1CCCC2=CC(=O)C3C(C12C)C3(C)C | Aristolone |
| HX15 | 11251 | C5H10O | 86.15 | CC(C)C(=O)C | MIPK |
| HX16 | 7013 | C16H13N | 219.3 | C1=CC=C(C=C1)NC2=CC=CC3=CC=CC=C32 | PANA |
| HX17 | 521710 | C15H24 | 204.39 | CC1CCC23C1CC(C2(C)C)CC=C3C | a-patchoulene |
| HX18 | 13143 | C5H8O | 84.13 | CC(=C)C(=O)C | Methyl butenone |
| HX19 | 519743 | C15H24 | 190.36 | CC1CCC2(C(=C)C3CCC2(C1C3)C)C | seychellene |
| HX20 | 3362 | C9H10O4 | 182.19 | CCC(=O)C1=C(C=C(C=C1O)O)O | Labroda |
| HX21 | 6428535 | C15H24 | 204.39 | CC1=CC2C(CC1)C(=CCCC2(C)C)C | 53111-25-4 |
| HX22 | 54695756 | C12H16O4 | 224.28 | CC1=CC(=C(C(=O)O1)C(=O)CCC(C)C)O | DHELWANGIN |
| HX23 | 543966 | C13H24 | 180.37 | CC1(CCCC1C2CCCCC2)C | 2,2-Dimethylcyclopentylcyclohexane |
| HX24 | 8766 | C12H10S | 186.29 | C1=CC=C(C=C1)SC2=CC=CC=C2 | WLN: RSR |
| HX25 | 90971 | C15H22O | 218.37 | CC1(CC(=O)C=C2C13CCC(C3)C2(C)C)C | 1,2,3,4,5,6-hexahydro-1,1,5,5-tetramethyl-7H-2,4a-methanonaphthalen-7-one |
| HX26 | 13072631 | C8H18O2 | 146.26 | CC(C)C(C(C(C)C)O)O | (3S,4R)-2,5-dimethylhexane-3,4-diol |
| HX27 | 101596917 | C15H24 | 204.39 | CC1=C2C3C(C3(C)C)CCC2(CCC1)C | ß-maaliene |
| HX28 | 5281781 | C17H14O6 | 314.31 | COC1=CC=C(C=C1)C2=COC3=C(C2=O)C(=C(C(=C3)O)OC)O | irisolidone |
| HX29 | 565709 | C15H24 | 204.39 | CC1CCC(CC2=C(CCC12)C)C(=C)C | Aciphyllene |
| HX30 | 12314136 | C14H10O | 293.34 | C1C2=CC=CC=C2C3=CC=CC=C3C1=O | phenanthrone |
| HX31 | 240 | C7H6O | 106.13 | C1=CC=C(C=C1)C=O | WLN: VHR |
| HX32 | 5319618 | C21H28O5 | 360.4 | CC1CCC2(C(C1=C)CC(=O)C3=C2C(=C(C(=C3O)C(C)(C)O)O)OC)C | 1-methylene-2,4a-dimethyl-6,8-dihydroxy-5-methoxy-7-(1,1-dimethylhydroxymethyl)-1,2,3,4,9,10,10a-heptahydro-9-phenanthrone |
| HX33 | 7163260 | C30H48O4 | 472.7 | CC1(CCC2(CCC3(C(=CCC4C3(CCC5C4(CC(C(C5(C)C)O)O)C)C)C2C1)C)C(=O)O)C | 2a,3ß-dihydroxyolean-12-en-28-oicacid |
| HX34 | 5319620 | C16H9NO4 | 279.25 | C1OC2=C(O1)C3=C4C=C(C=CC4=CC5=C3C(=C2)C(=O)N5)O | 3,4-methylenedioxy-10-hydroxy aristololactam |
| HX35 | 246728 | C8H16O | 128.21 | CCCCCC(=O)CC | 3-octanone |
| HX36 | 97102 | C20H19NO2S2 | 369.5 | CC1=CC=C(C=C1)S(=O)(=O)N=S(CC2=CC=CC=C2)C3=CC=CC=C3 | S-Benzyl-S-phenyl-N-tosylsulfimine |
| HX37 | 5317859 | C15H24 | 204.35 | CC1CCC2C(C2(C)C)C3=C(CCC13)C | alpha-guriunene |
| HX38 | 11252044 | C8H8O2 | 137.15 | COC1=CC=C(C=C1)C=O | anisaldehyde |
| HX39 | 14896 | C10H16 | 136.23 | CC1(C2CCC(=C)C1C2)C | beta-pinene |
| HX40 | 6432176 | C15H24 | 204.35 | CC1CCC=C2C1(C3C(C3(C)C)CC2)C | calarene |
| HX41 | 14350 | C15H24O | 220.35 | CC1(CC2C1CCC3(C(O3)CCC2=C)C)C | caryophyllene epoxid |
| HX42 | 6429077 | C15H22 | 202.33 | CC1CCC(C2=C1C=CC(=C2)C)C(C)C | cis-calamenene |
| HX43 | 7058172 | C4H9N3O2 | 131.13 | C[N+](=C(N)N)CC(=O)[O-] | creatine |
| HX44 | 10248 | C12H16O3 | 208.25 | COC1=CC(=CC(=C1OC)OC)CC=C | elemicin |
| HX45 | 101341 | C30H52O | 428.7 | CC1C(CCC2C1(CCC3C2(CCC4(C3(CCC5(C4CC(CC5)(C)C)C)C)C)C)C)O | epifriedelanol |
| HX46 | 5317394 | C32H54O2 | 470.8 | CC1C(CCC2C1(CCC3C2(CCC4(C3(CCC5(C4CC(CC5)(C)C)C)C)C)C)C)OC(=O)C | epifriedelanol acetate |
| HX47 | 244297 | C30H50O | 426.7 | CC1C(=O)CCC2C1(CCC3C2(CCC4(C3(CCC5(C4CC(CC5)(C)C)C)C)C)C)C | friedelan-3-one |
| HX48 | 24893863 | C20H33ClO | 324.9 | CC1(CCCC2(C1CCC34C2CCC(C3)C(C4)(CCl)O)C)C | fritillaziebinol |
| HX49 | 5463721 | C15H24O | 220.35 | CC1=CCC(C=CCC2(C(O2)CC1)C)(C)C | humulene epoxide i |
| HX50 | 6325415 | C20H26N2O2 | 326.4 | CCC1C2CC3C4C5(CC(C2C5O)N3C1O)C6=CC=CC=C6N4C | isoajmaline |
| HX51 | 520190 | C20H34O | 290.5 | CC1=CCCC(C=CC(CCC(=CCC1)C)C(C)C)(C)O | isocembrol |
| HX52 | 8815 | C10H12O | 148.2 | COC1=CC=C(C=C1)CC=C | methylchavicol |
| HX53 | 49867939 | C30H48O3 | 456.7 | CC1(CC2C(CCC3(C2=CCC4C3(CCC5C4(CCC(C5(C)C)O)C)C)C)C(C1)C(=O)O)C | oleanolic acid |
| HX54 | 7463 | C10H14 or CH3C6H4CH(CH3)2 | 134.22 | CC1=CC=C(C=C1)C(C)C | p-cymene |
| HX55 | 641294 | C10H10O2 | 162.18 | COC1=CC=C(C=C1)C=CC=O | p-methoxycinnamaldehyde |
| HX56 | 5285554 | C10H9O3- | 177.18 | COC1=CC=C(C=C1)C=CC(=O)[O-] | p-methoxycinnamic acid |
| HX57 | 101277 | C19H12O6 | 336.3 | COC1=CC2=C(C=C1C3=CC4=C(C=C5C(=C4)C=CO5)OC3=O)OCO2 | pachyrhizin |
| HX58 | 10955174 | C15H26O | 222.37 | CC1CCC2(C(C3CCC2(C1C3)C)(C)C)O | patchoulicalcohol |
| HX59 | 5318042 | C6H12O | 100.16 | CCCC=CCO | trans-2-hexenol |
| HX60 | 18502770 | C14H22 | 190.32 | CC1=CCC(=C)C2CC(C2CC1)(C)C | ß-caryophyllene |
| HX61 | 6918391 | C15H24 | 204.35 | CC(=C)C1CCC(C(C1)C(=C)C)(C)C=C | ß-elemene |
| HX62 | 5281167 | C6H12O | 100.16 | CCC=CCCO | ß-hexenol |
| HX63 | 12313023 | C15H24 | 204.35 | CC1=CC2C(CC1)C(=C)CCC2C(C)C | cadinene |
| HX64 | 11320910 | C6H12O | 100.16 | CCCC/C=C/O | hexenol |
| HX65 | 14829106 | C32H50O3 | 482.7 | CC(=O)OC1CCC2(C(C1(C)C)CCC3(C2CC=C4C3(CCC5(C4CC(CC5)(C)C)C=O)C)C)C | O-Acetyloleanolic aldehyde |
| HX66 | 643139 | C6H10O | 98.14 | CC/C=C/CC=O | 3-Hexenal |
| HX67 | 26634 | C13H18N2O | 218.29 | CC(C(=O)NC1CCC2=CC=CC=C2C1)N | 2-Amino-N-(1,2,3,4-tetrahydronaphthalen-2-yl)propanamide |
| HX68 | 101607926 | C15H24 | 204.35 | CC1=CCC2C3C1C2(CCC3C(C)C)C | (1S,2S,6S,7R,8R)-1,3-Dimethyl-8-propan-2-yltricyclo[4.4.0.02,7]dec-3-ene |
| HX69 | 8658 | C8H8O2 | 136.15 | COC1=CC=CC=C1C=O | 2-Methoxybenzaldehyde |
| HX70 | 6654 | C10H16 | 136.23 | CC1=CCC2CC1C2(C)C | alpha-Pinene |
| YCH1 | 8468 | C8H8O4 | 168.15 | COC1=C(C=CC(=C1)C(=O)O)O | vanillic acid |
| YCH2 | 159055 | C10H16O | 152.23 | CC1(C2CCC1(C(=O)C2)C)C | CAM |
| YCH3 | 7461 | C10H16 | 136.23 | CC1=CCC(=CC1)C(C)C | Moslene |
| YCH4 | 2214 | C9H10O3 | 166.17 | CC(=O)C1=CC(=C(C=C1)O)OC | Apocynin |
| YCH5 | 5281331 | C29H48O | 412.7 | CCC(C=CC(C)C1CCC2C1(CCC3C2=CCC4C3(CCC(C4)O)C)C)C(C)C | Spinasterol |
| YCH6 | 2724161 | C10H18O | 154.25 | CC1=CCC(CC1)(C(C)C)O | ()-Terpinen-4-ol |
| YCH7 | 1183 | C8H8O3 | 152.15 | COC1=C(C=CC(=C1)C=O)O | vanillin |
| YCH8 | 237332 | C6H6O3 | 126.11 | C1=C(OC(=C1)C=O)CO | HMF |
| YCH9 | 15304 | C16H14 | 206.28 | CC1=CC2=C(C=C1)C=CC3=C2C=C(C=C3)C | 3,6-Dimethylphenanthrene |
| YCH10 | 717531 | C11H12O4 | 208.21 | COC1=C(C=C(C=C1)/C=C/C(=O)O)OC | Dimethylcaffeic acid |
| YCH11 | 3080632 | C29H50O | 414.7 | CCC(CCC(C)C1CCC2C1(CCC3C2=CCC4C3(CCC(C4)O)C)C)C(C)C | stigmast-7-enol |
| YCH12 | 11223011 | C20H22O8 | 390.4 | COC1=CC(=CC(=C1O)C2=C(C(=CC(=C2)CCC(=O)O)OC)O)CCC(=O)O | 3-[3-[5-(2-carboxyethyl)-2-hydroxy-3-methoxyphenyl]-4-hydroxy-5-methoxyphenyl]propanoic acid |
| YCH13 | 11244683 | C18H17N3O4 | 339.3 | CC(=O)C1=C2C(=CC(=N1)C(=O)NCCC(=O)OC)C3=CC=CC=C3N2 | dichotomide i |
| YCH14 | 11242284 | C14H12N2O3 | 256.26 | CC(C1=C2C(=CC(=N1)C(=O)O)C3=CC=CC=C3N2)O | dichotomine a |
| YCH15 | 155331 | C22H31NO5 | 389.5 | CCC1C2C3CCC(N3CCCC2OC14C=C(C(=O)O4)C)C5CC(C(=O)O5)C | stemonine |
| YCH16 | 5281703 | C16H12O5 | 284.26 | COC1=C(C=C(C2=C1OC(=CC2=O)C3=CC=CC=C3)O)O | wogonin |
| BZ1 | 11379068 | C15H26O | 222.37 | CC1CCC=C(C12CCC(C2)C(C)(C)O)C | hinesol |
| BZ2 | 160782 | C12H11NO3 | 217.22 | CCOC(=O)C1=CC(=O)NC2=CC=CC=C21 | 4-ethoxycarbonyl-2-quinolone |
| BZ3 | 5317270 | C15H26O | 222.37 | CC12CCCC(=C)C1CC(CC2)C(C)(C)O | beta-eudesmol |
| BZ4 | 5321047 | C13H10O | 182.22 | CC=CC#CC#CC=CC1=CC=CO1 | atractylodin |
| BZ5 | 3080635 | C15H20O | 216.32 | CC1=COC2=C1CC3C(=C)CCCC3(C2)C | atractylone |
| BZ6 | 10813930 | C30H38O4 | 462.62 | CC1=C2CC3C(=C)CCCC3(CC2(OC1=O)C45CC6(CCCC(=C)C6CC4=C(C(=O)O5)C)C)C | Biatractylolide |
| BZ7 | 442360 | C15H22 | 202.37 | CC1=CC=C(C=C1)C(C)CCC=C(C)C | α-curcumene( R-) |
| BZ8 | 9215 | C13H9N | 179.23 | C1=CC=C2C(=C1)C=C3C=CC=CC3=N2 | Akridin |
| BZ9 | 11311230 | C15H20O3 | 248.32 | CC1=C2CC3C(=C)CCCC3(CC2(OC1=O)O)C | atractylenolide iii |
| BZ10 | 92221 | C10H16 | 136.26 | CC1(C2CCC(C2)C1=C)C | D-Camphene |
| BZ11 | 11368212 | C15H18O2 | 230.3 | CC1=C2CC3C(=C)CCCC3(C=C2OC1=O)C | atractylenolide |
| BZ12 | 440917 | C10H16 | 136.26 | CC1=CCC(CC1)C(=C)C | Hemo-sol |
| BZ13 | 442393 | C15H24 | 204.39 | CC(=C)C1CCC2(CCCC(=C)C2C1)C | beta-Selinene |
| BZ14 | 443166 | C10H18O | 154.28 | CC1CCC(CC1O)C(=C)C | (1S,2R,4R)-Neoiso-dihydrocarveol |
| BZ15 | 1493692 | C15H24 | 204.39 | CC1=CCC2C3C1C2(CCCC3(C)C)C | α-Longipinene |
| BZ16 | 5281515 | C15H24 | 204.39 | CC1=CCCC(=C)C2CC(C2CC1)(C)C | beta-caryophyllene |
| BZ17 | 5283650 | C29H50O | 414.79 | CCC(C=CC(C)C1CCC2C1(CCC3C2CCC4C3(CCC(C4)O)C)C)C(C)C | stigmast-22E-en-3beta-ol |
| BZ18 | 5318102 | C15H24 | 204.39 | CC1=CCC(C=CCC(=C)CCC1)(C)C | β-humulene |
| BZ19 | 5318734 | C15H26O | 222.37 | CC(=C1CCC2(CCCC(C2C1)(C)O)C)C | juniper camphor |
| BZ20 | 6321405 | C10H18O | 154.28 | CC1(C2CCC1(C(C2)O)C)C | (+/-)-Isoborneol |
| BZ21 | 6971047 | C5H9NO2 | 115.15 | C1CC([NH2+]C1)C(=O)[O-] | Prolinum |
| BZ22 | 7015153 | C10H12O | 148.22 | CC(=C)C(C1=CC=CC=C1)O | (1R)-2-methyl-1-phenylprop-2-en-1-ol |
| BZ23 | 9601230 | C15H20O | 216.35 | CC1=CCC2=C(CC(=CCC1)C)OC=C2C | (5E,9Z)-3,6,10-trimethyl-4,7,8,11-tetrahydrocyclodeca[b]furan |
| BZ24 | 11106487 | C15H24 | 204.39 | CC(CCC=C(C)C)C1CCC(=C)C=C1 | (3S)-3-[(1R)-1,5-dimethylhex-4-enyl]-6-methylenecyclohexene |
| BZ25 | 12309452 | C15H24 | 204.39 | CC(=C1CCC(C(C1)C(=C)C)(C)C=C)C | γ-elemene |
| BZ26 | 14448070 | C15H20O2 | 232.35 | CC1=C2CC3C(=C)CCCC3(CC2OC1=O)C | atractylenolideII |
| BZ27 | 15976101 | C30H52O | 428.82 | CCCC(CCC(C)C1CCC2C1(CCC3C2CC=C4C3(CCC(C4)O)C)C)C(C)C | (3S,8S,9S,10R,13R,14S,17R)-10,13-dimethyl-17-[(2R,5S)-5-propan-2-yloctan-2-yl]-2,3,4,7,8,9,11,12,14,15,16,17-dodecahydro-1H-cyclopenta[a]phenanthren-3-ol |
| BZ28 | 44584667 | C15H24 | 204.39 | CC1CCC2C1C3C(C3(C)C)CCC2=C | alloaromadedrene |
| BZ29 | 5281520 | C15H24 | 204.35 | CC1=CCC(C=CCC(=CCC1)C)(C)C | α-humulene |
| BZ30 | 92138 | C15H26O | 222.41 | CC(=C)C1CC(CCC1(C)C=C)C(C)(C)O | 2-[(1R,3S,4S)-3-isopropenyl-4-methyl-4-vinylcyclohexyl]propan-2-ol |
| GC1 | 5316801 | C25H26O5 | 406.5 | CC(=CCC1=C(C=CC(=C1O)C(=O)C=CC2=CC(=C3C(=C2)C=CC(O3)(C)C)O)O)C | (e)-1-[2,4-dihydroxy-3-(3-methyl-2-butenyl)phenyl]-3-(2,2-dimethyl-8-hydroxy-2h-benzo-pyran-6-yl)-2-propen-1-one |
| GC2 | 124702 | C6H13NO4 | 163.17 | C(C1C(C(C(N1)CO)O)O)O | 2,5-dihydroxymethyl-3,4-dihydroxypyrrolidine |
| GC3 | 5319801 | C15H10O5 | 270.24 | CC1=C(C=C2C(=C1O)C(=O)C3=C(C2=O)C=C(C=C3)O)O | 2-methyl-1,3,6-trihydroxyanthraquinone |
| GC4 | 54691413 | C10H9O4- | 193.18 | COC1=C(C=CC(=C1)C=CC(=O)O)[O-] | ferulic acid |
| GC5 | 5317483 | C21H20O7 | 384.4 | CC(=CCC1=C(C=C2C(=C1O)C(=O)C(=C(O2)C3=CC(=C(C=C3)O)OC)O)O)C | gancaonin p-3'-methylether |
| GC6 | 10800959 | C15H26O3 | 254.36 | CC1C(CC2(C1(C3CC(CC3(C2)O)(C)C)C)O)O | gloeosteretriol |
| GC7 | 5311038 | C34H50O7 | 570.8 | CC1(C2CCC3(C(C2(CCC1OC(=O)CCC(=O)O)C)C(=O)C=C4C3(CCC5(C4CC(CC5)(C)C(=O)O)C)C)C)C | glycyrrhetinicacid |
| GC8 | 10479222 | C30H48O3 | 456.7 | CC1(C2CCC3(C(C2(CCC1O)C)C(=O)C=C4C3(CCC5(C4CC(CC5)(C)CO)C)C)C)C | glycyrrhetol |
| GC9 | 11791469 | C15H18O3 | 246.3 | CC1CC2C(CC(=O)C3=COC(=C23)C1=O)C(C)C | gmelofuran |
| GC10 | 148619 | C16H14O5 | 286.2 | CC(=C)C(COC1=C2C(=CC3=C1OC=C3)C=CC(=O)O2)O | isogosferol |
| GC11 | 12311086 | C21H23NO2 | 321.4 | C1CC(NC(C1)CC(=O)C2=CC=CC=C2)CC(=O)C3=CC=CC=C3 | isolobelanine |
| GC12 | 5319678 | C10H12O5 | 212.2 | COC1=C(C(=C(C=C1)C(=O)OC)O)OC | methyl 2-hydroxy-3,4-dimethoxy benzoate |
| GC13 | 5319677 | C31H50O4 | 486.7 | CC12CCC(CC1C3=CCC4C5(CCC(C(C5CCC4(C3(CC2)C)C)(C)CO)O)C)(C)C(=O)OC | methyl-24-hydroxy-11-deoxoglycyrrhetate |
| GC14 | 5319681 | C31H48O5 | 500.7 | CC12CCC(CC1C3=CC(=O)C4C5(CCC(C(C5CCC4(C3(CC2)C)C)(C)CO)O)C)(C)C(=O)OC | methyl-24-hydroxyglycyrrhetate |
| GC15 | 102135 | C31H48O4 | 484.7 | CC1(C2CCC3(C(C2(CCC1O)C)C(=O)C=C4C3(CCC5(C4CC(CC5)(C)C(=O)OC)C)C)C)C | methylglycyrrhetate |
| GC16 | 10446822 | C17H19NO3 | 288.36 | CN1CCC23C=CC(=O)CC2OC4=C(C=CC(=C34)C1)OC | narwedine |
| GC17 | 6553885 | C10H18O | 154.25 | CC1CCC(C(C1)O)C(=C)C | neoisopulegol |
| GC18 | 442429 | C10H18O2 | 170.25 | CC1CCC2C1C(OCC2C)O | neomatatabiol |
| GC19 | 54710960 | C11H11O5- | 223.2 | COC1=CC(=CC(=C1[O-])OC)C=CC(=O)O | sinapic acid |
| GC20 | 15818599 | C25H26O6 | 422.5 | CC(=CCC1=C(C(=CC(=C1)C2=COC3=C(C2=O)C(=CC(=C3CC=C(C)C)O)O)O)O)C | glyurallin a |
| GC21 | 354368 | C17H14O3 | 266.31 | CC1=C(C(=O)C2=C(O1)C=C(C=C2)OC)C3=CC=CC=C3 | 7-methoxy-2-methylisoflavone |
| GC22 | 72 | C7H6O4 | 154.13 | C1=CC(=C(C=C1C(=O)O)O)O | protocatechuic acid |
| GC23 | 443162 | C10H18O | 154.28 | CC1=CCC(CC1)C(C)(C)O | (L)-alpha-Terpineol |
| GC24 | 69867 | C9H7NO2 | 161.17 | C1=CC=C2C(=C1)C(=CN2)C(=O)O | ICO |
| GC25 | 6544 | C9H14O | 138.23 | CC1=CC(=O)CC(C1)(C)C | Izoforon |
| GC26 | 185667 | C10H13N | 147.24 | CC1=C2CCCCC2=NC=C1 | 5,6,7,8-tetrahydro-4-methylquinoline |
| GC27 | 16211586 | C10H16 | 136.26 | CC1CC2C(C2(C)C)C=C1 | 21987_FLUKA |
| GC28 | 10494 | C30H48O3 | 456.78 | CC1(CCC2(CCC3(C(=CCC4C3(CCC5C4(CCC(C5(C)C)O)C)C)C2C1)C)C(=O)O)C | oleanolic acid |
| GC29 | 5320118 | C20H18O7 | 370.38 | CC(=CCC1=C(C=CC(=C1O)O)C2=C(C(=O)C3=CC(=C(C=C3O2)O)O)O)C | Neouralenol |
| GC30 | 131751571 | C29H42O5 | 484.7 | CC1(C2CCC3(C(C2(CCC1O)C)C(=O)C=C4C3(CC5C6(C4CC(C(C6)O5)(C)C(=O)O)C)C)C)C | liquoric acid |
| GC31 | 12678291 | C10H14O | 150.24 | CC1(C2C1C=C(CC2)C=O)C | 2-Caren-10-al |
| GC32 | 5280448 | C16H12O5 | 284.28 | COC1=C(C=C(C=C1)C2=COC3=C(C2=O)C=CC(=C3)O)O | Calycosin |
| GC33 | 5281704 | C17H14O5 | 298.31 | COC1=CC=C(C=C1)C2=COC3=CC(=C(C=C3C2=O)OC)O | Castanin |
| GC34 | 66841 | C10H16 | 136.26 | CC(C)C1=CCC(=C)CC1 | beta-Terpinene |
| GC35 | 637563 | C10H12O | 148.22 | CC=CC1=CC=C(C=C1)OC | anethole |
| GC36 | 131753069 | C26H28O6 | 436.54 | CC(=CCC1=C(C=CC(=C1O)C2=COC3=CC(=C(C(=C3C2=O)O)CC=C(C)C)OC)O)C | kanzonol K |
| GC37 | 192490 | C21H20O7 | 384.41 | CC(=CCC1=CC(=C(C=C1C2=C(C(=O)C3=C(O2)C=CC(=C3O)O)OC)O)O)C | uralene |
| GC38 | 5281619 | C20H18O5 | 338.38 | CC(=CCC1=C2C(=C(C=C1O)O)C(=O)C(=C(O2)C3=CC=CC=C3)O)C | Glepidotin A |
| GC39 | 8698 | C11H14O2 | 178.25 | CCCCOC(=O)C1=CC=CC=C1 | Butyl benzoate |
| GC40 | 480777 | C21H22O5 | 354.43 | CC(=CCC1=C(C=C2C(=C1OC)C=C(O2)C3=C(C=C(C=C3)O)O)OC)C | Gancaonin I |
| GC41 | 8433 | C15H12O2 | 224.27 | C1=CC=C(C=C1)C(=O)CC(=O)C2=CC=CC=C2 | Karenzu DK2 |
| GC42 | 10090416 | C21H20O6 | 368.41 | CC(C)(C=C)C1=C2C(=C(C=C1O)OC)C=C(C(=O)O2)C3=C(C=C(C=C3)O)O | 3-(2,4-dihydroxyphenyl)-8-(1,1-dimethylprop-2-enyl)-7-hydroxy-5-methoxy-coumarin |
| GC43 | 12305517 | C30H48O3 | 456.78 | CC1(C2CCC3(C(C2(CCC1O)C)CC=C4C3(CCC5(C4CC(CC5)(C)C(=O)O)C)C)C)C | 11-deoxyglycyrrhetic acid |
| GC44 | 11349817 | C25H26O4 | 390.51 | CC(=CCC1=CC2=C(C=C1O)OC(=CC2=O)C3=CC(=C(C=C3)O)CC=C(C)C)C | 7-hydroxy-2-[4-hydroxy-3-(3-methylbut-2-enyl)phenyl]-6-(3-methylbut-2-enyl)chromone |
| GC45 | 112111 | C28H42O4 | 470.7 | CC1(C2CCC3(C(C2(CCC1O)C)C(=O)C=C4C3(CCC5(C4CC(CC5)(C)C(=O)O)C)C)C)C | liguiritic acid |
| GC46 | 480787 | C22H22O6 | 382.44 | CC(=CCC1=C(C=C2C(=C1OC)C=C(C(=O)O2)C3=C(C=C(C=C3)O)O)OC)C | glycyrin |
| GC47 | 14604077 | C20H18O6 | 354.38 | CC(=CCC1=C2C(=C(C=C1O)O)C(=O)C(=CO2)C3=CC(=C(C=C3)O)O)C | 3-(3,4-dihydroxyphenyl)-5,7-dihydroxy-8-(3-methylbut-2-enyl)chromone |
| GC48 | 14604080 | C21H20O6 | 368.41 | CC(=CCC1=C(C2=C(C=C1O)OC=C(C2=O)C3=C(C=C(C=C3)OC)O)O)C | 5,7-dihydroxy-3-(2-hydroxy-4-methoxy-phenyl)-6-(3-methylbut-2-enyl)chromone |
| GC49 | 14604081 | C20H18O6 | 354.38 | CC(=CCC1=C(C2=C(C=C1O)OC(=CC2=O)C3=CC(=C(C=C3)O)O)O)C | 2-(3,4-dihydroxyphenyl)-5,7-dihydroxy-6-(3-methylbut-2-enyl)chromone |
| GC50 | 5481966 | C20H18O7 | 370.38 | CC(=CCC1=C(C2=C(C=C1O)OC(=C(C2=O)O)C3=CC(=C(C=C3)O)O)O)C | Gancaonin P |
| GC51 | 10361658 | C21H20O6 | 368.41 | CC(=CCC1=C(C(=C(C=C1O)O)C2=COC3=C(C2=O)C=CC(=C3)O)OC)C | 3-[4,6-dihydroxy-2-methoxy-3-(3-methylbut-2-enyl)phenyl]-7-hydroxy-chromone |
| GC52 | 503731 | C20H20O5 | 340.4 | CC(=CCC1=C(C=C2C(=C1OC)C=C(O2)C3=C(C=C(C=C3)O)O)O)C | licocoumarone |
| GC53 | 195396 | C31H44O6 | 512.75 | CC1(C2CCC3(C(C2(CCC1O)C)C(=O)C=C4C3(CCC5(C4CC6(CC5OC6=O)C)C)C(=O)OC)C)C | 3,22-Dihydroxy-11-oxo-delta(12)-oleanene-27-alpha-methoxycarbonyl-29-oic acid |
| GC54 | 11558452 | C16H10O6 | 298.26 | COC1=CC2=C(C=C1)C3=C(O2)C4=C(C=C(C=C4OC3=O)O)O | 1,3-dihydroxy-9-methoxy-6-benzofurano[3,2-c]chromenone |
| GC55 | 11602329 | C17H12O7 | 328.29 | COC1=C(C=C2C(=C1)C3=C(O2)C4=C(C=C(C=C4OC3=O)O)O)OC | 1,3-dihydroxy-8,9-dimethoxy-6-benzofurano[3,2-c]chromenone |
| GC56 | 5317300 | C20H18O5 | 338.38 | CC(=CCC1=C(C=CC(=C1O)C2=COC3=C(C2=O)C=CC(=C3)O)O)C | Eurycarpin A |
| GC57 | 15559941 | C30H44O4 | 468.74 | CC1(C2CCC3(C(C2(CCC1O)C)C(=O)C=C4C3(CCC5(C46CC(CC5)(C(=O)O6)C)C)C)C)C | isoglabrolide |
| GC58 | 3764 | C16H12O4 | 268.28 | COC1=CC2=C(C=C1)C(=O)C(=CO2)C3=CC=C(C=C3)O | HMO |
| GC59 | 19814101 | C30H46O5 | 486.76 | CC12CCC(CC1C3=CC(=O)C4C5(CCC(C(C5CCC4(C3(CC2)C)C)(C)CO)O)C)(C)C(=O)O | 24-Hydroxyglycyrrhetic acid |
| GC60 | 10331849 | C15H12O3 | 240.27 | C1=CC=C(C=C1)C=CC(=O)C2=C(C=C(C=C2)O)O | (Z)-1-(2,4-dihydroxyphenyl)-3-phenylprop-2-en-1-one |
| GC61 | 6478421 | C16H14O6 | 302.28 | COC1=C(C=CC(=C1O)O)C=CC(=O)C2=CC(=C(C=C2)O)O | 3,4,3',4'-Tetrahydroxy-2-methoxychalcone |
| GC62 | 5280460 | C10H8O4 | 192.18 | COC1=C(C=C2C(=C1)C=CC(=O)O2)O | scopoletin |
| GC63 | 222284 | C29H50O | 414.7 | CCC(CCC(C)C1CCC2C1(CCC3C2CC=C4C3(CCC(C4)O)C)C)C(C)C | β-sitosterol |
| GC64 | 136419 | C17H14O4 | 282.31 | COC1=CC=C(C=C1)C2=COC3=C(C2=O)C=CC(=C3)OC | Daidzein dimethyl ether |
| GC65 | 25015742 | C16H12O6 | 300.28 | COC1=CC(=CC2=C1C=C(C(=O)O2)C3=C(C=C(C=C3)O)O)O | 7,2',4'-trihydroxy－5-methoxy-3－arylcoumarin |
| GC66 | 268208 | C18H14O4 | 294.32 | CC1=C(C(=O)C2=C(O1)C=C(C=C2)OC(=O)C)C3=CC=CC=C3 | 7-Acetoxy-2-methylisoflavone |
| GC67 | 5380976 | C16H12O3 | 252.28 | CC1=C(C(=O)C2=C(O1)C=C(C=C2)O)C3=CC=CC=C3 | 7-hydroxy-2-methyl-3-phenyl-chromone |
| GC68 | 5481949 | C25H24O6 | 420.49 | CC(=CCC1=C(C2=C(C=C1O)OC=C(C2=O)C3=CC4=C(C(=C3)O)OC(C=C4)(C)C)O)C | Gancaonin H |
| GC69 | 5317478 | C21H20O5 | 352.41 | CC(=CCC1=C(C=C2C(=C1O)C(=O)C(=CO2)C3=CC=C(C=C3)OC)O)C | gancaonin A |
| GC70 | 15516846 | C25H24O4 | 388.49 | CC(=CCC1=CC2=C(C=C1O)OC(=CC2=O)C3=CC4=C(C=C3)OC(C=C4)(C)C)C | Kanzonol E |
| GC71 | 13965473 | C17H14O6 | 314.31 | COC1=C(C=C(C=C1)C2=COC3=CC(=C(C=C3C2=O)OC)O)O | Odoratin |
| GC72 | 8554 | C10H10O4 | 194.2 | COC(=O)C1=CC=CC=C1C(=O)OC | Mipax |
| GC73 | 64945 | C30H48O3 | 456.78 | CC1CCC2(CCC3(C(=CCC4C3(CCC5C4(CCC(C5(C)C)O)C)C)C2C1C)C)C(=O)O | ursolic acid |
| GC74 | 6782 | C16H22O4 | 278.38 | CC(C)COC(=O)C1=CC=CC=C1C(=O)OCC(C)C | DIBP |
| GC75 | 165675 | C10H20O | 156.3 | CC1CCC(C(C1)O)C(C)C | ()-Menthol |
| GC76 | 3026 | C16H22O4 | 278.38 | CCCCOC(=O)C1=CC=CC=C1C(=O)OCCCC | DBP |
| GC77 | 5317479 | C21H20O6 | 368.41 | CC(=CCC1=C(C=C2C(=C1O)C(=O)C(=CO2)C3=CC(=C(C=C3)OC)O)O)C | Gancaonin B |
| GC78 | 5317756 | C21H20O6 | 368.41 | CC(=CCC1=C(C=C2C(=C1OC)C=C(C(=O)O2)C3=C(C=C(C=C3)O)O)O)C | glycycoumarin |
| GC79 | 5318679 | C16H10O6 | 298.26 | COC1=C2C(=CC(=C1)O)OC(=O)C3=C2OC4=C3C=CC(=C4)O | Isotrifoliol |
| GC80 | 5319013 | C20H18O6 | 382.44 | CC(=CCC1=C(C=C(C(=C1OC)C2=COC3=C(C2=O)C=CC(=C3)O)O)OC)C | licoricone |
| GC81 | 5320083 | C21H18O6 | 366.39 | CC(=CCC1=C(C=C2C(=C1OC)C3=C(C4=C(O3)C=C(C=C4)O)C(=O)O2)O)C | glycyrol |
| GC82 | 746449 | C18H14O4 | 280.27 | CC(=O)OC1=CC2=C(C=C1)C(=O)C(=CO2)C3=CC=CC=C3 | glazarin |
| GC83 | 5317777 | C16H10O6 | 298.25 | C1OC2=C(O1)C(=C(C=C2)C3=COC4=C(C3=O)C=CC(=C4)O)O | glyzaglabrin |
| GC84 | 90479675 | C30H44O4 | 468.74 | CC1(C2CCC3(C(C2(CCC1O)C)C(=O)C=C4C3(CCC5(C4CC6(CC5OC6=O)C)C)C)C)C | glabrolide |
| GC85 | 5280378 | C16H12O4 | 268.28 | COC1=CC=C(C=C1)C2=COC3=C(C2=O)C=CC(=C3)O | formononetin |
| GC86 | 5319799 | C8H10N2O | 150.18 | CC1=CN2CCCC2=NC1=O | 3-methyl-6,7,8-trihydropyrrolo[1,2-a]pyrimidin-3-one |
| GC87 | 5319666 | C22H20O6 | 380.4 | CC(=CCC1=C(C=C2C(=C1OC)C3=C(C4=C(O3)C=C(C=C4)O)C(=O)O2)OC)C | 3-O-methylglycyrol |
| GC88 | 5321849 | C11H15N | 161.24 | CC1=CC(=NC2=C1CCCC2)C | 5,6,7,8-tetrahydro-2,4-dimethylquinoline |
| GC89 | 11378967 | C12H10O4 | 218.2 | CC1=CC(=O)OC2=C1C(=C(C=C2)C(=O)C)O | liqcoumarin |
