# Supplementary material for: Mechanisms of Ganweikang Tablets against Chronic Hepatitis B: A Comprehensive Study of Network Analysis, Molecular Docking, and Chemical Profiling
Source: Biomed Res Int. 2023 May 8;2023:8782892. doi: 10.1155/2023/8782892 (PMC10185428; doi:10.1155/2023/8782892)
Supplement: Supplementary Materials — Figure S1: schematic diagrams for the binding modes between targets and positive control small molecules. Figure S2: the binding patterns between active ingredients, positive control, and targets. Table S1: DEG results and disease-related targets. Table S2: compound-related targets. Table S3: KEGG pathway enrichment results on each module in TPT network. Table S4: detail information of CTP network. Table S5: molecular docking results of key targets. Table S6: detail information of UPLC-QTOF/MS analysis. Table S7: detail information of GC/MS analysis. Table S8: detail information of key active ingredients. Table S9: detail information of key targets. [file 8782892.f1.zip › Supplementary tables S3-S7.docx]

Mechanisms of Ganweikang Tablets against Chronic Hepatitis B: A Comprehensive Study of Network Analysis, Molecular Docking and Chemical Profiling

**Jia-Qi Xu^1,2^, Shi-Bing Su^3^, CY Chen^4,6^, J Gao^6,7^, ZM Cao^4,6^, JL Guan^5,6^, Lin-Xuan Xiao^1^, Mingming Zhao^1^, Hua Yu^1*^, Yuan-Jia Hu^1,2*^**

**Table S3-S7:**

Table S3 KEGG pathway enrichmentment results on each module in TPT network

| Pathway | Modularity | GeneRatio | Bgratio | p-value | p-adjust value | q-value | Count |
| --- | --- | --- | --- | --- | --- | --- | --- |
| Morphine addiction | 1 | 13/170 | 91/8104 | 4.45644389335974E-8 | 1.48176759454211E-6 | 8.97152520636894E-7 | 13 |
| Chemical carcinogenesis - reactive oxygen species | 2 | 20/170 | 223/8104 | 3.59165163041742E-8 | 1.48176759454211E-6 | 8.97152520636894E-7 | 20 |
| PD-L1 expression and PD-1 checkpoint pathway in cancer | 2 | 13/170 | 89/8104 | 3.38919858844808E-8 | 1.48176759454211E-6 | 8.97152520636894E-7 | 13 |
| Arachidonic acid metabolism | 3 | 11/170 | 61/8104 | 4.2222171968538E-8 | 1.48176759454211E-6 | 8.97152520636894E-7 | 11 |
| Endocrine resistance | 2 | 9/170 | 98/8104 | 2.02167353722897E-4 | 0.00141517147606028 | 8.56831166748014E-4 | 9 |
| Leishmaniasis | 2 | 8/170 | 77/8104 | 1.9527606522968E-4 | 0.00140387657705662 | 8.49992545665463E-4 | 8 |
| Folate biosynthesis | 3 | 5/170 | 26/8104 | 1.76224585916289E-4 | 0.00130210388482591 | 7.88373147520241E-4 | 5 |
| Measles | 2 | 11/170 | 139/8104 | 1.53510683398482E-4 | 0.00116668119382846 | 7.06379986765945E-4 | 11 |
| Estrogen signaling pathway | 2 | 13/170 | 138/8104 | 5.83155576650399E-6 | 1.10799559563576E-4 | 6.70848144567752E-5 | 13 |
| Yersinia infection | 2 | 11/170 | 137/8104 | 1.3488432971239E-4 | 0.00105527152069105 | 6.38925772321848E-4 | 11 |
| Epithelial cell signaling in Helicobacter pylori infection | 2 | 8/170 | 70/8104 | 9.92200878000773E-5 | 7.99774041055169E-4 | 4.84232007445353E-4 | 8 |
| Amphetamine addiction | 2 | 8/170 | 69/8104 | 8.94582006663238E-5 | 7.43621293038817E-4 | 4.50233707300906E-4 | 8 |
| Chemical carcinogenesis - DNA adducts | 3 | 8/170 | 69/8104 | 8.94582006663238E-5 | 7.43621293038817E-4 | 4.50233707300906E-4 | 8 |
| Arginine and proline metabolism | 3 | 7/170 | 51/8104 | 8.40321820685324E-5 | 7.43621293038817E-4 | 4.50233707300906E-4 | 7 |
| Nitrogen metabolism | 5 | 12/170 | 17/8104 | 2.77999282101289E-17 | 7.39478090389428E-15 | 4.47725159594707E-15 | 12 |
| Cocaine addiction | 2 | 7/170 | 49/8104 | 6.45850707296834E-5 | 5.9240099358951E-4 | 3.58675710404413E-4 | 7 |
| Retrograde endocannabinoid signaling | 1 | 12/170 | 148/8104 | 6.05794848880643E-5 | 5.75505106436611E-4 | 3.48445909318565E-4 | 12 |
| C-type lectin receptor signaling pathway | 2 | 10/170 | 104/8104 | 6.03948390870676E-5 | 5.75505106436611E-4 | 3.48445909318565E-4 | 10 |
| Relaxin signaling pathway | 2 | 13/170 | 129/8104 | 2.74067823748042E-6 | 5.6078493166907E-5 | 3.39533417274902E-5 | 13 |
| Kaposi sarcoma-associated herpesvirus infection | 2 | 14/170 | 194/8104 | 5.34940812389824E-5 | 5.4728560036805E-4 | 3.31360098362927E-4 | 14 |
| B cell receptor signaling pathway | 2 | 9/170 | 82/8104 | 5.01481169189033E-5 | 5.33575964017131E-4 | 3.23059447940724E-4 | 9 |
| AGE-RAGE signaling pathway in diabetic complications | 3 | 10/170 | 100/8104 | 4.30494862118204E-5 | 4.77131805514343E-4 | 2.88884710105637E-4 | 10 |
| Nicotine addiction | 1 | 11/170 | 40/8104 | 3.41838653021025E-10 | 4.54645408517963E-8 | 2.75270073222194E-8 | 11 |
| Chemical carcinogenesis - receptor activation | 2 | 17/170 | 212/8104 | 1.96581971704785E-6 | 4.53032977762418E-5 | 2.74293809250693E-5 | 17 |
| Tryptophan metabolism | 3 | 8/170 | 42/8104 | 2.04375779441693E-6 | 4.53032977762418E-5 | 2.74293809250693E-5 | 8 |
| Antifolate resistance | 3 | 6/170 | 31/8104 | 3.71623295888261E-5 | 4.29790420462075E-4 | 2.60221346777592E-4 | 6 |
| Galactose metabolism | 3 | 6/170 | 31/8104 | 3.71623295888261E-5 | 4.29790420462075E-4 | 2.60221346777592E-4 | 6 |
| Prostate cancer | 2 | 10/170 | 97/8104 | 3.30156132912157E-5 | 4.18197768355399E-4 | 2.53202447797294E-4 | 10 |
| IL-17 signaling pathway | 2 | 10/170 | 94/8104 | 2.50549197706775E-5 | 3.33230432950011E-4 | 2.0175803815335E-4 | 10 |
| Serotonergic synapse | 3 | 14/170 | 115/8104 | 1.05724627391988E-7 | 3.12475009847431E-6 | 1.89191438490926E-6 | 14 |
| GABAergic synapse | 1 | 14/170 | 89/8104 | 3.63884515842421E-9 | 2.4198320303521E-7 | 1.46511397168133E-7 | 14 |
| Steroid hormone biosynthesis | 3 | 12/170 | 61/8104 | 3.51559657990863E-9 | 2.4198320303521E-7 | 1.46511397168133E-7 | 12 |
| Ovarian steroidogenesis | 3 | 9/170 | 51/8104 | 9.08710825360739E-7 | 2.41717079545957E-5 | 1.46350269768624E-5 | 9 |
| Insulin resistance | 2 | 11/170 | 108/8104 | 1.485620373735E-5 | 2.079868523229E-4 | 1.25927931956484E-4 | 11 |
| Th17 cell differentiation | 2 | 11/170 | 108/8104 | 1.485620373735E-5 | 2.079868523229E-4 | 1.25927931956484E-4 | 11 |
| Prolactin signaling pathway | 2 | 9/170 | 70/8104 | 1.38035753797341E-5 | 2.079868523229E-4 | 1.25927931956484E-4 | 9 |
| Lipid and atherosclerosis | 2 | 16/170 | 215/8104 | 1.04349042214674E-5 | 1.73480282681896E-4 | 1.0503554907135E-4 | 16 |
| T cell receptor signaling pathway | 2 | 11/170 | 104/8104 | 1.03269009205227E-5 | 1.73480282681896E-4 | 1.0503554907135E-4 | 11 |
| Influenza A | 2 | 8/170 | 171/8104 | 0.0272690104648493 | 0.076353229301578 | 0.0462289041675561 | 8 |
| Ras signaling pathway | 2 | 10/170 | 232/8104 | 0.0239783164023086 | 0.0681886910405629 | 0.0412855944962648 | 10 |
| Apoptosis | 2 | 7/170 | 136/8104 | 0.0240967554804997 | 0.0681886910405629 | 0.0412855944962648 | 7 |
| Human papillomavirus infection | 2 | 13/170 | 331/8104 | 0.0216833747134256 | 0.0626932355844697 | 0.0379583104251044 | 13 |
| MAPK signaling pathway | 2 | 12/170 | 294/8104 | 0.0206512413873866 | 0.0609301237559842 | 0.0368908149373391 | 12 |
| JAK-STAT signaling pathway | 2 | 8/170 | 162/8104 | 0.0205014771544625 | 0.0609301237559842 | 0.0368908149373391 | 8 |
| Dopaminergic synapse | 2 | 7/170 | 132/8104 | 0.0208445160217841 | 0.0609301237559842 | 0.0368908149373391 | 7 |
| cAMP signaling pathway | 2 | 10/170 | 219/8104 | 0.016761617888089 | 0.05066579952536 | 0.0306761667090624 | 10 |
| Non-alcoholic fatty liver disease | 2 | 8/170 | 155/8104 | 0.0161382315510175 | 0.04934217922495 | 0.029874766210595 | 8 |
| Salmonella infection | 2 | 11/170 | 249/8104 | 0.0155303889518042 | 0.0480358541997665 | 0.0290838373271242 | 11 |
| Alzheimer disease | 2 | 15/170 | 384/8104 | 0.0147686501119364 | 0.0462171874091187 | 0.027982705475248 | 15 |
| Adipocytokine signaling pathway | 2 | 5/170 | 69/8104 | 0.0146146226817116 | 0.0462171874091187 | 0.027982705475248 | 5 |
| MicroRNAs in cancer | 3 | 13/170 | 310/8104 | 0.0132789041159334 | 0.0425564878896179 | 0.0257662946066939 | 13 |
| Growth hormone synthesis, secretion and action | 2 | 7/170 | 119/8104 | 0.0123994772888363 | 0.0402226946198835 | 0.0243532737508594 | 7 |
| Neurotrophin signaling pathway | 2 | 7/170 | 119/8104 | 0.0123994772888363 | 0.0402226946198835 | 0.0243532737508594 | 7 |
| Bile secretion | 3 | 6/170 | 89/8104 | 0.0108006091811253 | 0.0359120255272416 | 0.0217433316409496 | 6 |
| Arginine biosynthesis | 2 | 3/170 | 22/8104 | 0.0104180591227732 | 0.0350785281855402 | 0.0212386814894644 | 3 |
| Epstein-Barr virus infection | 2 | 10/170 | 202/8104 | 0.00992845664121739 | 0.0338585829046644 | 0.0205000521741736 | 10 |
| Alcoholic liver disease | 2 | 8/170 | 142/8104 | 0.00988188068936601 | 0.0338585829046644 | 0.0205000521741736 | 8 |
| Glycerolipid metabolism | 3 | 5/170 | 61/8104 | 0.00883941333529992 | 0.0309379466735497 | 0.0187317207797907 | 5 |
| Pathogenic Escherichia coli infection | 2 | 10/170 | 197/8104 | 0.00839782056042325 | 0.0297842702543011 | 0.0180332146771194 | 10 |
| Starch and sucrose metabolism | 3 | 4/170 | 36/8104 | 0.00652421556931483 | 0.023451910019429 | 0.0141992173841418 | 4 |
| NF-kappa B signaling pathway | 2 | 7/170 | 104/8104 | 0.00609848248776877 | 0.0223302503256075 | 0.0135200961607359 | 7 |
| EGFR tyrosine kinase inhibitor resistance | 2 | 6/170 | 79/8104 | 0.00612822659311785 | 0.0223302503256075 | 0.0135200961607359 | 6 |
| Metabolism of xenobiotics by cytochrome P450 | 3 | 6/170 | 78/8104 | 0.00576052673526665 | 0.0215816917124075 | 0.0130668730985293 | 6 |
| Pancreatic cancer | 2 | 6/170 | 76/8104 | 0.00507387177056171 | 0.0192807127281345 | 0.0116737200134728 | 6 |
| Pertussis | 2 | 6/170 | 76/8104 | 0.00507387177056171 | 0.0192807127281345 | 0.0116737200134728 | 6 |
| Choline metabolism in cancer | 2 | 7/170 | 98/8104 | 0.00440831347522204 | 0.0172442850648392 | 0.0104407424413154 | 7 |
| Proteoglycans in cancer | 2 | 11/170 | 205/8104 | 0.00383149578190038 | 0.015417895393928 | 0.00933493468647008 | 11 |
| Drug metabolism - cytochrome P450 | 3 | 6/170 | 72/8104 | 0.00388345485486157 | 0.015417895393928 | 0.00933493468647008 | 6 |
| Breast cancer | 2 | 9/170 | 147/8104 | 0.00368891614084178 | 0.0150961798994448 | 0.00914014849471728 | 9 |
| Coronavirus disease - COVID-19 | 2 | 12/170 | 232/8104 | 0.00345160118060508 | 0.0143457174068898 | 0.00868577270777264 | 12 |
| Renal cell carcinoma | 2 | 6/170 | 69/8104 | 0.00313710339345667 | 0.0132455476612615 | 0.00801966281034036 | 6 |
| Toxoplasmosis | 3 | 8/170 | 112/8104 | 0.00237416403188554 | 0.0101859295561541 | 0.00616718330863307 | 8 |
| Inflammatory bowel disease | 2 | 6/170 | 65/8104 | 0.00231536722984007 | 0.010096519395696 | 0.00611304894159674 | 6 |
| Taste transduction | 1 | 7/170 | 86/8104 | 0.00211060144044253 | 0.00935699971929522 | 0.00566529860329311 | 7 |
| Hepatitis C | 2 | 10/170 | 157/8104 | 0.00167003092463605 | 0.00752929196530827 | 0.00455869280052302 | 10 |
| Bladder cancer | 2 | 5/170 | 41/8104 | 0.00155756917258745 | 0.00714333448117692 | 0.00432501058812849 | 5 |
| Toll-like receptor signaling pathway | 2 | 8/170 | 104/8104 | 0.0014812778592902 | 0.00691263001002093 | 0.00418532802347923 | 8 |
| Osteoclast differentiation | 2 | 9/170 | 128/8104 | 0.00143308429098478 | 0.00680715038217769 | 0.00412146422031336 | 9 |
| Chronic myeloid leukemia | 2 | 7/170 | 76/8104 | 0.0010228409138793 | 0.0049468306016708 | 0.00299511310667049 | 7 |
| Histidine metabolism | 3 | 4/170 | 22/8104 | 0.00101752398743811 | 0.0049468306016708 | 0.00299511310667049 | 4 |
| Thyroid hormone signaling pathway | 2 | 9/170 | 121/8104 | 9.6216646061387E-4 | 0.00482898638723187 | 0.00292376302828048 | 9 |
| Human T-cell leukemia virus 1 infection | 2 | 13/170 | 222/8104 | 7.66820954709593E-4 | 0.00392258411447599 | 0.00237497178280501 | 13 |
| Adherens junction | 2 | 7/170 | 71/8104 | 6.79179416445827E-4 | 0.00354238676028608 | 0.00214477710456577 | 7 |
| Fluid shear stress and atherosclerosis | 2 | 10/170 | 139/8104 | 6.56574910108308E-4 | 0.00352159086355539 | 0.00213218599969915 | 10 |
| Small cell lung cancer | 2 | 8/170 | 92/8104 | 6.61953169841238E-4 | 0.00352159086355539 | 0.00213218599969915 | 8 |
| Th1 and Th2 cell differentiation | 2 | 8/170 | 92/8104 | 6.61953169841238E-4 | 0.00352159086355539 | 0.00213218599969915 | 8 |
| Hepatitis B | 2 | 11/170 | 162/8104 | 5.79205674011153E-4 | 0.00327805764440355 | 0.00198473612819051 | 11 |
| TNF signaling pathway | 2 | 9/170 | 112/8104 | 5.49547004602251E-4 | 0.00317781528748258 | 0.00192404328842436 | 9 |
| HIF-1 signaling pathway | 2 | 9/170 | 109/8104 | 4.49894197181866E-4 | 0.00268635283836089 | 0.00162648193220901 | 9 |
| Other glycan degradation | 4 | 4/170 | 18/8104 | 4.54458186940752E-4 | 0.00268635283836089 | 0.00162648193220901 | 4 |
| Neuroactive ligand-receptor interaction | 1 | 18/170 | 350/8104 | 3.75211146785429E-4 | 0.00232107360569591 | 0.00140531959505926 | 18 |
| GnRH secretion | 1 | 7/170 | 64/8104 | 3.58723178757263E-4 | 0.00227191346546267 | 0.00137555504636244 | 7 |
| Viral carcinogenesis | 2 | 13/170 | 204/8104 | 3.42907850481187E-4 | 0.00222471922507306 | 0.00134698077339208 | 13 |
| Linoleic acid metabolism | 3 | 5/170 | 29/8104 | 3.02355212406303E-4 | 0.00201066216250191 | 0.00121737756574117 | 5 |
| Chagas disease | 2 | 9/170 | 102/8104 | 2.73921662599641E-4 | 0.00186828621157704 | 0.00113117447713212 | 9 |

Table S4 Detail information of CTP network

| ID | Name | ID | Name |
| --- | --- | --- | --- |
| HQ01 | CA2 | YCH02 | ABCG2 |
| HQ01 | CA6 | YCH02 | CA1 |
| HQ01 | CA1 | YCH02 | CA6 |
| HQ01 | MMP9 | YCH02 | CA2 |
| HQ02 | CA2 | YCH02 | JUN |
| HQ02 | CA6 | YCH02 | GLO1 |
| HQ02 | CA1 | YCH02 | IKBKG |
| HQ02 | EP300 | YCH02 | NFKB1 |
| HQ02 | FOS | YCH02 | STAT1 |
| HQ02 | JUN | BZ01 | GPBAR1 |
| HQ02 | GLO1 | GC01 | GBA |
| HQ02 | MMP9 | GC02 | CA1 |
| HQ02 | IKBKG | GC02 | CA6 |
| HQ02 | NFKB1 | GC03 | ABCG2 |
| HQ03 | SIRT2 | GC04 | CA6 |
| HQ04 | ABCG2 | GC05 | CA1 |
| HQ04 | ABCB1 | GC06 | CA6 |
| HQ05 | ABCB1 | GC06 | CA1 |
| HQ05 | RELA | GC06 | CA2 |
| HQ06 | ABCB1 | GC07 | CD81 |
| HQ07 | GABBR1 | GC07 | DUSP3 |
| HQ07 | GABBR2 | GC07 | GPBAR1 |
| A01 | CA1 | GC07 | PTPRC |
| A01 | CA6 | GC07 | RELA |
| A01 | CA2 | GC08 | ABCB1 |
| B01 | AKR1B1 | GC09 | RELA |
| B01 | CA6 | GC10 | ABCB1 |
| B01 | CA1 | GC10 | PDE4D |
| B01 | CA2 | GC11 | PDE4D |
| B01 | DPP4 | GC12 | CA1 |
| B01 | MMP9 | GC12 | CA2 |
| C01 | RORC | GC12 | CES1 |
| H01 | RORC | GC12 | FEN1 |
| MBC01 | CD81 | GC13 | CD81 |
| MBC01 | DUSP3 | GC13 | GPBAR1 |
| MBC01 | GPBAR1 | GC13 | RELA |
| MBC01 | PTPRC | GC14 | PDE4D |
| MBC01 | RELA | GC15 | AKT1 |
| MBC02 | GPBAR1 | GC15 | PDE4D |
| MBC02 | RELA | GC16 | ABCG2 |
| MBC03 | ABCG2 | GC16 | AKR1B1 |
| MBC03 | ABCB1 | GC16 | ELAVL1 |
| L01 | CD81 | GC16 | GLO1 |
| L01 | GPBAR1 | GC16 | PDE4D |
| L01 | RORC | GC17 | ABCG2 |
| L01 | RELA | GC17 | AKR1B1 |
| D01 | RORC | GC17 | NLRP3 |
| LQ01 | RELA | GC18 | ABCB1 |
| LQ02 | CA6 | GC18 | STAT1 |
| LQ02 | CA1 | GC19 | ABCB1 |
| LQ03 | GPBAR1 | GC20 | CA2 |
| LQ03 | RELA | GC21 | CD81 |
| E01 | CD81 | GC21 | GPBAR1 |
| E01 | GPBAR1 | GC21 | RORC |
| E01 | RELA | GC21 | RELA |
| E02 | CD81 | GC22 | STAT1 |
| E02 | DUSP3 | GC23 | ABCB1 |
| E02 | GPBAR1 | GC24 | PDE4D |
| E02 | PTPRC | AKT1 | Hepatitis B |
| E02 | RELA | AKT1 | Toll-like receptor signaling pathway |
| G01 | CREB1 | AKT1 | MAPK signaling pathway |
| G01 | ABCB1 | DUSP3 | MAPK signaling pathway |
| FF01 | CA1 | STAT1 | Hepatitis B |
| FF02 | PDE4D | STAT1 | Toll-like receptor signaling pathway |
| FF02 | SRD5A1 | RELA | Hepatitis B |
| FF03 | CES1 | RELA | Toll-like receptor signaling pathway |
| FF04 | KCNA3 | RELA | MAPK signaling pathway |
| FF05 | CA1 | RELA | NF-kappa B signaling pathway |
| FF06 | CA1 | CREB1 | Hepatitis B |
| FF07 | KCNA3 | JUN | Hepatitis B |
| FF08 | CA2 | JUN | Toll-like receptor signaling pathway |
| FF09 | NFKB1 | JUN | MAPK signaling pathway |
| K01 | CA6 | IKBKG | Hepatitis B |
| K01 | CA1 | IKBKG | Toll-like receptor signaling pathway |
| HX01 | ABCB1 | IKBKG | MAPK signaling pathway |
| HX02 | CES1 | IKBKG | NF-kappa B signaling pathway |
| HX03 | CD81 | NFKB1 | Hepatitis B |
| HX03 | GPBAR1 | NFKB1 | Toll-like receptor signaling pathway |
| HX03 | RELA | NFKB1 | MAPK signaling pathway |
| HX04 | CDK2 | NFKB1 | NF-kappa B signaling pathway |
| HX05 | CES1 | CDK2 | Hepatitis B |
| HX06 | RELA | EP300 | Hepatitis B |
| HX07 | CA2 | FOS | Hepatitis B |
| HX08 | RELA | FOS | Toll-like receptor signaling pathway |
| YCH01 | CA1 | FOS | MAPK signaling pathway |
| YCH01 | CA6 | MMP9 | Hepatitis B |
| YCH01 | EP300 | AKT1 | JAK-STAT signaling pathway |
| YCH01 | FOS | STAT1 | JAK-STAT signaling pathway |
| YCH01 | GLO1 | EP300 | JAK-STAT signaling pathway |
| YCH01 | NFKB1 | / | / |

Table S5 Molecular docking results of key targets

| Target | PDB ID | Method | Resolution | Herb No. | Compound name | Pubchem CID | Affinity (kcal/mol) |
| --- | --- | --- | --- | --- | --- | --- | --- |
| CA2 | 3DC3 | X-RAY DIFFRACTION | 1.70 Å | HQ01 | isoferulic acid | 736186 | -5.12 |
|  |  |  |  | HQ02 | Cis-ferulic acid | 1548883 | -5.51 |
|  |  |  |  | A01 | vanillic acid | 8468 | -4.85 |
|  |  |  |  | B01 | Caffeate | 1549111 | -5.7 |
|  |  |  |  | HX07 | anisaldehyde | 11252044 | -4.75 |
|  |  |  |  | YCH02 | Dimethylcaffeic acid | 717531 | -5.15 |
|  |  |  |  | GC06 | protocatechuic acid | 72 | -5.62 |
|  |  |  |  | GC12 | Karenzu DK2 | 8433 | -6.33 |
|  |  |  |  | GC20 | Mipax | 8554 | -5.02 |
|  |  |  |  | positive control | AZM |  | -5.86 |
| EP300 | 4PZS | X-RAY DIFFRACTION | 1.94 Å | HQ02 | Cis-ferulic acid | 1548883 | -5.47 |
|  |  |  |  | YCH01 | Vanillin | 1183 | -6.26 |
|  |  |  |  | positive control | ACO |  | -4.7 |
| CDK2 | 2IW8 | X-RAY DIFFRACTION | 2.30 Å | HX04 | 3,4-methylenedioxy-10-hydroxy aristololactam | 5319620 | -7.99 |
|  |  |  |  | positive control | 4SP |  | -7.58 |
| ABCB1 | 6UJN | X-RAY DIFFRACTION | 3.98 Å | G01 | wogonin | 5281703 | -5.9 |
|  |  |  |  | MBC03 | artemisetin | 5320351 | -5.27 |
|  |  |  |  | HQ06 | formononetin | 10378473 | -7.19 |
|  |  |  |  | GC08 | Castanin | 5281704 | -6.53 |
|  |  |  |  | GC18 | artemisetin | 136419 | -7.26 |
|  |  |  |  | GC19 | Odoratin | 13965473 | -6.01 |
|  |  |  |  | HQ04 | 5,3'-Dihydroxy-7,4'-dimethoxyisoflavone | 44257336 | -6.27 |
|  |  |  |  | HQ05 | 3,5-dimethoxystilbene | 5316874 | -6.05 |
|  |  |  |  | HX01 | irisolidone | 5281781 | -6.28 |
|  |  |  |  | positive control | Tepotinib |  | -6.5 |
| AKT1 | 6HHG | X-RAY DIFFRACTION | 2.30 Å | GC15 | 2-(3,4-dihydroxyphenyl)-5,7-dihydroxy-6-(3-methylbut-2-enyl)chromone | 14604081 | -8.8 |
|  |  |  |  | positive control | G4T |  | -9.01 |
| JUN | 5FV8 | X-RAY DIFFRACTION | 1.99 Å | HQ02 | Cis-ferulic acid | 1548883 | -4.25 |
|  |  |  |  | YCH02 | Dimethylcaffeic acid | 717531 | -4.23 |
|  |  |  |  | positive control | JNK-IN-8 |  | -6.01 |
| CA1 | 1AZM | X-RAY DIFFRACTION | 2.00 Å | K01 | scopoletin | 5280460 | -5.59 |
|  |  |  |  | FF01 | fraxidin | 3083616 | -5.94 |
|  |  |  |  | FF05 | fraxetin | 5273569 | -5.89 |
|  |  |  |  | FF06 | Phytodolor | 5318565 | -5.45 |
|  |  |  |  | GC02 | ferulic acid | 54691413 | -5.08 |
|  |  |  |  | GC05 | 7-methoxy-2-methylisoflavone | 354368 | -7.18 |
|  |  |  |  | positive control | AZM |  | -5.7 |
| CA6 | 3FE4 | X-RAY DIFFRACTION | 1.90 Å | K01 | scopoletin | 5280460 | -5.81 |
|  |  |  |  | GC04 | sinapic acid | 54710960 | -5.63 |
|  |  |  |  | positive control | Zonisamide |  | -8 |
| FOS | 1A02 | X-RAY DIFFRACTION | 2.70 Å | HQ02 | Cis-ferulic acid | 1548883 | -4.39 |
|  |  |  |  | YCH01 | Vanillin | 1183 | -2.91 |
|  |  |  |  | positive control | T-5224 |  | -4.83 |
| ABCG2 | 6VXI | ELECTRON MICROSCOPY | 3.70 Å | MBC03 | artemisetin | 5320351 | -3.92 |
|  |  |  |  | GC03 | gancaonin p-3'-methylether | 5317483 | -4.84 |
|  |  |  |  | GC16 | Gancaonin P | 5481966 | -3.87 |
|  |  |  |  | GC17 | (Z)-1-(2,4-dihydroxyphenyl)-3-phenylprop-2-en-1-one | 10331849 | -4.97 |
|  |  |  |  | HQ04 | 5,3'-Dihydroxy-7,4'-dimethoxyisoflavone | 44257336 | -4.91 |
|  |  |  |  | YCH02 | Dimethylcaffeic acid | 717531 | -4.74 |
|  |  |  |  | positive control | MIX |  | -6.09 |
| MMP9 | 5UE4 | X-RAY DIFFRACTION | 1.80 Å | HQ01 | isoferulic acid | 736186 | -6.67 |
|  |  |  |  | HQ02 | Cis-ferulic acid | 1548883 | -5.73 |
|  |  |  |  | B01 | Caffeate | 1549111 | -6.32 |
|  |  |  |  | positive control | 5XQ |  | -7.19 |
| CREB1 | 5ZKO | X-RAY DIFFRACTION | 3.05 Å | G01 | wogonin | 5281703 | -3.67 |
|  |  |  |  | positive control | 666-15 |  | -2.03 |
| STAT1 | 1YVL | X-RAY DIFFRACTION | 3.00 Å | YCH02 | Dimethylcaffeic acid | 717531 | -5.58 |
|  |  |  |  | GC18 | Daidzein dimethyl ether | 136419 | -4.56 |
|  |  |  |  | GC22 | glazarin | 746449 | -5.42 |
|  |  |  |  | positive control | Fludarabine |  | -2.21 |
| NFKB1 | 1SVC | X-RAY DIFFRACTION | 2.60 Å | HQ02 | Cis-ferulic acid | 1548883 | -4.46 |
|  |  |  |  | FF09 | psoralen | 6199 | -6.11 |
|  |  |  |  | YCH01 | Vanillin | 1183 | -4.29 |
|  |  |  |  | YCH02 | Dimethylcaffeic acid | 717531 | -4.97 |
|  |  |  |  | positive control | Tectochrysin |  | -6.23 |
| RELA | 1NFI | X-RAY DIFFRACTION | 2.70 Å | HQ05 | 3,5-dimethoxystilbene | 5316874 | -5.02 |
|  |  |  |  | MBC01 | 3-Epioleanolic acid | 11869658 | -6.78 |
|  |  |  |  | MBC02 | Tormentic acid | 73193 | -6.29 |
|  |  |  |  | LQ01 | beta-Amyrenyl acetate | 345510 | -7.56 |
|  |  |  |  | LQ03 | ursolic acid | 49867942 | -7.58 |
|  |  |  |  | E01 | oleanolic acid | 49867939 | -6.87 |
|  |  |  |  | HX03 | 2a,3ÃŸ-dihydroxyolean-12-en-28-oicacid | 7163260 | -6.54 |
|  |  |  |  | HX08 | p-methoxycinnamaldehyde | 641294 | -4.24 |
|  |  |  |  | GC09 | anethole | 637563 | -4.12 |
|  |  |  |  | GC13 | 11-deoxyglycyrrhetic acid | 12305517 | -6.8 |
|  |  |  |  | GC21 | ursolic acid | 64945 | -6.9 |
|  |  |  |  | positive control | Dihydroartemisinin |  | -5.19 |
| IKBKG | 4OWF | X-RAY DIFFRACTION | 2.00 Å | HQ02 | Cis-ferulic acid | 1548883 | -3.59 |
|  |  |  |  | YCH02 | Dimethylcaffeic acid | 717531 | -4.06 |
|  |  |  |  | positive control | / |  |  |

Table S6 Detail information of UPLC-QTOF/MS analysis

| No. | Retention Time (min) | Negative ion mode | Positive ion mode | Identification | Molecular Formula |
| --- | --- | --- | --- | --- | --- |
| 1 | 1.35 | 191.1568[M-H]-, 111.0086 | 193.0256[M+H]+ | scopoletin | C_10_H_8_O_4_ |
| 2 | 3.43 | 375.1288[M-H]-, 213.0756 | 399.1238[M+NA]+ | Adoxosidicacid | C_16_H_24_O_10_ |
| 3 | 4.258 | 461.1646[M-H]- | 485.1613[M+NA]+ |  | C_30_H_38_O_4_ |
| 4 | 4.258 | 461.1646[M-H]- | 485.1613[M+NA]+ | Pratensein 7-O-glucoside | C_22_H_22_O_11_ |
| 5 | 4.258 | 461.1646[M-H]- | 485.1613[M+NA]+ | Methylinissolin 3-O-glucoside | C_23_H_26_O_10_ |
| 6 | 6.62 | 433.1342[M+HCOO-]-, 161.0251 | 411.1229[M+NA]+, 177.0501 | artemisetin | C_20_H_20_O_8_ |
| 7 | 8.07 | 469.1690[M-H]-,163.0341 | - | beta-Amyrenyl acetate | C_32_H_52_O_2_ |
| 8 | 8.941 | 623.1995[M-H]-, 161.0222(Glu-H2O) | 647.1965[M+NA]+, 163.0341(Glu-H2O) | Forsythoside I | C_30_H_38_O_14_ |
| 9 | 9.383 | 623.1995[M-H]-, 161.0222(Glu-H2O) | 647.1965[M+NA]+,471.1483, 325.0881(2Glu-H2O), 163.0341(Glu-H2O) | Forsythoside A | C_29_H_36_O_15_ |
| 10 | 10.63 | 623.1995[M-H]-, 161.0222(Glu-H2O) | 647.1965[M+NA]+, 163.0341(Glu-H2O) | ForsythosideA isomer | C_29_H_36_O_15_ |
| 11 | 10.904 | 497.1655[M+HCOO-]- | 453.1732[M+H]+, 291.1193[M+H-C6H10O5]+, 243.0607 |  | C_22_H_28_O_10_ |
| 12 | 12.779 | - | 431.1305， 269.0764 | Ononin | C_22_H_22_O_9_ |
| 13 | 13.392 | - | 247.0916 |  | C_14_H_14_O_4_ |
| 14 | 13.812 | 579.2087[M+HCOO-]-, 371.1485[M-Glu]- | 557.1989[M+NA]+ | Luteolin-7-O-(6-O-malonyl-β-D-glucoside) | C_24_H_22_O_14_ |
| 15 | 14.596 | 283.0593[M-H]-, 211.0378 | 285.0722[M+H]+ , 213.0504 | wogonin | C_16_H_12_O_5_ |
| 16 | 14.739 | 283.0597 | 285.0721 | Calycosin | C_16_H_12_O_5_ |
| 17 | 15.187 | 491.1193[M+HCOO-]-, 283.0598[M-H-Glu]- | 447.1271[M+H]+ , 285.0724[M+H-Glu]+ | Calycosin 7-O-glucoside | C_22_H_22_O_10_ |
| 18 | 17.262 | - | 947.531[M+NA]+ | Astragaloside VII | C_47_H_78_O_19_ |
| 19 | 17.747 | - | 850.327[M+NA]+ | Astragaloside II | C_43_H_70_O_15_ |
| 20 | 19.16 | 267.0641 | 269.0767 | Formonentin | C_16_H_12_O_4_ |
| 21 | 19.315 | - | 883.4402， 451.3184 | Astragaloside I | C_46_H_74_O_16_ |
| 22 | 20.184 | - | 807.4574[M+NA]+ | Astragaloside IV | C_41_H_68_O_14_ |
| 23 | 20.847 | 821.4001[M-H]- | 823.4173[M+H]+ |  | C_42_H_62_O_16_ |
| 24 | 27.375 | 265.1461, 152.9934 | - | 7-methoxy-2-methylisoflavone | C_17_H_14_O_3_ |
| 25 | 32.443 | - | 301.1379[M+NA]+, 149.0186 | 3,4-methylenedioxy-10-hydroxy aristololactam | C_16_H_9_NO_4_ |

Table S7 Detail information of GC/MS analysis

| No. | Retention Time(min) | Identification | Molecular Formula | CAS # | RI | MW | AMOUNT(%) |
| --- | --- | --- | --- | --- | --- | --- | --- |
| 1 | 2.83 | 4-Methylheptane | C_8_H_18_ | 589-53-7 | 767 | 114.23 | 1.39 |
| 2 | 3.18 | n-Octane | C_8_H_18_ | 111-65-9 | 800 | 114.23 | 1.17 |
| 3 | 3.46 | 1.2,4-Dimethylheptane | C_9_H_20_ | 2213-23-2 | 821 | 128.25 | 0.54 |
| 4 | 3.67 | 2,4-Dimethyl-1-heptene | C_9_H_18_ | 19549-87-2 | 836 | 126.24 | 2.84 |
| 5 | 4.95 | 3-Thujene | C_10_H_16_ | 5/2/2867 | 929 | 136.23 | 1.07 |
| 6 | 5.08 | α-Pinene | C_10_H_16_ | 80-56-8 | 937 | 136.23 | 9.43 |
| 7 | 5.81 | Sabinene | C_10_H_16_ | 3387-41-5 | 974 | 136.23 | 2.62 |
| 8 | 5.91 | β-Pinene | C_10_H_16_ | 127-91-3 | 979 | 136.23 | 23.27 |
| 9 | 6.92 | o-Cymene | C_10_H_14_ | 527-84-4 | 1022 | 134.22 | 2 |
| 10 | 7.04 | 1.2,6-Dimethylnonane | C_11_H_24_ | 17302-28-2 | 1018 | 156.31 | 0.95 |
| 11 | 7.16 | β-Terpinyl acetate | C_12_H_20_O_2_ | 10198-23-9 | 1317 | 196.29 | 1.11 |
| 12 | 7.91 | γ-Terpinene | C_10_H_16_ | 99-85-4 | 1060 | 136.23 | 0.81 |
| 13 | 8.81 | (Z)-1,4-Dimethylcyclooctane | C_10_H_20_ | 13151-99-0 | 1054 | 140.27 | 0.48 |
| 14 | 11.04 | Terpinen-4-ol | C_10_H_18_O | 562-74-3 | 1177 | 154.25 | 0.76 |
| 15 | 13.42 | 1,3-Di-tert-butylbenzene | C_14_H_22_ | 1014-60-4 | 1247 | 190.32 | 1.73 |
| 16 | 13.63 | 4,6-Dimethyldodecane | C_14_H_30_ | 61141-72-8 | 1325 | 198.39 | 0.3 |
| 17 | 15.27 | 2-Isopropyl-5-methyl-1-heptanol | C_11_H_24_O | 91337-07-4 | - | 172.31 | 0.56 |
| 18 | 15.5 | (5E)-5-Icosene | C_20_H_40_ | 74685-30-6 | 2268 | 280.5 | 0.7 |
| 19 | 15.72 | 2-Hexyl-1-decanol | C_16_H_34_O | 2425-77-6 | 1504 | 242.44 | 0.51 |
| 20 | 19.9 | 2,4-di-t-Butylphenol | C_14_H_22_O | 96-76-4 | 1519 | 206.32 | 0.63 |
| 21 | 20.47 | 4βH,5α-Eremophila-1(10),11-diene | C_15_H_24_ | 7/3/4630 | 1492 | 204.35 | 0.73 |
| 22 | 20.79 | γ-Elemene | C_15_H_24_ | 29873-99-2 | 1433 | 204.35 | 0.96 |
| 23 | 20.98 | Trichloroacetic acid | C_18_H_33_Cl_3_O_2_ | 74339-54-1 | 2405 | 387.8 | 0.32 |
| 24 | 21.88 | Atractylon | C_15_H_20_O | 6989-21-5 | 1662 | 216.32 | 2.63 |
| 25 | 23.57 | 2,2-Dimethoxy-2-phenylacetophenone | C_16_H_16_O_3_ | 24650-42-8 | 1874 | 256.3 | 0.89 |
