# Supplementary material for: Mechanisms of Ganweikang Tablets against Chronic Hepatitis B: A Comprehensive Study of Network Analysis, Molecular Docking, and Chemical Profiling
Source: Biomed Res Int. 2023 May 8;2023:8782892. doi: 10.1155/2023/8782892 (PMC10185428; doi:10.1155/2023/8782892)
Supplement: Supplementary Materials — Figure S1: schematic diagrams for the binding modes between targets and positive control small molecules. Figure S2: the binding patterns between active ingredients, positive control, and targets. Table S1: DEG results and disease-related targets. Table S2: compound-related targets. Table S3: KEGG pathway enrichment results on each module in TPT network. Table S4: detail information of CTP network. Table S5: molecular docking results of key targets. Table S6: detail information of UPLC-QTOF/MS analysis. Table S7: detail information of GC/MS analysis. Table S8: detail information of key active ingredients. Table S9: detail information of key targets. [file 8782892.f1.zip › Table S8 Detail information of key active ingredients.docx]

Table S8 Detail information of key active ingredients

| Compound | CAS | Formular | MW | CanonicalSMILES | Heavy atoms | Aromatic heavy atoms | Fraction Csp3 | Rotatable bonds | H-bond acceptors | H-bond donors | MR | TPSA | iLOGP | XLOGP3 | WLOGP | MLOGP | Silicos-IT Log P | Consensus LogP | log Kp (cm/s) | Lipinski violations |
| --- | --- | --- | --- | --- | --- | --- | --- | --- | --- | --- | --- | --- | --- | --- | --- | --- | --- | --- | --- | --- |
| ferulic acid | 25522-33-2 | C10H10O4 | 194.2 | COC1=C(C=C(C=C1)/C=C/C(=O)O)O | 14 | 6 | 0.1 | 3 | 4 | 2 | 51.63 | 66.76 | 1.79 | 1.51 | 1.39 | 1 | 1.26 | 1.39 | -6.41 | 0 |
| oleanolic acid | [508-02-1](https://commonchemistry.cas.org/detail?cas_rn=508-02-1) | C30H48O3 | 456.78 | CC1(CCC2(CCC3(C(=CCC4C3(CCC5C4(CCC(C5(C)C)O)C)C)C2C1)C)C(=O)O)C | 33 | 0 | 0.9 | 1 | 3 | 2 | 136.65 | 57.53 | 3.89 | 7.49 | 7.23 | 5.82 | 5.85 | 6.06 | -3.77 | 1 |
| ursolic acid | 77-52-1 | C30H48O3 | 456.78 | CC1CCC2(CCC3(C(=CCC4C3(CCC5C4(CCC(C5(C)C)O)C)C)C2C1C)C)C(=O)O | 33 | 0 | 0.9 | 1 | 3 | 2 | 136.91 | 57.53 | 3.71 | 7.34 | 7.09 | 5.82 | 5.46 | 5.88 | -3.87 | 1 |
| tormentic acid | 13850-16-3 | C30H48O5 | 488.78 | CC1CCC2(CCC3(C(=CCC4C3(CCC5C4(CC(C(C5(C)C)O)O)C)C)C2C1(C)O)C)C(=O)O | 35 | 0 | 0.9 | 1 | 5 | 4 | 139.28 | 97.99 | 3.28 | 4.96 | 5.18 | 4.14 | 4.07 | 4.33 | -5.76 | 0 |
| 11-deoxyglycyrrhetic acid | 564-16-9 | C30H48O3 | 456.78 | CC1(C2CCC3(C(C2(CCC1O)C)CC=C4C3(CCC5(C4CC(CC5)(C)C(=O)O)C)C)C)C | 33 | 0 | 0.9 | 1 | 3 | 2 | 136.65 | 57.53 | 3.78 | 7.49 | 7.23 | 5.82 | 5.85 | 6.04 | -3.77 | 1 |
| dibenzoyl methane | 120-46-7 | C15H12O2 | 224.27 | C1=CC=C(C=C1)C(=O)CC(=O)C2=CC=CC=C2 | 17 | 12 | 0.07 | 4 | 2 | 0 | 66.35 | 34.14 | 1.94 | 3.03 | 3.14 | 2.51 | 3.65 | 2.86 | -5.52 | 0 |
| anisaldehyde | 19486-71-6 | C8H8O2 | 137.15 | COC1=CC=C(C=C1)C=O | 10 | 6 | 0.12 | 2 | 2 | 0 | 38.32 | 26.3 | 1.68 | 1.76 | 1.51 | 1.12 | 1.98 | 1.61 | -5.88 | 0 |
| wogonin | 632-85-9 | C16H12O5 | 284.26 | COC1=C(C=C(C2=C1OC(=CC2=O)C3=CC=CC=C3)O)O | 21 | 16 | 0.06 | 2 | 5 | 2 | 78.46 | 79.9 | 2.55 | 3.49 | 2.88 | 0.77 | 3.03 | 2.54 | -5.56 | 0 |
| protocatechuic acid | 99-50-3 | C7H6O4 | 154.13 | C1=CC(=C(C=C1C(=O)O)O)O | 11 | 6 | 0 | 1 | 4 | 3 | 37.45 | 77.76 | 0.66 | 1.15 | 0.8 | 0.4 | 0.26 | 0.65 | -6.42 | 0 |
| psoralen | 66-97-7 | C11H6O3 | 186.16 | C1=CC(=O)OC2=CC3=C(C=CO3)C=C21 | 14 | 13 | 0 | 0 | 3 | 0 | 52.26 | 43.35 | 2.01 | 1.67 | 2.54 | 1.48 | 2.91 | 2.12 | -6.25 | 0 |
| caffeate | 4361-87-9 | C9H8O4 | 180.17 | C1=CC(=C(C=C1/C=C\\C(=O)O)O)O | 13 | 6 | 0 | 2 | 4 | 3 | 47.16 | 77.76 | 0.89 | 1.15 | 1.09 | 0.7 | 0.75 | 0.92 | -6.58 | 0 |
| dimethylcaffeic acid | 14737-89-4 | C11H12O4 | 208.21 | COC1=C(C=C(C=C1)/C=C/C(=O)O)OC | 15 | 6 | 0.18 | 4 | 4 | 1 | 56.1 | 55.76 | 2.01 | 2.34 | 1.69 | 1.3 | 1.79 | 1.83 | -5.91 | 0 |
| vanillin | 121-33-5 | C8H8O3 | 152.15 | COC1=C(C=CC(=C1)C=O)O | 11 | 6 | 0.12 | 2 | 3 | 1 | 40.34 | 46.53 | 1.57 | 1.21 | 1.21 | 0.51 | 1.49 | 1.2 | -6.37 | 0 |
| β-amyrenyl acetate | 1616-93-9 | C32H52O2 | 468.84 | CC(=O)OC1CCC2(C(C1(C)C)CCC3(C2CC=C4C3(CCC5(C4CC(CC5)(C)C)C)C)C)C | 34 | 0 | 0.91 | 2 | 2 | 0 | 144.62 | 26.3 | 5.19 | 9.73 | 8.74 | 7.08 | 7.42 | 7.63 | -2.25 | 1 |
| formonentin | 485-72-3 | C16H12O4 | 268.28 | COC1=CC=C(C=C1)C2=COC3=C(C2=O)C=CC(=C3)O | 20 | 16 | 0.06 | 2 | 4 | 1 | 76.43 | 59.67 | 2.49 | 2.8 | 3.17 | 1.33 | 3.52 | 2.66 | -5.95 | 0 |
| aristololactam IIIa | 97399-91-2 | C16H9NO4 | 279.25 | C1OC2=C(O1)C3=C4C=C(C=CC4=CC5=C3C(=C2)C(=O)N5)O | 21 | 16 | 0.06 | 0 | 4 | 2 | 78.85 | 71.55 | 1.91 | 2.79 | 2.71 | 1.91 | 3.69 | 2.6 | -6.02 | 0 |
| 7-methoxy-2-methyl isoflavone | 19725-44-1 | C17H14O3 | 266.31 | CC1=C(C(=O)C2=C(O1)C=C(C=C2)OC)C3=CC=CC=C3 | 20 | 16 | 0.12 | 2 | 3 | 0 | 79.38 | 39.44 | 3.01 | 3.43 | 3.78 | 2.16 | 4.52 | 3.38 | -5.49 | 0 |
