# Supplementary material for: Mechanisms of Ganweikang Tablets against Chronic Hepatitis B: A Comprehensive Study of Network Analysis, Molecular Docking, and Chemical Profiling
Source: Biomed Res Int. 2023 May 8;2023:8782892. doi: 10.1155/2023/8782892 (PMC10185428; doi:10.1155/2023/8782892)
Supplement: Supplementary Materials — Figure S1: schematic diagrams for the binding modes between targets and positive control small molecules. Figure S2: the binding patterns between active ingredients, positive control, and targets. Table S1: DEG results and disease-related targets. Table S2: compound-related targets. Table S3: KEGG pathway enrichment results on each module in TPT network. Table S4: detail information of CTP network. Table S5: molecular docking results of key targets. Table S6: detail information of UPLC-QTOF/MS analysis. Table S7: detail information of GC/MS analysis. Table S8: detail information of key active ingredients. Table S9: detail information of key targets. [file 8782892.f1.zip › Table S9 Detail information of key targets.docx]

Table S9 Detail information of key targets

| Protein name | Gene name | Uniprot No. | Amino acids | Description | Category | Cellular Location | Summary |
| --- | --- | --- | --- | --- | --- | --- | --- |
| CAH2 | CA2 | P00918 | 260 | Carbonic Anhydrase 2 | Enzyme | Cytoplasm, Cell membrane | The protein encoded by this gene is one of several isozymes of carbonic anhydrase, which catalyzes reversible hydration of carbon dioxide. |
| NFKB1 | NFKB1 | P19838 | 968 | Nuclear Factor Kappa B Subunit 1 | Transcription factor | Nucleus, Cytoplasm | NF-kappa-B is a pleiotropic transcription factor present in almost all cell types and is the endpoint of a series of signal transduction events that are initiated by a vast array of stimuli related to many biological processes such as inflammation, immunity, differentiation, cell growth, tumorigenesis and apoptosis. |
| TF65 | RELA | Q04206 | 551 | RELA Proto-Oncogene, NF-KB Subunit | Transcription factor | Nucleus, Cytoplasm | NF-kappa-B is a ubiquitous transcription factor involved in several biological processes. NF-kappa-B is composed of NFKB1 or NFKB2 bound to either REL, RELA, or RELB. |
| AKT1 | AKT1 | P31749 | 480 | AKT Serine/Threonine Kinase 1 | Kinase | Nucleus, Cytoplasm, Cell membrane | This gene encodes one of the three members of the human AKT serine-threonine protein kinase family which are often referred to as protein kinase B alpha, beta, and gamma. |
| JUN | JUN | P05412 | 331 | Jun Proto-Oncogene | Transcription factor | Nucleus | This gene is the putative transforming gene of avian sarcoma virus 17. It encodes a protein which is highly similar to the viral protein, and which interacts directly with specific target DNA sequences to regulate gene expression. |
| CAH1 | CA1 | P00915 | 261 | Carbonic Anhydrase 1 | Enzyme | Cytoplasm | Carbonic anhydrases (CAs) are a large family of zinc metalloenzymes that catalyze the reversible hydration of carbon dioxide. They participate in a variety of biological processes, including respiration, calcification, acid-base balance, bone resorption, and the formation of aqueous humor, cerebrospinal fluid, saliva and gastric acid. |
| CAH6 | CA6 | P23280 | 308 | Carbonic Anhydrase 6 | Enzyme | Secreted | The protein encoded by this gene is one of several isozymes of carbonic anhydrase. This protein is found only in salivary glands and saliva and protein may play a role in the reversible hydratation of carbon dioxide though its function in saliva is unknown. |
| NEMO | IKBKG | Q9Y6K9 | 419 | Inhibitor Of Nuclear Factor Kappa B Kinase Regulatory Subunit Gamma | Disease related gene | Nucleus, Cytoplasm | This gene encodes the regulatory subunit of the inhibitor of kappaB kinase (IKK) complex, which activates NF-kappaB resulting in activation of genes involved in inflammation, immunity, cell survival, and other pathways. |
| FOS | FOS | P01100 | 380 | Fos Proto-Oncogene | Transcription factor | Nucleus, Endoplasmic reticulum Cytoplasm, Cytosol | The Fos gene family consists of 4 members: FOS, FOSB, FOSL1, and FOSL2. These genes encode leucine zipper proteins that can dimerize with proteins of the JUN family, thereby forming the transcription factor complex AP-1. |
| EP300 | EP300 | Q09472 | 2414 | E1A Binding Protein P300 | Enzyme | Cytoplasm, Nucleus, Chromosome | This gene encodes the adenovirus E1A-associated cellular p300 transcriptional co-activator protein. |
| CREB1 | CREB1 | P16220 | 327 | CAMP Responsive Element Binding Protein 1 | Transcription factor | Nucleus | This gene encodes a transcription factor that is a member of the leucine zipper family of DNA binding proteins. This protein binds as a homodimer to the cAMP-responsive element, an octameric palindrome. |
| STAT1 | STAT1 | P42224 | 750 | Signal Transducer And Activator Of Transcription 1 | Transcription factor | Nucleus, Cytoplasm | The protein encoded by this gene is a member of the STAT protein family. In response to cytokines and growth factors, STAT family members are phosphorylated by the receptor associated kinases, and then form homo- or heterodimers that translocate to the cell nucleus where they act as transcription activators. |
| MMP9 | MMP9 | P14780 | 707 | Matrix Metallopeptidase 9 | Enzyme | Secreted, extracellular space, extracellular matrix | Proteins of the matrix metalloproteinase (MMP) family are involved in the breakdown of extracellular matrix in normal physiological processes, such as embryonic development, reproduction, and tissue remodeling, as well as in disease processes, such as arthritis and metastasis. |
| CDK2 | CDK2 | P24941 | 298 | Cyclin Dependent Kinase 2 | Kinase | Nucleus, Cytoplasm | This gene encodes a member of a family of serine/threonine protein kinases that participate in cell cycle regulation. The encoded protein is the catalytic subunit of the cyclin-dependent protein kinase complex, which regulates progression through the cell cycle. |
| MDR1 | ABCB1 | P08183 | 1280 | ATP Binding Cassette Subfamily B Member 1 | Enzymes | Cytoplasm, Cell membrane | The membrane-associated protein encoded by this gene is a member of the superfamily of ATP-binding cassette (ABC) transporters. ABC proteins transport various molecules across extra- and intra-cellular membranes. |
| ABCG2 | ABCG2 | Q9UNQ0 | 655 | ATP Binding Cassette Subfamily G Member 2 | Enzymes | Cell membrane | The membrane-associated protein encoded by this gene is included in the superfamily of ATP-binding cassette (ABC) transporters. ABC proteins transport various molecules across extra- and intra-cellular membranes. |
